# Life at the periphery: what makes CHO cells survival talents

**DOI:** 10.1007/s00253-022-12123-6

**Published:** 2022-08-30

**Authors:** Tobias Jerabek, Florian Klingler, Nadja Raab, Nikolas Zeh, Jens Pfannstiel, Kerstin Otte

**Affiliations:** 1 Institute of Applied Biotechnology, University of Applied Sciences Biberach, Biberach an der Riss, Germany; 2 USP Development, Novartis AG, Kundl, Austria; 3 Cell Line Development, Bioprocess Development Biologicals, Boehringer Ingelheim GmbH & Co. KG, Biberach, Germany; 4 Core Facility Mass Spectrometry, University of Hohenheim, Stuttgart, Germany

**Keywords:** Cell surface proteome, CHO, Gene expression analysis, Survival pathway, Surfaceome, Cell characterization

## Abstract

**Abstract:**

The production of biopharmaceuticals relies on robust cell systems that can produce recombinant proteins at high levels and grow and survive in the stressful bioprocess environment. Chinese hamster ovary cells (CHO) as the main production hosts offer a variety of advantages including robust growth and survival in a bioprocess environment. Cell surface proteins are of special interest for the understanding of how CHO cells react to their environment while maintaining growth and survival phenotypes, since they enable cellular reactions to external stimuli and potentially initiate signaling pathways. To provide deeper insight into functions of this special cell surface sub-proteome, pathway enrichment analysis of the determined CHO surfaceome was conducted. Enrichment of growth/ survival-pathways such as the phosphoinositide-3-kinase (PI3K)–protein kinase B (AKT), mitogen-activated protein kinase (MAPK), Janus kinase/signal transducers and activators of transcription (JAK-STAT), and RAP1 pathways were observed, offering novel insights into how cell surface receptors and ligand-mediated signaling enable the cells to grow and survive in a bioprocess environment. When supplementing surfaceome data with RNA expression data, several growth/survival receptors were shown to be co-expressed with their respective ligands and thus suggesting self-induction mechanisms, while other receptors or ligands were not detectable. As data about the presence of surface receptors and their associated expressed ligands may serve as base for future studies, further pathway characterization will enable the implementation of optimization strategies to further enhance cellular growth and survival behavior.

**Key points:**

• *PI3K/AKT, MAPK, JAK-STAT, and RAP1 pathway receptors are enriched on the CHO cell surface and downstream pathways present on mRNA level.*

• *Detected pathways indicate strong CHO survival and growth phenotypes.*

• *Potential self-induction of surface receptors and respective ligands.*

**Graphical abstract:**

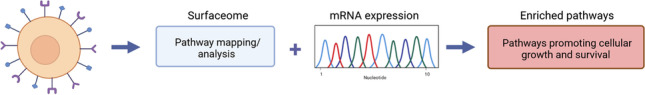

**Supplementary Information:**

The online version contains supplementary material available at 10.1007/s00253-022-12123-6.

## Introduction

The production of biopharmaceuticals relies on robust cell systems that can produce recombinant proteins at high levels and grow and survive in the stressful bioprocess environment. Various factors, like shear force, nutrient and serum deprivation, hypoxia, or increased pH and osmolality, could compromise these cellular abilities (Lim et al. 2010). Chinese hamster ovary cells (CHO) offer a variety of advantages including robust growth in suspension and their tolerance of variations in pH, oxygen levels, and temperature or pressure as well as the ability to be adapted to grow in defined serum-free media and the availability of genetic tools for cellular modification (Kim et al. 2012; Lai et al. 2013). Due to their economic importance and the continuously increasing demand for more complex biopharmaceuticals, there has been a significant interest to further optimize growth behavior, stress tolerance, and productivity by testing media composition and additives (Ehret et al. 2019), optimizing process conditions (Handlogten et al. 2018; McHugh et al. 2020; Alhuthali et al. 2021; Torres et al. 2021), or by genetic engineering (Lim et al. 2010; Tan et al. 2015; MacDonald et al. 2022).

Omics studies are a recent method to gain further insight into complex cellular processes. As a recent addition to various proteomic studies of CHO cells (Kantardjieff et al. 2010; Baycin-Hizal et al. 2012; Heffner et al. 2017; Kol et al. 2020), the cell surface proteome (surfaceome) of CHO cells was uncovered (Klingler et al. 2021). Since cells receive and record substantial information by their surface proteins, this particular sub-proteome is of special interest to understand how CHO cells react to their environment. Recorded signals on the cellular surface are further transmitted to the intracellular compartments via cellular signaling where they lead to various reactions like induction of growth, cell cycle progression, or apoptosis determining the fate of the cell.

There are several signaling pathways known to be responsible for regulation of cellular growth and survival. Among them, a central and highly conserved enzymatic cascade for maintenance and regulation of cell metabolism, growth, cell cycle, and survival is the phosphoinositide-3-kinase (PI3K)–protein kinase B (AKT). Activation of PI3K-AKT pathway signaling depends among others on membrane bound and associated activating receptor tyrosine kinases (RTKs). Binding of growth factors on RTKs on the cell surface promotes activation of class 1A PI3Ks starting to phosphorylate phosphatidylinositol (3,4)-biphosphate (PIP_2_) to phosphatidylinsositol (3,4,5)-triphospahte (PIP_3_). This process induces activation of AKT which promotes numerous downstream functions (Hemmings and Restuccia 2012). In addition, mitogen-activated protein kinase (MAPK) family members are essential for regulating cellular functions like proliferation, differentiation, development, stress response, and apoptosis. Cascades of MAPK kinase kinases (MAPKKK) activated by growth factors, cytokines, or stress, and a downstream MAPK kinase (MAPKK) and MAPK activation, are essential for cellular responses to external stimuli. Three mammalian MAPK families are known, the classical MAPK known as extracellular signal-regulated kinase (ERK), C-Jun N-terminal kinase/stress-activated protein kinase (JNK/SAPK), and p38 kinase (Zhang and Liu 2002; Morrison 2012). Cellular proliferation, differentiation, migration, apoptosis, and survival are also regulated via the Janus kinase/signal transducers and activators of transcription (JAK-STAT) pathway as reaction to cytokines like interleukin (IL) 6 and growth factors like epidermal growth factor (EGF). Components of JAK-STAT pathway interact with proteins involved in ERK MAPK and PI3K signaling (Harrison 2012). Finally, RAP1 signaling pathway records extracellular signals by G-protein coupled receptors (GPCRs). RAP1 is a member of the Ras family GTPases, which are known to regulate many cellular processes like cell growth, apoptosis, adhesion, and intracellular vesicular transport. Furthermore, downstream signaling of RAP1 pathway is a known activator of ERK MAPK and PI3K-AKT pathways (Jaśkiewicz et al. 2018).

The current study is based on the recently determined CHO surfaceome dataset, which was linked to transcription analysis data and analyzed for growth and survival relevant pathways with the scope of increasing knowledge about characteristics of CHO cells. Resulting datasets enable an increased understanding of how CHO cell reacts to their stressful bioprocess environment and maintain phenotypes of robust growth and survival during cell culture conditions. In addition, our analyses provide a knowledge base for the identification of novel potential engineering targets or media supplement approaches for improvement of CHO production cells grown under bioprocess conditions.

## Methods

### Proteomic analysis

The sub-proteome of cell surface proteins was characterized using the cell surface capturing (CSC) method for labeling proteins on the surface (Wollscheid et al. 2009). Experiments with CHO cells were conducted as previously reported (Klingler et al. 2021). In brief, various different CHO cell lines were labeled with biotin on *N*-glycan moieties present on cell surface proteins. After cellular lysis proteins were digested using trypsin to render peptides, biotin-labeled glycopeptides were enriched by streptavidin–biotin affinity chromatography and released via PNGaseF cleavage of the *N*-linked glycan. Enriched glycopeptides were analyzed by nano-LC–ESI–MS/MS to identify proteins present on the cell surface by unique peptide sequences. The mass spectrometry proteomics data have been deposited to the ProteomeXchange Consortium via the PRIDE (Perez-Riverol et al. 2022) partner repository with the dataset identifier PXD033581 and 10.6019/PXD033581.

### Relative gene expression analysis

Mean relative gene expression values of CHO-DG44-mAb1 were determined as described previously (Raab et al. 2022). Shortly, total RNA was isolated from cells in twelve replicates using miRNeasy Mini Kit (Qiagen, Hilden, Germany). Isolated RNA was used for library generation and sequencing. Resulting data were mapped to CHO genome (CriGri_1.0, GCA_000223135) and normalized to library size and analyzed with GeneData Selector® software (Genedata, Basel, Switzerland). Erythropoietin (EPO), which is known to be not expressed in CHO (Chen et al. 2001), showed a relative expression value of − 1.02; thus, genes with expression values of ≤ 0 were considered as not expressed. For comparison, the housekeeping gene glycerinaldehyd-3-phosphat-dehydrogenase (GAPDH) showed a relative expression value of 13.35. The relative gene expression data are included in the supplementary information files of the article (Supplemental Table S1-4).

### Bioinformatic analyses

DAVID 2021 (https://david.ncifcrf.gov/) (Dennis et al. 2003) and Panther (http://pantherdb.org/) (Mi et al. 2019a,b) online tools were used for Gene Ontology annotation and classification and pathway enrichment analysis. Panther generic mapping tool (ftp://ftp.pantherdb.org/generic_mapping) was used for mapping hamster protein sequences to usable IDs for Panther online classification tool. Kyoto Encyclopedia of Genes and Genomes (KEGG) Automatic Annotation Server (KAAS) (https://www.genome.jp/kegg/kaas/) (Moriya et al. 2007) was used for custom KEGG pathway mapping of identified hamster protein sequences.

## Results

### Analysis of the CHO surfaceome identifies enrichment of survival and growth inducing receptors

**Fig. 1:**
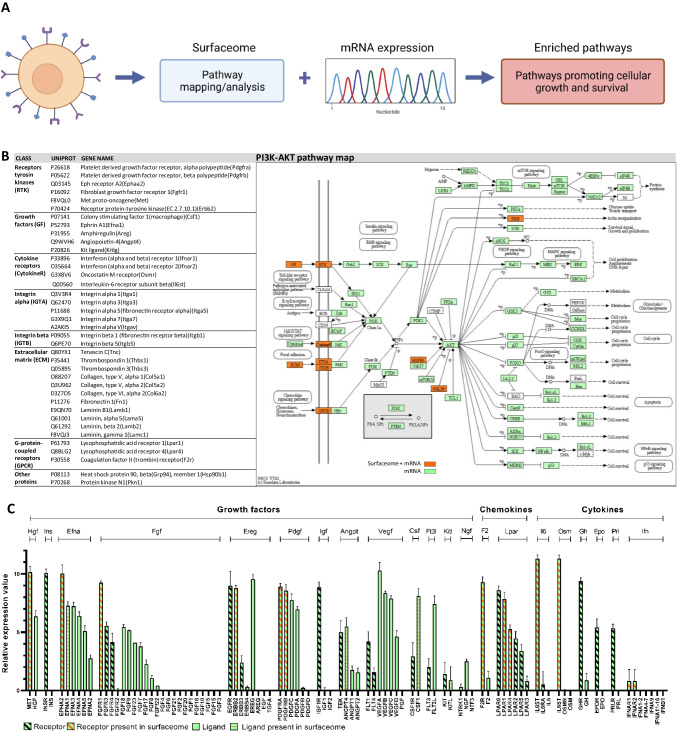
Surfaceome and transcriptomic analysis of CHO cells. **a** Workflow of the characterization of cells. Pathway analysis was performed based on surfaceome data. Enriched growth-related pathways were further evaluated using mRNA expression data. Created with BioRender.com. **b** Identified surface receptors of the PI3K/AKT pathway in the surfaceome (left) and their annotation in the KEGG PI3K/AKT pathway (ko04151) (right). Proteins identified in the surfaceome and on mRNA level are marked in orange. Proteins only identified on mRNA level are marked in green. The figure was modified from the Kyoto Encyclopedia of Genes and Genomes (KEGG) database with permission (Kanehisa and Goto 2000; Kanehisa 2019; Kanehisa et al. 2021). **c** Mean relative expression values of identified KEGG-annotated surface and secreted proteins of the PI3K/AKT in CHO-DG44-mAb1 with standard deviation (SD). Receptors are marked with a diagonal line. Orange color indicates the presence of the protein in the surfaceome data set. An orange horizontal line indicates the presence of a ligand in the surfaceome data set

Proteins embedded in and associated with the membrane facilitate essential biological functions that enable the survival of a cell by uptake of nutrients or signaling functions in response to external stimuli and conditions. In a previous proteomic study, we uncovered the cell surface proteome of various CHO cell lines using the CSC method (Wollscheid et al. 2009) leading to the identification of 449 unique proteins present on the cellular surface (Klingler et al. 2021). Based on this data set, pathway enrichment analysis using the KEGG database and gene ontology (GO) identified enrichment of surface proteins belonging to pathways promoting cellular growth and survival (Fig. 1a). Enriched pathways included the PI3K-AKT pathway, MAPK pathway, and JAK-STAT pathway as well as RAP1 pathway. Those pathways are associated with a variety of effects, such as regulation of different mechanisms like apoptosis, cell cycle, and proliferation. Comparable enrichment of growth- and survival-related pathways was observed for surface proteins of human embryonic kidney 293 (HEK293) cell lines, which are also utilized as fast-growing production cell lines (Bausch-Fluck et al. 2015) (Table 1). However, in contrast to this surfaceome, datasets obtained from other cell lines provided in other studies like the slow-growing plasma cell–derived myeloma cell line JK-6L (Burger et al. 1994) (unpublished dataset from our own laboratory) or braintumor cells (Bausch-Fluck et al. 2015) did not display a similar enrichment of proteins involved in growth- and survival-related pathways (Table 1).

**Table 1:** Enriched growth/survival-related pathways in the CHO surfaceome in comparison to other cell lines. Number of identified surface proteins (*p*-value/Benjamini Hochberg correction) involved in survival/growth-related pathways, according to KEGG annotation and enrichment analysis results according to DAVID 2021. Datasets for cell lines used for comparison were obtained from previous studies: JK-6L (unpublished dataset), braintumor, and HEK293 cells (Bausch-Fluck et al. 2015)

Pathway	CHO	JK-6L	Braintumor	HEK293
PI3K/AKT	20 (7.1E−21/5.9E−19)	15 (5.3E−3/7.2E−2)	14 (1.1E−5/2.3E−4)	34 (1.1E−6/3.3E−5)
MAPK	11 (2.8E−5/2.5E−4)	5 (7.8E−1/1.0E0)	-	16 (1.4E−1/5.4E−1)
JAK-STAT	7 (3.9E−2/1.2E−1)	-	-	8 (4.1E−1/1.0E0)
RAP1	8 (3.6E−6/4.3E−5)	7 (1.9E−1/9.9E−1)	-	16 (1.3E−2/1.1E−1)

### Receptors of the PI3K-AKT pathway enriched on CHO cells to induce growth and survival phenotypes

Detailed analysis of the identified surface proteins revealed a subset of 20 out of 72 surface receptors of the PI3K/AKT pathway to be present and detectable on CHO cells (Fig. 1b). These included several receptors of the class receptors tyrosine kinases (RTK), cytokine receptors (CytokineR), integrin alpha and beta (ITGA & ITGB), and GPCR. Only the receptor class of toll-like receptors 2/4 (TLR 2/4), the B-cell receptor (BCR), and cluster of differentiation 19 (CD19) could not be detected on the surface of CHO cells. In addition, 16 ligands binding to the detected receptors were identified, including growth factors (GF) and extra cellular matrix (ECM) proteins (Fig. 1b). In addition, interleukin 6 cytokine family signal transducer (IL6ST), which is not annotated in the PI3K/AKT KEGG pathway, but plays an important role as signal transducer and receptor subunit (Kishimoto et al. 1994), was detected in the surfaceome and therefore included in the dataset.

In order to evaluate whether the identified receptors and ligands would be able to induce intracellular downstream signaling cascades, the surfaceome data set was supplemented with RNA expression data. For most proteins of the PI3K/AKT signaling cascade located downstream of the surface receptors, mRNA expression was detectable, suggesting regulation of cellular processes including protein synthesis, apoptosis, and cell cycle as well as other cellular pathways including the NFkB and p53 signaling pathways (Fig. 1b).

In order to extend the study to even weakly expressed or technically undetectable surface receptors on protein level, we analyzed the relative mRNA expression values of all KEGG described surface receptors and associated ligands within the PI3K/AKT pathway (Fig. 1c). While additional 15 receptors of the RTK class were found to be expressed on mRNA level, also most of their corresponding ligands were expressed with only insulin (INS) and insulin-like growth factor-1/2 (IGF1/2) being not detectable on mRNA level (Fig. 1c). Within the class of GPCRs, 4 additional receptors were expressed on mRNA level. However, only coagulation factor II receptor (F2R) was found to be co-expressed with its associated ligand coagulation factor II (F2), since it is the only mRNA encoded ligand. Some cytokine receptors require various numbers of subunits to form the functional receptor complex. While the growth hormone receptor (GHR), erythropoietin receptor (EPOR), and prolactin receptor (PRLR) are functional without subunits, the interleukin 6 receptor alpha (IL6RA) and oncostatin M receptor (OSMR) both require IL6ST to form a functional receptor complex. Also, interferon alpha receptor 1 and 2 (IFNAR1/2) need to form a dimer to be functional. In sum, 4 additional and functional receptors were present on mRNA level (Fig. 1c). However, with the exception of growth hormone (GH), no cytokine receptor ligands were co-expressed. Furthermore, 13 additional integrin receptors containing 9 receptors of the ITGA class and 4 receptors of the ITGB class were identified on mRNA level (Supplemental Fig. S1). These data indicate that a large amount of surface receptors as well as associated ligands of the PI3K/AKT pathway are present in CHO cells which may therefore be active to induce growth- and survival-related phenotypes.

### Interconnected MAPK pathway enriched on CHO cells

**Fig. 2:**
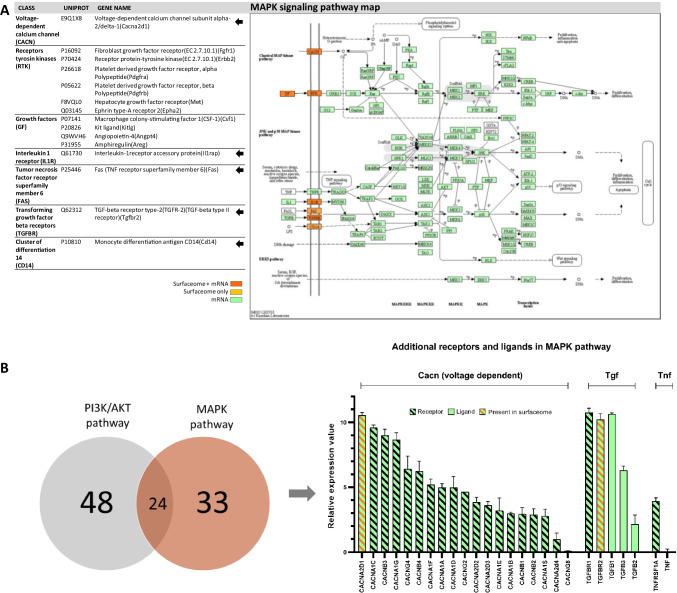
Analysis of proteins in the MAPK pathway. **a** Identified surface receptors of the MAPK pathway in the surfaceome with arrows as indication for exclusive proteins for the MAPK pathway (left) and annotation of all identified proteins in the KEGG MAPK pathway (ko04010) (right). Proteins identified in the surfaceome and on mRNA level are marked in orange. Proteins only identified in the surfaceome are marked in yellow. Proteins only identified on mRNA level are marked in green. The figure was modified from the KEGG database with permission (Kanehisa and Goto 2000; Kanehisa 2019; Kanehisa et al. 2021). **b** Comparison of KEGG annotated surface and secreted proteins of the PI3K/AKT pathway and MAPK pathway (left). Proteins exclusive to the MAPK pathway were analyzed on mRNA level and plotted with their mean relative expression values of identified proteins of the PI3K/AKT in CHO-DG44 with SD. Receptors are marked with a diagonal line. Orange color indicates the presence of the protein in the surfaceome data set

In addition to the growth, proliferation, and survival-promoting PI3K/AKT pathway, the MAPK pathway was enriched in KEGG and GO analysis of the surfaceome data set (Table 1). In sum, 15 proteins were identified by our proteomics approach (Fig. 2a). Since the PI3K/AKT and MAPK pathways are well described to be interconnected (Cao et al. 2019), 10 of these proteins were common to both pathways and only 5 receptors were exclusive for the MAPK pathway (Fig. 2a, marked with arrows). These include surface receptors of the classes calcium voltage-gated channels (CACN) and IL1R, as well as transforming growth factor beta receptors (TGFBR), receptor tumor necrosis factor receptor superfamily member 6 (FAS), and CD14. The 10 proteins being in common with the PI3K/AKT pathway were part of the RTKs as well as respective associated ligands described above. RTKs as well as CACNs are involved in signaling of the ERK cascade of the MAPK pathway, which is associated survival-promoting effects (Balmanno and Cook 2009).

Upon complementing the surfaceome data set with mRNA expression data, all involved proteins downstream of the surface receptors were found to be expressed on mRNA level. This indicates a possible regulation of apoptosis and cell cycle regulation, proliferation, and differentiation as well as the regulation of the Wnt and p53 signaling pathways within CHO cells (Fig. 2a).

Based on the described interconnection of the PI3K/AKT and MAPK pathways, we compared all KEGG annotated receptors for these pathways and identified 24 receptors to be present in both pathways, 48 to be specific for the PI3K/AKT pathway and 33 receptors to be specific for the MAPK pathway (Fig. 2b). Analysis of mRNA expression of these additional 33 MAPK pathway specific receptors identified 18 out of 26 voltage dependent CACN receptors to be expressed on mRNA level (Fig. 2b). In addition, all annotated TGFBRs were co-expressed with their respective ligands. Finally, tumor necrosis factor receptor superfamily member 1A (TNFRSF1A) expressed on mRNA level in contrast to its ligand tumor necrosis factor (TNF).

### Enriched Jak-Stat and Rap1 pathways as additional activators for PI3K/AKT and MAPK signaling pathways

**Fig. 3:**
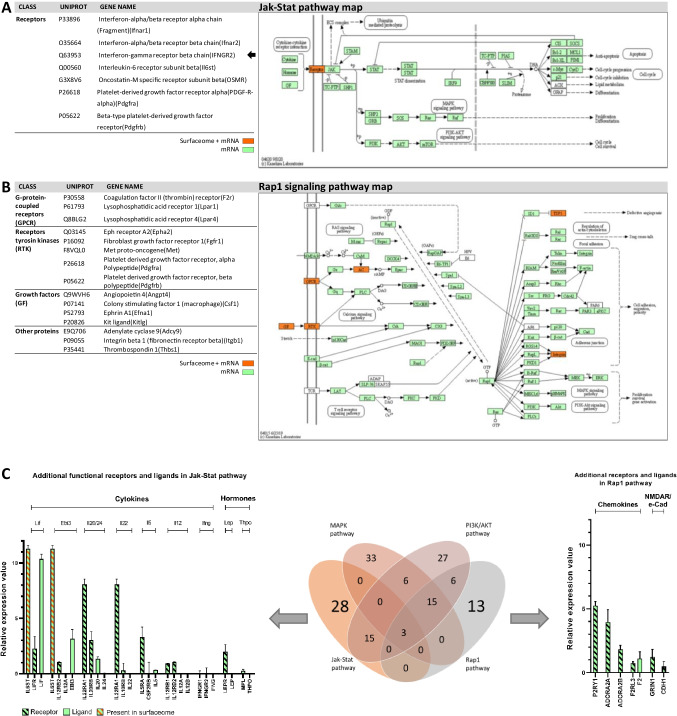
Analysis of proteins in the JAK-STAT and RAP1 pathways. **a** Identified surface receptors of the Jak-Stat pathway in the surfaceome with arrows as indication for exclusive proteins for the MAPK pathway (left) and annotation of all identified proteins in the KEGG JAK-STAT pathway (ko04630) (right). Proteins identified in the surfaceome and on mRNA level are marked in orange. Proteins only identified on mRNA level are marked in green. The figure was modified from the KEGG database with permission (Kanehisa and Goto 2000; Kanehisa 2019; Kanehisa et al. 2021). **b** Identified surface receptors of the MAPK pathway in the surfaceome (left) and annotation of all identified proteins in the KEGG MAPK pathway (ko04015) (right). Proteins identified in the surfaceome and on mRNA level are marked in orange. Proteins only identified on mRNA level are marked in green. The figure was modified from the KEGG database with permission (Kanehisa and Goto 2000; Kanehisa 2019; Kanehisa et al. 2021). **c** Comparison of KEGG annotated surface and secreted proteins of the PI3K/AKT pathway, MAPK pathway, JAK-Stat pathway, and RAP1 pathway (middle). Proteins exclusive to the JAK-STAT pathway (left) and proteins exclusive to the RAP1 pathway (right) were analyzed on mRNA level and plotted with their mean relative expression values of identified proteins of the PI3K/AKT in CHO-DG44-mAb1 with SD. Receptors are marked with a diagonal line. Orange color indicates the presence of the protein in the surfaceome data set

In addition to the MAPK and PI3K/AKT pathways, KEGG and GO analysis identified an additional enrichment of surface receptors for the JAK-STAT and the RAP1 pathways, which both are described to activate the PI3K/AKT and MAPK pathways (Fig. 3a, b). Furthermore, the JAK-STAT pathway serves as additional regulator of apoptosis and cell cycle (Fig. 3a). For the JAK-STAT pathway, 7 receptors were identified in the surfaceome dataset. One receptor was exclusive for the JAK-STAT pathway and 6 were also present in the PI3K/AKT pathway (Fig. 3a, marked with arrows). The exclusive receptor was member of the CytokineR class. For the RAP1 pathway, 15 receptors were identified in the surfaceome; however, all of those are also present in the PI3K/AKT pathway. mRNA expression analysis of both pathways showed that all downstream signaling proteins of the receptors were expressed, allowing them to serve as additional activators of the PI3K/AKT and MAPK pathways (Fig. 3a,b).

Since the described cellular signaling pathways share several surface receptors, we compared all KEGG annotated surface receptors of all pathways to identify common and unique proteins (Fig. 3c). While only 3 receptors were common to all pathways, the MAPK and AKT pathways are induced by 33 and 27 unique receptors, respectively, followed by JAK-STAT (28) and RAP1 pathways (13).

Considering the 28 exclusive receptors of the JAK-STAT pathway, 15 receptors were expressed on mRNA level; however, only leukemia inhibitory factor (LIF), IL2a, IL5, and IL20 were expressed ligands for functional receptor complexes. On the other hand, IL22, IL12 a/b, interferon gamma (IFNG), leptin (LEP), and thrombopoietin (THPO) were unexpressed ligands to functional receptor complexes. For the RAP1 pathway, 6 of the 13 unique receptors were found to be expressed on mRNA level. The transcriptomic data revealed additional surface receptors, such as glutamate ionotropic receptor NMDA type subunit 1 (GRIN1) of the class *N*-methyl-D-aspartate receptors (NMAR) and cadherin 1 (CDH1), the only annotated member of the call of E-cadherins (E-CAD). However, with the exception of F2 serving as ligand for coagulation factor II receptor-like 3 (F2RL3), and CDH1 working via homophilic interaction, no expression statement of ligands could be made, as they are not encoded by a specific mRNA.

## Discussion

CHO cells continue to be the most frequently used mammalian expression system in the biopharmaceutical industry (Walsh 2018). This is due to the fact that they offer a wide range of advantages, including their ability to grow in suspension and defined serum-free media, as well as their tolerance of harsh bioprocess conditions, caused for example by shear stress, nutrient, or oxygen depletion (Jayapal et al. 2007; Lim et al. 2010; Lai et al. 2013). External stimuli during bioprocessing are recorded by surface proteins on the cell membrane and signals further transmitted into the cell leading to various cellular reactions. Knowledge about presence of surface proteins and understanding of induced intra-cellular signaling cascades therefore present a valuable source for understanding and improvement of CHO as expression system. Thus, we aimed to expand the available sets of CHO omics data and supply subsequent analysis of survival-related surface receptor enrichment on the surface of CHO cells to potentially serve as knowledge base for future approaches to optimize biopharmaceutical production with CHO cells.

The presented analysis of CHO surfaceome datasets identified an enrichment of surface proteins associated with anti-apoptotic effects, cell proliferation, or cell growth. A similar enrichment of proteins involved in these pathways was identified on HEK293 cells, another commonly used cell line for biopharmaceutical production. In contrast to that, proteins involved in these pathways were not as dominantly enriched on the slow-growing cell line JK-6L or braintumor cells. These data suggest these pathways and involved surface proteins to potentially correlate with a fast and robust growth phenotype, which is highly desirable during bioprocessing conditions.

The PI3K/AKT pathway, as one of the enriched pathways, is known to act as a central cellular regulator, being associated with anti-apoptotic effects as well as promotion of cell proliferation, cell growth, or modulation of metabolism (Yao and Cooper 1995; Bao et al. 2004; Porta et al. 2014; Manning and Toker 2017). Previous efforts in CHO cell engineering have already focused on this pathway by overexpression of active AKT, which induced bioprocess relevant and beneficial effects as reduced apoptosis and decreased level of autophagosome accumulation (Hwang and Lee 2009). Furthermore, overexpression of oncogenic mutant phosphatase and tension homology deleted on chromosome ten (PTEN), a negative regulator of AKT, resulted in enhanced proliferation, reduced apoptosis rate, and increased transient recombinant protein expression (Zhou et al. 2021) and the overexpression of EGF receptor in CHO cells resulted in higher sensitivity to EGF supplementation and higher growth rates in the presence of EGF (Chen et al. 2001). On the other hand, medium supplementation may be a relevant strategy based on expressed or missing ligands of identified surface receptors. Previous studies have shown that for example further addition of already expressed ligands of the PI3K/AKT, MAPK, and RAP1 pathways as autocrine growth factors, fibroblast growth factor (FGF) 8, hepatocyte growth factor, and vascular endothelial growth factor C induced positive effect on CHO cell growth (Lim et al. 2013). However, since further addition of the already expressed cytokine LIF to growth medium resulted in inhibition of cell growth in CHO cells (Lim et al. 2013), the outcome of pathway modulation might not easily be predictable and in favor for optimized bioprocesses. In order to evaluate the expression and presence of relevant ligands for the identified surface receptors in CHO cells, the surfaceome data set was supplemented with RNA expression data. Thereby, severall expressed surface receptors with no co-expressed respective ligand could be identified in this study. Among those, the probably most prominent receptor was the insulin receptor, which was detectable on RNA level, but not its ligand insulin. This is in accordance with the need for supplementation of insulin to CHO cells cultured in serum-free culture media to prevent apoptosis and enhance cell viability (Adamson and Walum 2007). Furthermore, a combination of insulin and the additional growth factor basic FGF has been reported to enhance cell growth and recombinant protein synthesis in CHO (Liu and Wu 2009). An alternative supplement for serum-free cell culture of CHO cells is IGF-1 (Ross and Englesberg 1993), which again was not detectable on RNA level in our study, while the corresponding receptors were expressed. Following this rationale, further studies could focus on weakly or non-expressed ligands associated with present surface receptors identified in this study such as EPO or PRL, for culture medium supplementation and subsequent assessment of potential beneficial effects on the cells. However, elevated non-physiological concentrations of IGF-1 also have been reported to inhibit cell proliferation and reduce productivity (Romand et al. 2016), highlighting that ligand supplementation or overexpression approaches need to be conducted carefully. Following the opposite approach, weakly or non-expressed surface receptors such as FGFR2 may be overexpressed to enhance sensitivity of the cells to the respective ligands, as already demonstrated for epidermal growth factor receptor (Chen et al. 2001).

Moreover, our analysis revealed the presence of several surface receptors with co-expression of their associated ligands, in particular for growth factors including different vascular endothelial growth factors (VEGFs). This suggests the possibility for autocrine self-induction of the cells, which might lead to activation of survival-related pathways resulting in the ability to sustain harsh bioprocess conditions. The concept of autocrine self-induction of survival pathways has been demonstrated in previous studies, where for example a hypoxia-induced increase of VEGF expression prevented apoptosis in serum-deprived tumor cells (Baek et al. 2000). Due to the increased expression of VEGF under hypoxic/serum-deprived conditions, the authors suggested that VEGF might act as a self-promoting survival factor.

Furthermore, VEGF-induced anti-apoptotic effects were reported to be caused by activation of the ERK cascade of the MAPK pathway (Baek et al. 2000), a pathway which was also enriched in the CHO surfaceome data set. The MAPK pathway is divided into three different cascades, called ERK, p38, or JNK cascade. The ERK cascade is generally considered to promote cell survival, especially in the context of tumors (Balmanno and Cook 2009). For example, a dominant effect of activated ERK1/2 was reported to be able to protect cells from apoptotic signaling via death receptors (Tran et al. 2001). Since our study mainly identified surface receptors of the ERK cascade, which are also in common with the PI3K/AKT pathway, CHO cells may primarily induce the survival-promoting ERK cascade. However, while the ERK cascade is generally associated with anti-apoptotic effects and the p38 as well as the JNK cascades are mostly associated with pro-apoptotic effects, there are also examples of opposite effects, indicating the relevance of the respective molecular context (Yue and López 2020). Therefore, CHO production cells will have to be analyzed in great detail for the roles of the different cascades of the MAPK pathway prior to and during modulation of culture conditions or genetic engineering, since cells might modulate the molecular context and therefore change the effect of different MAPK cascades.

As an additional activator of the survival-associated pathways, the JAK-STAT pathway was enriched in the surfaceome data set. Despite its ability to induce the PI3K/AKT and MAPK pathways, it also promotes additional proliferative and anti-apoptotic effects (Vainchenker and Constantinescu 2013), potentially additionally contributing to the observed robust growth and survival phenotypes of CHO cells.

Lastly, the RAP1 pathway was enriched in the CHO surfaceome, which also is interconnected with the PI3K/AKT and MAPK pathways (Jaśkiewicz et al. 2018). However, the exact effect of RAP1 is dependent on the cell type (Jaśkiewicz et al. 2018) and even in similar cell types the effect of RAP1 might differ (Tsygankova et al. 2001; Lou et al. 2002). Thus, further analysis of the role of RAP1 in CHO cells might be necessary to reveal a better understanding of its cellular role in order to be beneficial for bioprocess optimization.

In conclusion, our study provides novel insights into the cell surface proteome of CHO cells and how cell surface receptors and ligand-mediated signaling may enable the cells to support growth and survival in a bioprocess environment. As data about the presence of surface receptors and their associated expressed ligands may serve as base for future studies, further pathway characterization is needed to enable and implement novel optimization strategies to further enhance cellular growth and survival behavior.

## Supplementary Information

Below is the link to the electronic supplementary material.

Supplementary file1 (PDF 1896 KB)

## Data Availability

The mass spectrometry proteomics data have been deposited to the ProteomeXchange Consortium via the PRIDE (Perez-Riverol et al. 2022) partner repository with the dataset identifier PXD033581 and 10.6019/PXD033581. Proteomic datasets from HEK293 and braintumor cells are accessible via the surface protein atlas (https://wlab.ethz.ch/cspa/). Proteomic datasets of JK-6L cells are currently unpublished proprietary data available upon request. The relative gene expression data are included in the supplementary information files of the article (Table S1-4).
