# Supplementary material for: Life at the periphery: what makes CHO cells survival talents
Source: Appl Microbiol Biotechnol. 2022 Aug 30;106(18):6157–67. doi: 10.1007/s00253-022-12123-6 (PMC9468092; doi:10.1007/s00253-022-12123-6)
Supplement: Supplementary file 1 — Supplementary file1 (PDF 1896 KB) [file 253_2022_12123_MOESM1_ESM.pdf]

Applied Microbiology and Biotechnology

Life at the periphery: What makes CHO cells survival talents

Tobias Jerabek[#,1,*], Florian Klingler[1,*], Nadja Raab[1,2], Nikolas Zeh[1,3], Jens Pfannstiel[4] and Kerstin Otte[1]

[1]: Institute of Applied Biotechnology, University of Applied Sciences Biberach, Biberach an der Riss, Germany

[2]: USP development, Novartis AG, Kundl, Austria

[3]: Cell Line Development, Bioprocess Development Biologicals, Boehringer Ingelheim GmbH & Co. KG, Biberach, Germany.

[4]: Core Facility Mass Spectrometry, University of Hohenheim, Stuttgart, Germany

[*]: Both authors contributed equally to this manuscript.

[#]: Corresponding author: E-Mail: jerabek@hochschule-bc.de; Phone: +49 (0) 7351 582-455

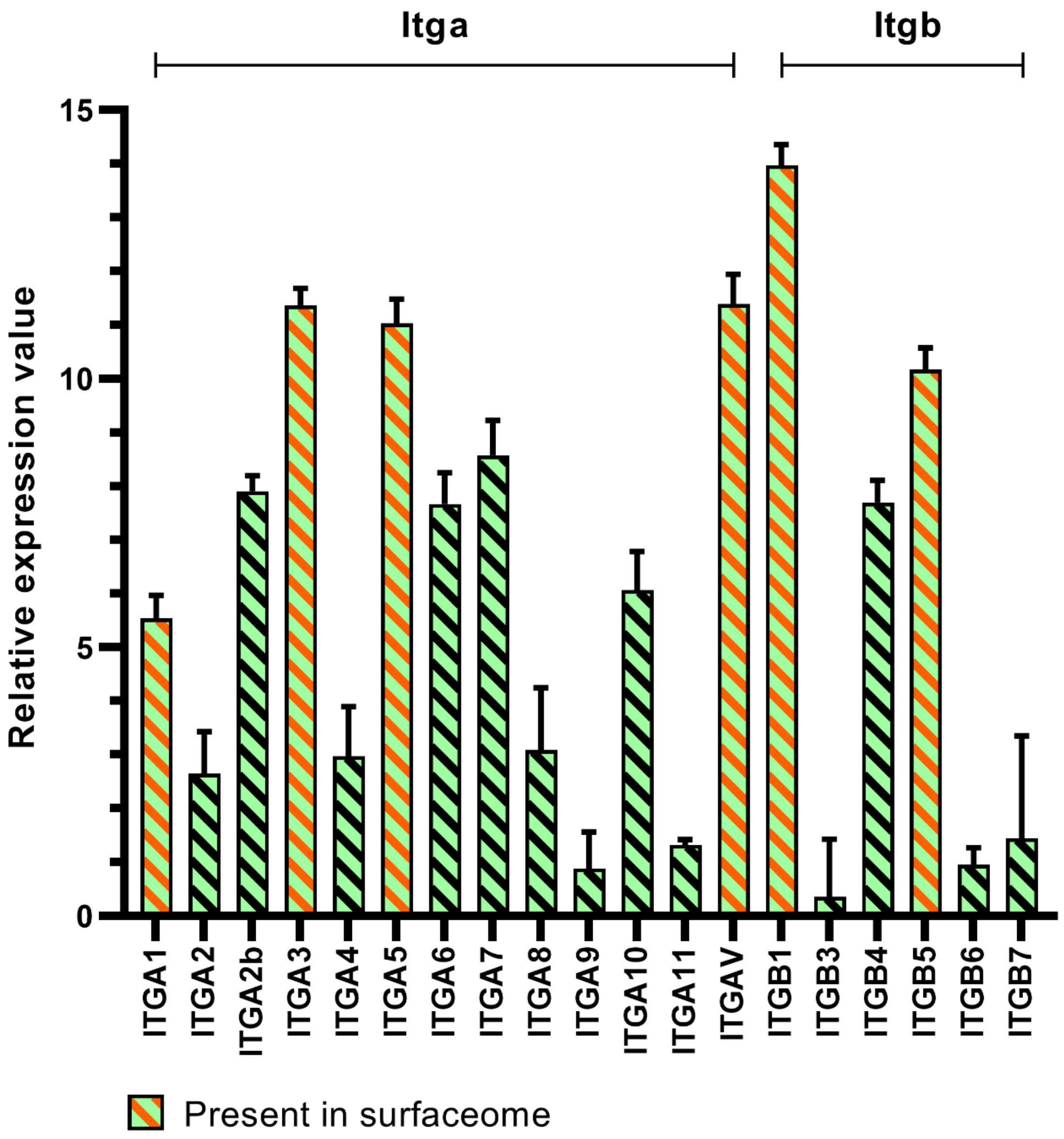

Fig. S1 Identified integrin receptors of the PI3K/AKT pathway

Mean relative expression values of identified KEGG-annotated integrin receptors of the PI3K/AKT in CHO-DG44-mAb1 with standard deviation (SD). Orange color indicates the presence of the protein in the surfaceome data set.

Table S1 Relative expression values of genes related to the PI3K-AKT pathway

PI3K-AKT	Replicate 1	Replicate 2	Replicate 3	Replicate 4	Replicate 5	Replicate 6	Replicate 7	Replicate 8	Replicate 9	Replicate 10	Replicate 11	Replicate 12	Mean	SD	Comment
GAPDH	13.5720	13.8087	13.5775	12.9286	12.6895	12.9789	13.4230	13.6322	13.6013	13.3205	13.3560	13.2579	13.3455	0.3331	Positive Reference Gen
EPO	-1.0478	-1.0043	-1.0477	-1.0476	-1.0476	-0.9545	-1.0477	-1.0478	-1.0477	-1.0477	-0.9653	-0.9802	-1.0238	0.0370	Negative Reference Gen
HSP90B1	16.5944	16.4856	16.7656	16.7881	16.4450	16.7995	16.2145	16.3084	16.2905	16.5389	16.6648	17.2067	16.5918	0.2771	
LAMB1	14.0092	13.7774	14.0648	13.7120	13.5529	13.7558	14.1548	13.9881	14.0929	14.0295	14.0777	14.7571	13.9977	0.3029	
ITGB1	14.4833	14.2941	14.5779	13.6967	13.4304	13.8708	13.9368	13.8733	13.9475	13.5261	13.5959	14.4074	13.9700	0.3876	
LAMC1	13.6409	13.3610	13.7045	12.6194	12.6374	12.9773	13.4319	13.2016	13.2827	13.1451	13.2275	14.2815	13.2926	0.4582	
MYC	12.9329	12.9820	12.9262	12.9637	12.8473	12.9155	13.3571	13.3971	13.3457	12.8349	12.8262	12.6471	12.9980	0.2391	
THBS1	13.5517	13.1452	13.5569	11.9202	12.0535	12.1904	12.9619	12.6039	12.8268	12.8505	12.8744	13.9511	12.8739	0.6247	
COL5A1	12.1286	11.9996	12.2793	12.6247	12.7138	12.7056	13.0181	12.8461	12.9156	13.0163	12.9950	13.4825	12.7271	0.4221	
ATF4	12.5945	12.9625	12.4914	12.9263	12.6672	12.9056	12.5733	12.6845	12.7573	12.5026	12.5696	12.3154	12.6625	0.1965	
HSP90AA1	13.2112	13.1589	13.2784	11.9315	12.0038	12.1039	13.0115	13.0777	12.9076	12.0812	12.1516	12.6859	12.6336	0.5352	
CHUK	12.3029	12.1274	12.2604	12.5130	12.6068	12.5045	12.3905	12.2782	12.3756	12.5135	12.4418	12.2148	12.3774	0.1440	
LAMB2	11.9783	11.9456	11.9998	12.6155	12.7048	12.5712	12.5939	12.4598	12.5915	12.1978	12.1595	12.2475	12.3388	0.2809	
COL5A2	12.4027	12.2230	12.5192	12.0927	12.1545	12.3150	12.2421	11.9582	12.1124	12.0194	12.0676	12.8096	12.2430	0.2408	
GNB1	12.2615	12.3523	12.1680	12.0891	11.9739	12.1999	12.0902	12.0951	11.9404	12.3201	12.3219	12.3993	12.1843	0.1501	
FN1	12.4974	12.2731	12.7002	11.1037	11.2482	11.5333	12.4586	12.2036	12.2805	11.6378	11.7167	12.8089	12.0385	0.5703	
RAC1	11.4810	11.5909	11.4031	11.7228	11.7026	11.7549	11.5017	11.5636	11.5211	11.2911	11.3533	11.0922	11.4982	0.1930	
PTK2	11.1904	11.1404	11.1836	11.7832	11.7172	11.7599	11.5634	11.5035	11.5291	11.5831	11.5632	11.1806	11.4748	0.2394	
ITGAV	10.8863	10.5965	11.0219	11.6986	11.7722	11.8257	11.0738	10.9227	10.9744	11.7740	11.7232	12.4404	11.3925	0.5479	
ITGA3	11.1582	10.9770	11.1954	11.6719	11.7112	11.7704	11.1751	11.0395	11.1098	11.3209	11.3413	11.8996	11.3642	0.3156	
IL6ST	11.2228	10.9270	11.3525	11.1044	11.3553	11.1060	11.2427	11.1333	11.1967	11.3824	11.4496	12.1697	11.3035	0.3095	
COL6A1	10.4714	10.2536	10.5855	11.4031	11.5525	11.5119	11.2537	11.1020	11.1715	11.7133	11.7594	12.3111	11.2574	0.5917	
ATF6B	11.5035	11.3340	11.5520	11.1560	10.9318	11.1658	11.2803	11.2609	11.2665	10.9477	10.9790	11.5240	11.2418	0.2174	
YWHAE	11.7421	11.6462	11.6487	10.6544	10.6071	10.8175	11.7295	11.7357	11.4038	10.8059	10.7707	11.2363	11.2332	0.4697	
JAK1	11.5773	11.4257	11.5887	10.6087	10.7113	10.7186	11.4969	11.4465	11.3317	10.8689	10.8874	11.3319	11.1662	0.3744	

CRTC2	10.7623	10.7677	10.7627	11.9310	11.9056	11.9725	10.9336	10.8127	10.9624	11.1445	11.1373	10.8370	11.1608	0.4858
GNB2	11.1346	11.2998	11.1474	11.1745	10.9832	11.1031	11.0861	11.1617	11.0915	11.0989	11.0977	11.1434	11.1268	0.0738
PKN2	10.9307	10.8361	10.9020	11.3005	11.3729	11.1830	11.0693	10.9830	11.0551	11.0461	11.0250	10.8143	11.0432	0.1724
ITGA5	10.7623	10.5219	10.8323	10.7124	10.7554	10.7892	11.1024	10.9670	11.0248	11.2594	11.3079	12.2606	11.0246	0.4529
PPP2R1A	11.1277	11.1995	11.0945	10.7225	10.6148	10.8490	10.9306	10.9278	10.8773	10.9202	10.9771	11.0035	10.9370	0.1645
THBS3	9.9440	9.8437	9.8951	11.4634	11.5202	11.3988	10.7147	10.5699	10.7412	11.4736	11.4913	11.2306	10.8572	0.6691
EIF4B	11.2161	11.2311	11.1846	10.4700	10.4823	10.2356	11.0922	11.0545	11.1218	10.7279	10.6677	10.7783	10.8552	0.3405
STK11	10.6070	10.6365	10.5689	10.9773	11.0369	11.0660	10.9263	10.8110	10.8665	10.6223	10.6131	10.3939	10.7605	0.2151
RPTOR	10.9058	10.8038	10.9227	10.6166	10.8360	10.5838	10.7417	10.5505	10.7019	10.6720	10.6086	10.7085	10.7210	0.1242
MTOR	10.5973	10.5724	10.6838	10.6982	10.8748	10.7847	10.7279	10.5986	10.7657	10.6691	10.5977	10.6774	10.6873	0.0905
TSC2	10.2045	10.1179	10.1499	11.3081	11.3409	11.3044	10.4940	10.4618	10.4833	10.8250	10.8109	10.5219	10.6685	0.4499
LAMA2	10.2283	9.9748	10.3159	10.5457	10.6031	10.6804	10.4649	10.3637	10.4450	11.1244	11.0978	11.4971	10.6117	0.4310
CDK4	10.8589	10.9650	10.8646	10.1505	10.1917	10.3218	10.6975	10.7785	10.5632	10.4782	10.4834	10.7545	10.5923	0.2715
YWHAG	10.6339	10.5947	10.5712	10.0441	10.1013	10.2710	10.4988	10.5021	10.4154	10.6465	10.7333	11.0586	10.5059	0.2773
GNG12	10.4733	10.3992	10.3948	10.4484	10.6226	10.3424	10.5108	10.2932	10.2469	10.6665	10.5469	10.7365	10.4735	0.1504
CDC37	10.7574	10.7150	10.7010	9.6919	9.9236	10.0247	10.5255	10.5187	10.4060	10.3576	10.3580	10.8790	10.4049	0.3630
FOXO3	10.0309	9.9260	10.0177	10.8159	10.8928	10.6972	10.0543	10.0129	10.0517	10.5533	10.5217	10.5800	10.3462	0.3616
TEP1	9.8439	9.8577	9.8936	10.7975	11.0158	10.8214	10.2837	10.2177	10.2268	10.3634	10.3984	10.1813	10.3251	0.3853
VEGFA	9.7805	9.7162	9.8433	11.4271	11.4914	11.2323	9.6843	9.5422	9.7503	10.3565	10.3363	10.0384	10.2666	0.7194
PKN1	9.9809	10.0001	9.9900	10.5365	10.6633	10.5084	10.1885	10.0471	10.1524	10.3123	10.3189	10.3582	10.2547	0.2329
MDM2	10.1723	10.0757	10.0917	10.5120	10.5512	10.4856	10.1271	10.0624	10.0557	10.1114	10.1928	10.0655	10.2086	0.1908
EIF4EBP1	10.7636	10.8237	10.6749	9.3488	9.2029	9.6543	10.4171	10.4330	10.2716	10.1508	10.2497	10.4630	10.2045	0.5334
GYS1	9.9079	9.8183	9.9080	10.5423	10.6420	10.5471	10.0735	9.9767	10.0132	10.2464	10.2562	10.2685	10.1834	0.2791
CCND1	10.5411	10.5323	10.5715	9.2709	9.4322	9.2866	10.7715	10.7688	10.6291	10.0733	9.9975	10.3254	10.1834	0.5685
ITGB5	10.3982	10.1422	10.4697	9.7338	9.6852	10.0536	10.6380	10.5184	10.5838	9.6195	9.6746	10.5334	10.1709	0.4015
MET	10.2139	9.8769	10.3383	9.4042	9.5582	9.6294	10.7528	10.6375	10.6722	9.9925	9.9368	10.8485	10.1551	0.4976
RAF1	10.0856	10.0764	10.0724	10.1959	10.3417	10.2593	10.3624	10.1845	10.1787	10.0367	9.9888	10.0595	10.1535	0.1208
IKBKB	9.5529	9.3989	9.5995	10.8800	10.7881	10.7766	9.8436	9.8612	9.9876	10.5475	10.5252	10.0178	10.1482	0.5288
INSR	10.2067	9.8869	10.2950	9.9354	10.0969	9.9412	9.7433	9.6800	9.7487	10.1873	10.2082	10.9359	10.0721	0.3415
JAK3	9.1505	9.3149	9.2970	10.7481	10.8176	10.6045	10.0211	9.9810	10.1439	10.3945	10.3180	9.7725	10.0470	0.5707

PKN3	9.5710	9.5153	9.6146	10.4621	10.4342	10.3765	10.1803	10.0390	10.1910	10.1870	10.2275	9.5239	10.0269	0.3676
EPHA2	10.5958	10.3902	10.6848	8.7759	8.7455	9.0197	10.3555	10.2023	10.3339	10.0302	10.0438	11.0603	10.0198	0.7634
SOS1	9.6752	9.6241	9.6611	10.2777	10.2333	10.2617	9.8577	9.7789	9.7355	10.1170	10.1319	10.0824	9.9530	0.2544
PRKAA1	9.7747	9.6756	9.8135	10.1522	10.4058	10.1060	10.1217	9.9799	10.1605	9.7422	9.6793	9.6884	9.9417	0.2443
AKT1	9.8190	9.7996	9.7243	10.0171	9.9920	10.0747	10.0875	9.8846	9.8976	9.7995	9.7503	9.7493	9.8830	0.1305
CDKN1A	9.6458	9.7824	9.5567	10.0933	9.9158	10.1105	9.5875	9.5179	9.4519	10.0203	10.0793	10.1111	9.8227	0.2598
SGK1	10.0514	9.9824	10.1123	10.0583	10.1385	10.0550	10.2268	10.3910	10.2066	8.7938	8.7515	8.8190	9.7989	0.6187
COL6A2	9.5893	9.4280	9.7719	9.3846	9.4213	9.7617	9.8248	9.6880	9.8327	10.0031	10.0789	10.7347	9.7932	0.3702
COL3A1	10.4642	10.3240	10.6364	9.1113	9.1775	9.5677	10.1242	9.9816	10.0909	8.9282	9.0414	10.0296	9.7897	0.5985
GNB4	9.8448	9.7155	9.8137	9.6114	9.6723	9.5943	10.0228	9.9532	9.9177	9.7561	9.7757	9.7557	9.7861	0.1323
NFKB1	9.7781	9.7069	9.8188	9.4854	9.6087	9.6568	9.7400	9.6161	9.5476	10.0923	10.0524	10.3081	9.7843	0.2470
RHEB	9.8611	9.8948	9.6956	9.6011	9.4475	9.6105	9.7537	9.8769	9.6461	9.9587	9.8541	9.9044	9.7587	0.1577
GRB2	9.8187	9.8374	9.8203	9.8006	9.6244	9.7788	9.8335	9.8058	9.7379	9.5354	9.6914	9.7674	9.7543	0.0936
CCND3	9.5641	9.5821	9.5033	9.6188	9.4847	9.6795	9.7073	9.7372	9.6409	9.8482	9.8109	10.1526	9.6941	0.1829
ATF2	9.4606	9.5301	9.5085	10.0978	10.0912	10.0961	9.6065	9.4562	9.5388	9.7094	9.6488	9.5017	9.6871	0.2568
MAPK1	10.0027	9.9077	9.9221	9.5429	9.5066	9.4020	9.8633	9.7680	9.6867	9.2965	9.3009	9.3329	9.6277	0.2630
RELA	9.6768	9.6231	9.5984	9.1628	9.3076	9.3082	9.6839	9.5520	9.4918	9.9192	9.8013	10.2676	9.6161	0.2976
MCL1	9.7999	9.8368	9.6727	9.5187	9.7423	9.2923	9.6099	9.5605	9.6360	9.4362	9.3583	9.4518	9.5763	0.1722
RPS6KB1	9.3684	9.2808	9.3754	9.9423	9.8965	9.8434	9.5552	9.4692	9.6267	9.5710	9.5487	9.2558	9.5611	0.2324
EREG	10.1187	9.9371	10.1513	9.1945	8.8706	9.0163	9.2268	9.1994	9.4183	9.7434	9.7694	9.8909	9.5447	0.4425
CDKN1B	9.2918	9.3445	9.2546	9.5584	9.5720	9.6845	9.4648	9.5296	9.5753	9.2679	9.4054	9.3987	9.4456	0.1401
YWHAB	9.4422	9.3989	9.4160	9.4315	9.6377	9.4092	9.5160	9.5198	9.3861	9.3757	9.3925	9.2895	9.4346	0.0885
GHR	9.1481	9.0026	9.2189	9.7590	9.8151	9.8556	9.2013	9.1967	9.2409	9.4806	9.5224	9.4400	9.4068	0.2847
PPP2R5A	9.0664	9.0558	9.0678	9.8660	9.9612	9.8873	9.2830	9.1864	9.1933	9.4766	9.4551	9.3287	9.4023	0.3338
JAK2	9.3697	9.3379	9.3876	9.2426	9.3210	9.4307	9.6172	9.5477	9.5603	9.3032	9.2775	9.3329	9.3940	0.1206
MAP2K2	9.4592	9.5844	9.4184	9.2923	9.4514	9.3256	9.5387	9.4778	9.4350	9.2165	9.1145	9.3919	9.3921	0.1344
CDK2	9.1672	9.1644	9.1959	9.6473	9.7126	9.4444	9.6545	9.7062	9.7283	9.1574	9.1632	8.8369	9.3815	0.3025
F2R	9.5960	9.4303	9.7191	8.8500	8.5320	9.0493	9.6668	9.6148	9.6981	8.8268	8.7184	9.7368	9.2865	0.4551
FGFR1	9.1670	8.9461	9.2649	9.2462	9.2356	9.3703	9.2159	9.1531	9.1281	9.1745	9.2275	9.7014	9.2359	0.1777
RBL2	8.5930	8.5622	8.8854	9.8777	9.9450	9.7273	9.1974	9.1110	8.9031	9.0313	8.9867	9.2109	9.1692	0.4598

PIK3CA	8.8941	8.7973	8.9463	9.3578	9.5141	9.3783	9.0681	9.0091	8.9539	9.3110	9.1101	9.0802	9.1184	0.2225
PHLPP1	9.1265	9.0544	9.0427	8.9274	8.9183	8.8629	9.3452	9.3513	9.2526	8.9781	8.9524	9.3343	9.0955	0.1816
EGFR	7.8056	7.6686	7.8223	11.1868	9.8082	10.9622	8.2306	7.9687	8.2839	9.6179	9.4639	8.7251	8.9620	1.2320
PHLPP2	8.8026	8.6236	8.7862	9.0211	9.0156	8.7874	9.0959	8.8513	8.9183	9.0520	8.9082	9.1118	8.9145	0.1496
PDGFRA	8.7241	8.4761	8.7502	9.0801	9.0547	9.1926	9.0553	8.9531	9.0290	8.5550	8.5331	9.3950	8.8999	0.2888
PIK3CB	8.9997	8.7413	8.8727	8.6727	8.8009	8.6105	9.2069	9.0518	8.8863	8.9648	8.8451	9.1312	8.8987	0.1807
IGF1R	8.9767	8.7371	9.1688	8.2517	8.6963	8.1217	9.4528	9.3262	9.3846	8.7205	8.6269	9.0556	8.8766	0.4284
ERBB2	8.7272	8.4591	8.7276	8.9371	9.0450	9.1235	8.9295	8.6419	8.8685	8.5930	8.5841	8.9226	8.7966	0.2047
LAMA3	9.1033	8.9633	9.2803	8.1102	8.2693	8.3176	9.3904	9.3750	9.3922	8.2457	8.3055	8.4015	8.7628	0.5277
GNB3	7.9344	7.8599	7.7381	10.0100	9.8752	9.8745	8.0234	7.8651	8.0223	9.5323	9.4797	8.7186	8.7445	0.9335
PPP2R3A	8.7490	8.5799	8.5960	8.3783	8.5897	8.5269	8.7546	8.6585	8.6672	8.9105	8.9908	9.2517	8.7211	0.2349
PIK3R1	8.6038	8.4967	8.5200	8.8517	8.7943	8.8142	8.9309	8.8709	8.8672	8.5985	8.5085	8.7161	8.7144	0.1604
BCL2L1	8.9053	8.9913	8.8030	8.1724	8.1704	8.4201	8.8138	8.7811	8.6396	8.8015	8.8143	9.1500	8.7052	0.3050
DDIT4	8.8309	8.9704	8.8649	8.4215	8.2985	8.3902	8.9469	9.0481	8.9563	8.3884	8.4184	8.2805	8.6512	0.3051
LPAR6	8.8184	8.6847	8.8934	8.4266	8.4345	8.3276	8.9187	9.0362	9.0441	8.3614	8.3413	7.8632	8.5959	0.3603
TSC1	8.2929	8.1169	8.3746	8.6215	9.1079	8.6776	8.5891	8.4236	8.6299	8.7174	8.6507	8.7931	8.5829	0.2568
NRAS	8.4296	8.3021	8.5704	8.6368	8.9355	8.4326	8.8141	8.5569	8.6609	8.5084	8.5073	8.5541	8.5757	0.1716
ITGA7	7.6659	7.5435	7.7331	8.9273	9.0371	8.9862	8.4087	8.2710	8.4423	9.3318	9.1409	9.3313	8.5682	0.6542
PDGFRB	7.9081	7.6439	7.9983	8.4990	8.4875	8.5429	8.7159	8.7071	8.7307	8.8450	8.9618	9.6879	8.5607	0.5376
PRKCA	8.7510	8.5813	8.7480	8.1014	8.6334	8.0322	8.8775	8.8874	8.8351	8.2237	8.1733	8.6475	8.5410	0.3185
VEGFB	8.1480	8.2159	8.1707	8.5631	8.2418	8.6062	8.1490	8.4060	8.1513	8.5438	8.4290	8.4300	8.3379	0.1771
SGK3	8.1763	8.0445	8.1130	7.8734	8.2724	8.1806	8.5821	8.5229	8.4692	8.0541	8.0229	7.9262	8.1865	0.2321
GNB5	7.6194	7.5932	7.7053	8.6763	8.8148	8.6450	7.8928	7.9397	7.8649	8.4836	8.3294	8.4351	8.1666	0.4438
CSF1	7.6546	7.4047	7.7612	7.4425	7.5966	7.4906	8.2458	8.2866	8.1387	8.8076	8.8536	9.5481	8.1026	0.6794
CREB1	8.1445	8.0342	8.1772	8.1882	8.3366	7.9417	8.2700	8.1083	8.2063	7.8977	7.8655	8.0250	8.0996	0.1486
CDK6	8.4047	8.0376	8.3842	7.3083	7.4922	6.8421	8.5162	8.4750	8.4456	7.9822	7.9976	8.4471	8.0277	0.5454
GSK3B	8.1936	8.1988	8.1300	7.8716	7.9845	7.7395	8.1119	8.2480	8.1165	7.6495	7.5164	7.8882	7.9707	0.2384
ITGA2B	7.4733	7.5226	7.5364	8.2032	8.2516	8.1719	7.9847	8.0022	7.9794	8.1617	7.9771	7.5700	7.9028	0.2944
VEGFC	8.3031	8.3215	8.4201	7.5995	7.6946	7.5540	7.7655	7.8188	7.8107	7.5469	7.6635	7.9141	7.8677	0.3110
LPAR1	8.3126	7.9780	8.2824	7.3036	6.8225	7.3167	8.4714	8.4995	8.4068	7.2964	7.2892	8.0869	7.8388	0.5916

PDGFC	8.1474	8.0114	8.2657	7.0321	6.8760	7.2214	8.2479	8.3075	8.2457	7.3824	7.2963	7.7752	7.7341	0.5388
IRS1	7.8720	7.6248	7.9608	6.7813	7.0180	6.9696	8.3931	8.4048	8.3635	7.7041	7.4761	8.0844	7.7210	0.5686
ITGB4	7.2180	7.1797	7.1508	8.2377	8.3216	8.2886	7.6843	7.5591	7.6049	7.7029	7.7351	7.5944	7.6898	0.4120
PDPK1	7.7439	7.6134	7.9145	7.4488	7.7615	6.8684	8.1251	7.8855	7.8918	7.5153	7.3346	7.9109	7.6678	0.3392
ITGA6	7.8388	7.5233	8.0401	6.9931	6.9071	6.8368	8.2279	7.9165	7.7914	7.5111	7.5407	8.8452	7.6643	0.5824
COL4A1	8.3013	8.2550	8.2558	6.3686	6.5613	6.1353	8.7559	8.4878	8.5141	7.2071	7.2882	7.2931	7.6186	0.9241
PIK3CG	6.4789	6.3289	6.2544	8.0458	8.1382	7.8954	7.3233	7.1925	7.2743	8.9751	8.9213	8.2416	7.5891	0.9368
HRAS	7.4140	7.5140	7.3319	7.7922	7.5789	8.0662	7.1767	7.4505	7.4151	7.2996	7.4219	7.4002	7.4884	0.2368
CCNE1	8.0995	7.8569	7.8823	6.8591	6.6445	6.8693	8.0374	7.7954	7.9716	7.0469	7.0628	7.1049	7.4359	0.5456
FLT3L	6.4100	6.5775	6.5084	8.1535	8.2579	8.2127	7.1124	6.8882	7.2605	7.8517	8.0281	7.7047	7.4138	0.7051
IKBKG	7.2934	6.9957	7.3384	7.1022	7.5674	7.0720	7.6337	7.2355	7.4488	7.5654	7.5270	7.7636	7.3786	0.2444
KRAS	7.4382	7.4450	7.7496	7.1054	7.4338	7.0881	7.5359	7.6554	7.7291	7.1562	6.9418	7.1529	7.3693	0.2734
EFNA1	7.0577	6.8238	7.0950	7.7223	7.6476	7.9223	6.9110	7.0473	6.9742	7.2594	7.3066	7.3331	7.2584	0.3461
CCND2	7.5130	7.0938	7.6809	6.8046	7.3311	6.6743	7.4737	7.4172	7.0909	7.3664	6.7548	7.6142	7.2346	0.3447
EFNA3	6.7881	6.9772	6.8918	7.7464	7.6904	7.7768	6.9795	7.1610	7.2287	7.1090	7.1708	7.1839	7.2253	0.3356
RXRA	6.4101	6.5435	6.3620	7.3663	7.4684	7.2250	7.1982	7.2260	7.0212	7.3528	7.4414	7.7382	7.1128	0.4442
PDGFA	6.6595	6.6203	6.6195	7.3033	7.2371	7.1159	6.7536	6.8338	6.9157	7.2603	7.1255	7.1333	6.9648	0.2608
MLST8	6.7453	6.8748	7.0725	6.9800	7.0101	6.7831	7.3614	7.1191	7.0725	6.7730	6.6516	6.7567	6.9334	0.2050
GNG5	6.8413	7.2345	6.8534	7.1065	6.5955	6.9271	6.6351	6.9817	6.8395	6.9143	7.0185	7.0532	6.9167	0.1830
NR4A1	6.1747	6.4518	6.3408	7.1425	7.1376	7.0797	6.6813	6.6176	6.6949	7.3446	7.2657	7.1283	6.8383	0.3925
GNG11	6.4376	6.7499	6.2838	6.8784	6.7385	6.8679	6.4084	6.7595	6.2147	7.0354	7.2121	7.0084	6.7162	0.3158
BDNF	7.0519	6.7964	7.0678	6.5326	6.0925	6.4568	6.6489	6.7931	6.4455	6.0644	6.2084	6.1881	6.5289	0.3512
PIK3R3	6.2277	6.0967	6.3126	6.5672	6.4259	6.1771	6.4692	6.4297	6.4149	7.0156	6.6220	6.7702	6.4607	0.2584
EFNA4	6.0418	5.9046	5.9291	7.0521	6.8172	6.6720	6.2893	6.3558	6.4465	6.4357	6.5616	6.3323	6.4032	0.3460
HGF	6.9725	6.7967	6.8641	6.0684	5.8991	6.1470	6.7261	6.3519	6.5987	5.8745	5.3533	6.7558	6.3673	0.5008
GNG8	5.0080	5.2685	5.3157	7.1489	6.9823	7.0900	5.9439	5.7275	5.9953	6.9720	6.7886	6.1978	6.2032	0.7774
ITGA10	5.3590	5.0966	5.2387	6.7709	6.7185	6.6193	5.5179	5.4207	5.7195	6.9188	6.9278	6.3854	6.0578	0.7237
VWF	4.9955	4.7073	4.7961	5.5972	6.6963	6.1234	5.7965	5.4005	5.6240	7.7439	7.5597	7.3069	6.0289	1.0662
GNG10	6.3752	6.2378	6.0379	5.1634	5.4897	5.8052	6.1456	6.1533	5.8668	5.8667	6.0075	6.3261	5.9563	0.3517
MTCP1	5.2625	5.2605	5.4166	5.9301	6.2821	5.9630	5.7774	5.3470	5.6678	5.9161	6.0775	5.7449	5.7205	0.3362

FGFR3	5.2883	5.1666	5.1787	5.9480	5.8127	6.0614	5.5358	5.3093	5.1882	5.7754	5.6403	5.7087	5.5511	0.3184
ITGA1	5.8417	5.3084	5.6609	5.0228	5.1870	4.9957	5.3113	5.5388	5.8349	5.8165	5.4176	6.4955	5.5359	0.4254
ANGPT4	4.9014	4.8998	4.9744	4.8433	4.4968	4.5045	6.0776	6.0685	6.1136	5.9911	5.9444	6.8120	5.4690	0.7749
FGF18	5.4785	5.7564	5.4444	5.5463	5.5614	5.6129	5.2693	5.2671	5.1824	5.2311	5.3472	5.7394	5.4530	0.1961
EPOR	4.9448	4.8728	4.8708	6.2295	6.1592	6.1894	4.5646	4.4316	4.6860	6.0033	6.2849	5.6051	5.4035	0.7378
PRLR	5.2842	5.1700	5.5179	4.9902	4.8006	4.9262	5.7095	5.6794	6.0457	5.3366	5.2019	5.2733	5.3280	0.3593
LPAR4	5.6319	5.2831	5.8445	5.0483	5.1436	4.8623	5.6083	5.5141	5.3655	4.6568	4.6770	5.3594	5.2496	0.3822
FGF9	5.1356	5.1425	5.1356	5.1524	5.1512	5.1520	5.1446	5.1356	5.1548	5.1592	5.2472	5.1583	5.1558	0.0301
RELN	4.4878	4.2495	4.5737	5.9966	6.1369	6.0101	4.3998	4.7849	3.9159	5.6565	6.0091	5.4974	5.1432	0.8172
EFNA5	5.5005	5.0250	5.5140	4.5666	4.7080	4.3203	5.5543	5.2932	5.1074	5.0955	4.5173	5.8556	5.0881	0.4802
CHAD	4.3649	4.3286	4.6654	5.3954	5.9005	5.1026	5.4660	5.2825	5.2768	5.0837	4.7748	5.0754	5.0597	0.4610
TEK	3.9995	4.2114	3.9940	6.6351	6.5289	6.0084	4.3390	4.1966	4.5574	5.4896	5.4365	4.7504	5.0122	0.9738
AKT2	4.7455	4.7343	4.9098	5.0925	5.6626	4.2852	5.2252	5.2498	5.3050	5.1339	4.8486	4.7178	4.9925	0.3603
EIF4E	5.0193	4.8454	5.0813	5.1228	4.7485	5.1446	4.9965	4.9661	4.8662	5.0151	5.1736	4.6442	4.9686	0.1636
LAMA5	4.7162	4.2453	4.5178	4.8155	5.2720	4.1548	5.5462	4.7945	4.9064	4.6467	4.5644	5.7626	4.8285	0.4860
IL7	4.3487	4.3383	4.4969	4.9293	4.8595	5.1858	4.8642	4.8225	4.7976	4.9747	5.0906	4.9894	4.8081	0.2751
PIK3R2	3.6289	4.6680	4.3328	5.2908	5.1629	5.5391	5.0030	4.3836	4.3267	4.6624	4.1408	4.6177	4.6464	0.5367
PIK3AP1	4.5400	3.6665	4.0158	4.2214	4.6647	4.4458	4.7245	4.5488	4.7691	5.1977	5.1317	5.7212	4.6373	0.5489
VEGFD	3.9474	4.0894	3.9265	5.1260	4.5331	4.7595	4.4150	4.2028	5.0016	5.3319	5.1375	4.9825	4.6211	0.5014
LPAR2	3.4218	3.9750	3.6213	4.9367	5.0695	4.6489	4.1313	4.1528	4.3491	4.8135	5.3617	4.8759	4.4465	0.6011
BCL2	4.7531	4.4508	4.9800	4.2104	3.5989	1.3262	5.4267	5.0220	4.7935	4.7071	4.6780	5.1889	4.4280	1.0859
CREB5	4.3976	3.8022	4.3288	3.8943	3.6557	3.1984	5.0251	4.8572	4.7969	4.8069	4.5275	5.5215	4.4010	0.6615
PPP2R2B	4.3960	4.3959	4.3961	4.3967	4.4203	4.3966	4.3960	4.3960	4.3961	4.3963	4.3965	4.3962	4.3982	0.0069
COL1A1	3.1109	3.1479	3.2271	5.0143	5.3428	5.4719	3.8752	4.1208	4.3729	4.6805	4.9322	4.3455	4.3035	0.8327
SYN1	3.9327	3.7828	4.1510	4.5381	4.6886	4.3376	3.9626	4.0141	3.9123	4.6521	4.7069	4.8314	4.2925	0.3755
NOS3	3.5367	3.3590	3.4702	5.3983	5.2605	5.2418	3.3147	3.3828	3.7358	5.1142	4.9364	4.5560	4.2755	0.8754
FLT1	3.9782	3.6107	3.8575	4.1140	4.0968	4.0784	4.0004	3.7485	3.9565	4.1514	4.2171	6.7571	4.2139	0.8196
FGFR4	3.6743	3.5239	3.7393	5.2257	4.9507	4.8286	3.3600	3.2425	3.3720	4.7022	4.8432	4.6625	4.1771	0.7470
FGF23	4.0844	4.0843	4.0845	4.0851	4.0850	4.0851	4.0845	4.0844	4.0845	4.0847	4.0849	4.0847	4.0847	0.0003
CSF3	3.9153	4.0267	4.1164	3.0987	3.2838	3.0057	4.6006	4.2432	4.7796	4.6057	4.6770	4.3425	4.0579	0.6227

BRCA1	4.1405	4.0290	4.2631	3.9581	3.8788	3.6224	3.8994	4.0176	3.8063	4.1175	3.0687	4.0729	3.9062	0.3130
MAP2K1	3.9547	3.9289	4.1485	4.1052	4.2472	3.4520	4.1970	3.9633	3.9114	3.6063	3.6553	3.6886	3.9049	0.2544
SGK2	3.2980	3.2968	3.4642	3.6004	4.2052	3.8373	3.8454	3.5961	4.1760	4.2295	4.2114	4.2383	3.8332	0.3743
LAMC2	3.2218	2.9290	3.3050	3.5675	3.6763	3.7903	3.6917	4.0414	3.7501	4.3122	4.7923	4.7203	3.8165	0.5697
FGF7	4.1441	3.9250	4.3831	3.3315	3.5170	3.3995	3.6470	3.6958	3.8711	4.1620	3.7446	3.5239	3.7787	0.3273
AKT3	3.2145	2.7738	2.6244	3.9002	3.8221	3.3277	3.8829	3.9698	3.5171	4.5937	4.1699	4.0045	3.6501	0.5773
PIK3R5	2.4634	1.8573	3.3145	4.0762	4.2825	3.7752	3.8006	3.5212	2.9966	4.4902	4.4115	4.5246	3.6261	0.8443
PIK3CD	1.9712	2.6169	2.7965	4.1764	4.8065	4.6224	2.8806	3.6242	3.6488	4.1906	3.6600	3.2838	3.5232	0.8528
LPAR5	3.2311	3.3866	2.4439	4.3793	3.9337	4.3505	2.7050	3.3250	3.1466	3.1224	3.5477	3.0806	3.3877	0.5910
CASP9	3.3634	2.8263	3.2518	3.1084	3.8352	3.9929	3.4932	2.8831	2.6129	2.9939	2.9551	3.4055	3.2268	0.4130
ITGA8	2.4302	1.6276	2.9505	4.5833	1.9451	3.4508	2.4717	1.6187	2.5546	4.3123	4.3817	4.6959	3.0852	1.1615
LAMB3	2.0256	2.2569	2.2951	2.2731	2.5008	2.6479	2.5581	2.2307	1.8169	4.2838	4.3235	6.4907	2.9753	1.3706
ITGA4	1.4548	2.1092	2.4318	4.5618	3.1999	2.0454	2.6271	2.5989	3.6558	3.6363	4.1865	3.0959	2.9669	0.9260
CSF1R	1.4583	1.8618	1.8323	2.9973	4.3021	4.2000	2.5538	1.7181	2.3706	3.7073	3.2951	4.9759	2.9394	1.1622
COL2A1	2.1232	2.4649	2.0634	3.6167	3.0808	3.3823	1.8349	1.9841	2.0590	4.2522	4.1722	3.9815	2.9179	0.9325
GNG3	2.2632	2.5640	2.2003	3.2625	3.4066	3.2416	2.3898	2.5555	2.6189	3.3494	3.2691	3.1557	2.8564	0.4624
EFNA2	2.6704	2.4220	2.3126	3.0278	3.0814	2.7756	3.0882	2.9553	2.3112	3.0742	2.8233	2.4866	2.7524	0.3048
ITGA2	1.6644	1.7983	2.0201	2.7941	4.3245	3.0417	2.5192	2.1636	2.1331	3.3414	3.4216	2.5571	2.6483	0.7790
NGF	2.3688	2.3530	2.3552	2.1207	2.5454	2.3227	2.6541	2.7229	2.6157	2.6579	2.7167	2.6511	2.5070	0.1950
ERBB3	1.1641	2.0160	1.7173	3.0343	3.4412	2.5183	2.6130	2.5870	2.5056	2.4881	2.3896	2.4501	2.4104	0.5832
BCL2L11	2.1352	1.5835	2.9346	2.8002	3.4375	2.3925	1.8124	1.7865	2.5164	2.7280	1.7039	2.6858	2.3764	0.5769
SPINT2	0.8643	0.6594	0.9742	3.1013	3.0189	2.8258	2.6111	2.3813	2.6893	3.3803	3.2952	2.6709	2.3727	0.9739
FGF17	2.3007	1.8706	2.2278	2.7509	2.7077	1.7358	1.7222	2.4972	2.0866	2.6599	2.2156	2.4213	2.2664	0.3610
TNC	2.2668	2.2477	2.0234	1.9883	2.3145	1.3506	1.9538	1.7854	2.9842	2.6685	2.3807	2.6277	2.2160	0.4366
FLT3	2.0402	0.9765	0.7026	2.1785	2.9982	2.8799	2.6946	1.1731	1.6380	2.1247	2.3058	2.3133	2.0021	0.7410
NTF5	1.9918	1.7927	1.8705	1.7951	2.0234	1.7950	1.7932	1.8632	1.9428	2.1309	2.0054	1.9671	1.9143	0.1125
THBS2	1.3483	0.4169	1.6922	2.4006	1.8464	-0.1904	2.3912	1.6025	1.4358	3.1183	1.7653	3.6589	1.7905	1.0517
ANGPT1	1.1522	1.1726	1.6448	1.8830	1.8110	2.1614	1.5710	1.5447	1.7978	2.0573	2.1347	2.1555	1.7572	0.3536
LAMA1	1.7182	1.1431	1.4287	1.9355	1.8490	1.8434	1.6374	1.7218	1.4260	1.9461	1.9462	1.9459	1.7118	0.2597
FLT4	1.1029	1.0243	1.6636	1.8137	1.3626	1.5459	1.2442	1.2318	1.5792	2.1730	2.1232	1.9737	1.5698	0.3909

ANGPT2	1.7619	1.5045	1.6281	1.5701	2.2540	1.0733	1.3666	1.7656	1.9683	1.0562	1.0653	1.6965	1.5592	0.3730
ITGB7	0.1468	-0.0172	1.2773	6.9028	2.7097	1.7886	1.2035	1.7243	0.2312	0.3979	0.5463	0.3645	1.4396	1.9078
KIT	-0.1392	0.4861	0.6579	2.2780	2.7773	2.4263	1.3288	0.6004	0.6547	1.5405	2.4461	2.1043	1.4301	0.9665
SYK	-0.4047	1.1501	-0.3151	2.0561	0.0704	1.4037	0.8104	1.7708	2.6176	2.0959	3.0479	2.0519	1.3629	1.1296
ITGA11	1.2560	1.3294	1.2563	1.5514	1.2579	1.2581	1.2561	1.2560	1.2563	1.4782	1.3960	1.2568	1.3174	0.1029
FGF8	1.1754	1.2038	1.1113	1.2381	0.7613	0.7708	1.0419	0.7325	1.2514	1.3381	1.1697	1.0834	1.0731	0.2074
ITGB6	0.6130	0.8151	0.6135	0.6164	0.8290	0.6163	1.0430	1.0346	1.3379	1.0360	1.5141	1.3491	0.9515	0.3210
KITL	1.4558	-0.2109	1.2533	-1.0593	2.1141	-1.0797	1.7541	0.0099	1.5745	1.0249	2.0096	1.7600	0.8839	1.1623
ITGA9	-0.1061	1.0027	0.5629	1.3077	0.8084	0.8353	0.5271	0.5145	-0.0961	2.0848	1.1914	1.9065	0.8783	0.6803
GH	0.8069	0.4241	0.0184	0.7824	1.4101	0.7696	1.2436	0.5015	0.0180	1.2288	1.8797	1.4014	0.8737	0.5776
IFNAR2	0.1160	0.9537	0.2036	3.6672	0.5839	0.6227	1.1851	1.1590	0.1991	0.3630	0.5085	0.3302	0.8243	0.9664
IFNAR1	0.1104	-0.0496	0.1972	3.4889	0.5746	0.6132	1.1787	0.1165	1.2458	0.3552	1.6179	0.3226	0.8143	0.9899
LPAR3	0.4644	0.4641	0.4646	0.6418	0.4658	0.8968	0.6568	0.7313	0.4646	1.5006	1.2505	1.6515	0.8044	0.4307
IL2RG	1.1359	0.9379	0.1850	2.3569	0.5644	0.6032	0.1252	0.1039	0.1805	0.3439	0.4892	0.3112	0.6114	0.6374
IL6RA	-0.0359	-0.2008	0.0532	3.7491	0.4387	0.4780	-0.0078	-0.0296	0.0486	0.2150	0.3625	1.3028	0.5311	1.0877
TRP53	-0.0532	-0.2157	0.0346	0.4747	1.5949	0.4532	-0.0255	1.0352	0.0300	0.1939	0.3393	1.8632	0.4771	0.6736
GNG7	0.4285	0.4180	0.4348	0.4747	0.4683	0.4724	0.4304	0.4289	0.4344	0.4475	0.4608	0.4448	0.4453	0.0194
FGF22	0.4285	0.4283	0.4287	0.4296	0.4294	0.4295	0.4286	0.4285	0.4287	0.4290	0.4293	0.4289	0.4289	0.0004
COL9A1	0.0464	0.1346	0.1391	0.4905	0.6915	0.6198	0.3863	0.2891	0.2046	0.7261	0.6713	0.7264	0.4271	0.2582
ITGB3	1.1113	-1.0055	1.2303	-0.2871	1.7388	-0.3091	0.5169	-0.8269	-0.7461	1.8693	-0.4256	1.4009	0.3556	1.0698
NTRK1	0.1371	0.4428	0.3619	0.1448	0.1437	0.1444	0.1374	0.1372	0.1381	0.9245	0.4397	0.5695	0.3101	0.2493
ERBB4	0.3158	0.2295	0.2298	0.2307	0.4969	0.2306	0.2297	0.3165	0.4089	0.2301	0.3637	0.4359	0.3098	0.0962
PDGFB	0.1770	0.1443	0.1963	0.3116	0.2939	0.3052	0.1830	0.1783	0.1953	0.2344	0.2728	0.2263	0.2265	0.0568
CREB3	0.8981	-0.3915	-0.1454	1.5362	0.2276	0.2656	-0.2044	-0.2256	-0.1498	0.0112	0.1539	-0.0210	0.1629	0.5464
OSMR	0.0681	0.0629	0.0712	0.0915	0.0882	0.0903	0.0690	0.0683	0.0711	0.0776	0.0844	0.0762	0.0766	0.0098
VTN	0.0643	0.0641	0.0644	0.0652	0.0651	0.0652	0.0643	0.0643	0.0644	0.0646	0.0649	0.0646	0.0646	0.0004
FGFR2	0.0862	0.0433	0.1108	-0.0060	0.0575	0.0671	-0.0366	-0.0913	-0.0809	0.1130	0.2006	0.1859	0.0541	0.0944
MYB	-0.1865	-0.3164	-0.1154	0.2482	0.1977	0.2301	-0.1641	-0.1815	-0.1191	0.0149	0.1352	-0.0120	-0.0224	0.1879
OSM	-0.1161	-0.1165	-0.1159	-0.1143	-0.1146	-0.1144	-0.1160	-0.1161	-0.1159	-0.1154	-0.1149	-0.1155	-0.1155	0.0007
BAD	-1.2585	-0.1141	0.7350	1.2444	1.1741	-0.9670	-1.2431	0.0579	0.7298	-1.1187	0.4579	-1.1375	-0.1200	0.9845

THEM4	-0.4022	-0.5340	-0.3303	0.0357	-0.0149	0.0176	-0.3796	-0.3971	-0.3340	1.0283	-0.0778	-0.2259	-0.1345	0.4121
IL4RA	-0.3293	-0.4707	-0.2525	0.1358	0.0823	0.1167	-0.3051	-0.3239	-0.2565	-0.1125	0.0158	-0.1414	-0.1534	0.2015
PDGFD	-0.1427	-0.2440	-0.1321	-0.2427	0.1730	-0.2428	-0.0488	-0.1420	-0.2437	-0.2434	-0.2431	-0.2434	-0.1663	0.1253
CHRM1	-0.1419	-0.3298	-0.0409	-1.2772	-1.3216	1.5668	-1.6495	0.5115	-1.6078	0.1420	1.9887	0.1045	-0.1712	1.1742
GNGT2	0.8633	1.0637	-1.1612	0.8720	-0.7896	0.8450	-1.2211	-1.2426	-1.1657	-1.0039	1.8306	-1.0361	-0.1788	1.1586
TLR4	-0.7810	-0.9518	-0.6895	3.0622	-0.2992	1.1586	-0.7520	-0.7745	-0.6942	-0.5247	-0.3758	-0.5585	-0.1817	1.1591
SPP1	-0.2871	-0.2872	-0.2870	-0.2865	-0.2866	-0.2866	-0.2871	-0.2871	-0.2870	-0.2869	-0.2867	-0.2869	-0.2869	0.0002
CD19	-0.3122	-2.1706	0.9526	-1.5936	1.0030	-1.6110	0.4256	0.3970	-0.2088	-1.8231	0.1716	-0.0469	-0.4014	1.1144
IBSP	-0.5031	-0.3653	-0.5030	-0.5024	-0.5025	-0.5024	-0.4114	-0.5031	-0.5030	-0.5028	-0.5026	-0.3907	-0.4743	0.0523
GNG4	-0.5542	-0.5577	-0.5521	-0.5382	-0.5405	-0.5391	-0.5535	-0.5541	-0.1357	-0.5477	-0.5431	-0.5487	-0.5137	0.1192
GNG13	-0.6184	-0.6185	-0.6184	-0.3965	-0.6181	-0.4013	-0.6184	-0.6184	-0.4794	-0.5304	-0.6182	-0.5336	-0.5558	0.0872
FGF6	-0.6210	-0.6211	-0.6209	-0.4552	-0.3304	-0.6205	-0.6210	-0.6210	-0.6209	-0.3901	-0.6206	-0.6208	-0.5636	0.1069
AREG	-0.5477	-0.6548	-0.5754	-0.5576	-0.7427	-0.5615	-0.3791	-0.5465	-0.4528	-0.6038	-0.6563	-0.5522	-0.5692	0.0941
PGF	-0.5961	-0.5961	-0.5960	-0.5958	-0.5959	-0.5958	-0.5961	-0.5961	-0.5960	-0.5960	-0.5959	-0.5960	-0.5960	0.0001
KDR	-0.4652	-0.5896	-1.4095	-1.3321	-1.3440	-1.3364	0.9616	-0.4603	-1.4102	0.6054	-0.1426	-0.2925	-0.6013	0.8077
IL2RA	-0.7584	-0.7810	-0.7450	0.4065	-0.6774	-0.6696	-0.7543	-0.7575	-0.7457	-0.7186	-0.6921	-0.7243	-0.6348	0.3297
EGF	-0.7318	-0.7929	-0.6968	-0.5020	-0.5304	-0.5122	-0.7209	-0.7294	-0.6986	-0.6298	-0.5651	-0.6439	-0.6462	0.0980
TLR2	-0.8776	-1.0278	-0.7969	-0.3954	-0.4501	-0.4149	-0.8522	-0.8719	-0.8011	-0.6509	-0.5184	-0.6809	-0.6948	0.2096
NGFR	-0.7637	-0.7639	-0.7635	-0.7627	-0.5775	-0.7627	-0.5499	-0.7637	-0.7635	-0.6146	-0.7630	-0.7633	-0.7177	0.0838
G6PC	-0.7807	-0.7808	-0.7806	-0.7803	-0.7804	-0.7804	-0.7807	-0.7807	-0.7806	-0.6854	-0.7804	-0.6888	-0.7650	0.0364
LAMA4	-0.8970	-0.9511	-0.9511	-0.9509	-0.7809	-0.7057	-0.7977	-0.8012	-0.7443	-0.5414	-0.6758	-0.8818	-0.8066	0.1275
IGF1	-1.6945	0.3299	-1.6221	-1.2663	1.1370	-1.2834	-0.0902	-1.6893	-1.6258	0.8570	-1.3745	-1.5186	-0.8201	1.0668
FGF21	0.1598	-1.5044	-1.2332	0.8541	-0.8353	-0.7955	-1.2976	-1.3207	-1.2381	-1.0645	-0.9129	-1.0989	-0.8573	0.6866
LAMC3	-0.9023	-0.9023	-0.9022	-0.9019	-0.9020	-0.9020	-0.9022	-0.9023	-0.9022	-0.9021	-0.9020	-0.9021	-0.9021	0.0001
CHRM2	-0.9924	-0.9925	-0.8430	-0.9921	-0.9921	-0.9921	-0.9191	-0.9209	-0.9924	-0.9923	-0.9922	-0.9923	-0.9678	0.0482
EPO	-1.0478	-1.0043	-1.0477	-1.0476	-1.0476	-0.9545	-1.0477	-1.0478	-1.0477	-1.0477	-0.9653	-0.9802	-1.0238	0.0370
FASL	-1.9894	-2.0972	-1.9314	0.8959	0.1285	0.1717	-1.9711	-0.3863	-1.9344	-0.1173	-1.7315	-1.8481	-1.0676	1.1063
PCK1	-1.1942	-1.1942	-1.1942	-1.1940	-1.1941	-1.1940	-1.1408	-1.1942	-1.1942	-1.1941	-1.1941	-1.1941	-1.1897	0.0154
GNG2	-1.4760	-1.6326	-1.3925	-0.9830	-1.0382	-1.0027	-1.4496	-1.4701	-1.3968	-1.2425	-1.1074	-1.2731	-1.2887	0.2149
ITGB8	-1.9229	-2.0350	-1.8626	-1.5635	-1.6040	-1.5779	-1.9038	-1.9186	-1.8657	-0.0549	0.7982	-1.7759	-1.4406	0.8787

FGF2	-1.4835	-1.4835	-1.4835	-1.4834	-1.3992	-1.4834	-1.4835	-1.4835	-1.4835	-1.4834	-1.4834	-1.4834	-1.4764	0.0243
NTRK2	-0.5596	-2.7684	-0.4387	0.9878	-2.1694	-2.1350	-2.5791	-2.6001	-2.5252	-2.3701	-2.2368	1.0436	-1.5292	1.4092
FGF20	-1.5602	-1.5202	-1.5602	-1.5601	-1.5602	-1.5602	-1.5602	-1.5602	-1.5602	-1.5602	-1.5602	-1.4980	-1.5517	0.0204
FGF1	-1.7270	-1.7545	-1.7111	-1.6208	-1.6342	-1.6256	-1.7221	-1.7259	-1.7120	-0.5373	-1.6504	-1.6869	-1.5923	0.3353
IL6	-1.7497	-1.8528	-1.6936	-1.4093	-1.4484	-1.4233	-1.7320	-1.7457	-1.6965	-1.5911	-1.4971	-1.6122	-1.6210	0.1474
IL2RB	-1.8713	-2.0253	-1.7895	-1.3920	-1.4453	-1.4110	-1.8454	-1.8655	-1.7937	-1.6432	-1.5121	-1.6730	-1.6889	0.2092
PRKAA2	-1.7040	-1.7447	-1.9747	-1.9719	-1.9724	-1.9721	-1.9750	-1.9751	-1.9747	-1.2915	-1.9729	-1.9740	-1.8752	0.2076
CSF3R	-2.4429	-2.5979	-2.3613	-1.9714	-2.0230	-1.9898	-2.4170	-2.4371	-0.3919	-2.2165	-2.0881	-2.2459	-2.0985	0.5748
IGF2	-2.1103	-2.1113	-2.1098	-2.1060	-2.1066	-2.1062	-2.1102	-2.1103	-2.1098	-2.1086	-2.1073	-2.1089	-2.1088	0.0018
IFNB1	-2.5172	-2.5173	-2.5172	-2.5172	-2.5172	-2.5172	-2.5172	-2.5172	-2.5172	-2.5172	-2.5172	-2.5172	-2.5172	0.0000
IFNA13	-2.5826	-2.5826	-2.5826	-2.5826	-2.5826	-2.5826	-2.5826	-2.5826	-2.5826	-2.5826	-2.5826	-2.5826	-2.5826	0.0000
IL7R	-2.8376	-2.9468	-2.7798	-2.5028	-2.5395	-2.5159	-2.8193	-2.8335	-2.7828	-2.6771	-2.5858	-2.6980	-2.7099	0.1465
TGFA	-3.2389	-3.3350	-3.1882	-0.5497	-2.9786	-2.9582	-3.2228	-3.2353	-3.1908	-3.0983	-3.0187	-3.1165	-2.9276	0.7576
PRL	-2.9419	-2.9419	-2.9419	-2.9419	-2.9419	-2.9419	-2.9419	-2.9419	-2.9419	-2.9419	-2.9419	-2.9419	-2.9419	0.0000
FGF16	-3.0169	-3.0169	-3.0169	-3.0168	-3.0168	-3.0168	-3.0169	-3.0169	-3.0169	-3.0169	-3.0168	-3.0169	-3.0169	0.0000
FGF10	-4.0907	-4.1551	-4.0567	-3.7961	-3.8324	-3.8090	-1.7320	-4.0882	-4.0584	-3.9667	-3.8779	-3.9869	-3.7875	0.6586
FGF5	-3.8711	-3.8711	-3.8711	-3.8696	-3.8698	-3.8697	-3.8711	-3.8711	-3.8711	-3.8707	-3.8701	-3.8708	-3.8706	0.0006
NTF3	-3.9397	-3.9397	-3.9395	-3.9379	-3.9381	-3.9380	-3.9097	-3.9397	-3.9395	-3.9389	-3.9384	-3.9390	-3.9365	0.0085
FGF15	-4.3022	-4.3060	-4.3000	-4.2891	-4.2906	-4.2896	-4.3015	-4.3020	-4.3001	-4.2960	-4.2924	-4.2968	-4.2972	0.0056
FGF3	-4.3405	-4.3408	-4.3404	-4.3397	-4.3398	-4.3397	-4.3405	-4.3405	-4.3404	-4.3401	-4.3399	-4.3402	-4.3402	0.0004
IFNA15	-4.3699	-4.3702	-4.3697	-4.3689	-4.3690	-4.3689	-4.3698	-4.3699	-4.3697	-4.3694	-4.3691	-4.3695	-4.3695	0.0004
IL2	-4.5697	-4.5698	-4.5696	-4.5693	-4.5693	-4.5693	-4.5697	-4.5697	-4.5696	-4.5695	-4.5694	-4.5695	-4.5695	0.0002
RPS6e	#NV	#NV	#NV	#NV	#NV	#NV	#NV	#NV	#NV	#NV	#NV	#NV	#NV	#NV
PPP2C	#NV	#NV	#NV	#NV	#NV	#NV	#NV	#NV	#NV	#NV	#NV	#NV	#NV	#NV
INS	#NV	#NV	#NV	#NV	#NV	#NV	#NV	#NV	#NV	#NV	#NV	#NV	#NV	#NV
GNG1	#NV	#NV	#NV	#NV	#NV	#NV	#NV	#NV	#NV	#NV	#NV	#NV	#NV	#NV
IL3	#NV	#NV	#NV	#NV	#NV	#NV	#NV	#NV	#NV	#NV	#NV	#NV	#NV	#NV
ILR3A	#NV	#NV	#NV	#NV	#NV	#NV	#NV	#NV	#NV	#NV	#NV	#NV	#NV	#NV
IL4R	#NV	#NV	#NV	#NV	#NV	#NV	#NV	#NV	#NV	#NV	#NV	#NV	#NV	#NV
IL4	#NV	#NV	#NV	#NV	#NV	#NV	#NV	#NV	#NV	#NV	#NV	#NV	#NV	#NV

AIP3	#NV	#NV	#NV	#NV	#NV	#NV	#NV	#NV	#NV	#NV	#NV	#NV	#NV	#NV
LAMB4	#NV	#NV	#NV	#NV	#NV	#NV	#NV	#NV	#NV	#NV	#NV	#NV	#NV	#NV
IGH	#NV	#NV	#NV	#NV	#NV	#NV	#NV	#NV	#NV	#NV	#NV	#NV	#NV	#NV
TCL1A	#NV	#NV	#NV	#NV	#NV	#NV	#NV	#NV	#NV	#NV	#NV	#NV	#NV	#NV
IFNA1	#NV	#NV	#NV	#NV	#NV	#NV	#NV	#NV	#NV	#NV	#NV	#NV	#NV	#NV
IFNA2	#NV	#NV	#NV	#NV	#NV	#NV	#NV	#NV	#NV	#NV	#NV	#NV	#NV	#NV
IFNA3	#NV	#NV	#NV	#NV	#NV	#NV	#NV	#NV	#NV	#NV	#NV	#NV	#NV	#NV
IFNA4	#NV	#NV	#NV	#NV	#NV	#NV	#NV	#NV	#NV	#NV	#NV	#NV	#NV	#NV
IFNA5	#NV	#NV	#NV	#NV	#NV	#NV	#NV	#NV	#NV	#NV	#NV	#NV	#NV	#NV
IFNA6	#NV	#NV	#NV	#NV	#NV	#NV	#NV	#NV	#NV	#NV	#NV	#NV	#NV	#NV
IFNA7	#NV	#NV	#NV	#NV	#NV	#NV	#NV	#NV	#NV	#NV	#NV	#NV	#NV	#NV
IFNA9	#NV	#NV	#NV	#NV	#NV	#NV	#NV	#NV	#NV	#NV	#NV	#NV	#NV	#NV
IFNA10	#NV	#NV	#NV	#NV	#NV	#NV	#NV	#NV	#NV	#NV	#NV	#NV	#NV	#NV
IFNA11	#NV	#NV	#NV	#NV	#NV	#NV	#NV	#NV	#NV	#NV	#NV	#NV	#NV	#NV
IFNA12	#NV	#NV	#NV	#NV	#NV	#NV	#NV	#NV	#NV	#NV	#NV	#NV	#NV	#NV
IFNA14	#NV	#NV	#NV	#NV	#NV	#NV	#NV	#NV	#NV	#NV	#NV	#NV	#NV	#NV
IFNA16	#NV	#NV	#NV	#NV	#NV	#NV	#NV	#NV	#NV	#NV	#NV	#NV	#NV	#NV
FGF4	#NV	#NV	#NV	#NV	#NV	#NV	#NV	#NV	#NV	#NV	#NV	#NV	#NV	#NV
FGF19	#NV	#NV	#NV	#NV	#NV	#NV	#NV	#NV	#NV	#NV	#NV	#NV	#NV	#NV
IL3RA	#NV	#NV	#NV	#NV	#NV	#NV	#NV	#NV	#NV	#NV	#NV	#NV	#NV	#NV
KLK1B4	#NV	#NV	#NV	#NV	#NV	#NV	#NV	#NV	#NV	#NV	#NV	#NV	#NV	#NV

Table S2 Relative expression values of genes related to the MAPK signaling pathway

MAPK Signaling	Replicate 1	Replicate 2	Replicate 3	Replicate 4	Replicate 5	Replicate 6	Replicate 7	Replicate 8	Replicate 9	Replicate 10	Replicate 11	Replicate 12	Mean	SD	Comments
GAPDH	13.5720	13.8087	13.5775	12.9286	12.6895	12.9789	13.4230	13.6322	13.6013	13.3205	13.3560	13.2579	13.3455	0.3331	Positive Reference Gene
EPO	-1.0478	-1.0043	-1.0477	-1.0476	-1.0476	-0.9545	-1.0477	-1.0478	-1.0477	-1.0477	-0.9653	-0.9802	-1.0238	0.0370	Negative Reference Gene
FLNA	14.6223	14.3730	14.7033	13.6465	13.9358	13.7013	14.7123	14.5462	14.6619	13.6140	13.6022	14.0542	14.1811	0.4671	
MYC	12.9329	12.9820	12.9262	12.9637	12.8473	12.9155	13.3571	13.3971	13.3457	12.8349	12.8262	12.6471	12.9980	0.2391	
ATF4	12.5945	12.9625	12.4914	12.9263	12.6672	12.9056	12.5733	12.6845	12.7573	12.5026	12.5696	12.3154	12.6625	0.1965	
NFKB2	11.4506	11.4960	11.4219	12.0260	12.0092	12.0393	11.4766	11.3834	11.5263	12.2808	12.2804	12.0406	11.7859	0.3542	
MAP4K4	11.9266	11.8576	11.9021	11.8062	11.8948	11.8367	11.5736	11.4977	11.5304	11.4819	11.4879	11.6219	11.7014	0.1832	
RAC1	11.4810	11.5909	11.4031	11.7228	11.7026	11.7549	11.5017	11.5636	11.5211	11.2911	11.3533	11.0922	11.4982	0.1930	
MAP4K3	10.9248	10.7711	10.9341	11.8378	11.8501	11.8805	11.1983	11.0721	11.1983	11.3584	11.3474	10.6981	11.2559	0.4157	
MAP3K1	10.7289	10.5733	10.7962	11.7537	11.8403	11.5933	10.9151	10.8141	10.9113	11.1390	11.1006	10.8531	11.0849	0.4197	
NF1	10.7614	10.6137	10.8266	11.1486	11.3715	10.9602	11.2281	11.0184	11.1824	11.3911	11.3332	11.0163	11.0710	0.2500	
RASA2	10.9057	10.7899	10.9869	10.9692	11.2571	10.9078	11.3378	11.2145	11.3499	11.0675	10.9331	10.6511	11.0309	0.2196	
MAPK8IP3	10.1428	10.1673	10.1838	11.8744	12.0172	11.8248	10.7308	10.5895	10.8765	11.4922	11.5217	10.8682	11.0241	0.6977	
MAP3K12	10.2318	10.2517	10.2354	11.9363	11.7942	11.8346	10.7689	10.6921	10.8398	10.8001	10.7945	10.2312	10.8675	0.6449	
TGFBR1	11.2601	11.0518	11.4006	10.5705	10.6087	10.6236	10.6757	10.5101	10.6835	10.2762	10.3056	10.9325	10.7416	0.3522	
DDIT3	10.0852	10.2607	10.0445	11.6879	11.5064	11.6041	9.7388	9.8528	9.7443	11.2254	11.2796	11.1243	10.6795	0.7855	
MAPK14	10.3489	10.2936	10.3561	11.1923	11.2290	11.1084	10.5954	10.5394	10.5756	10.6955	10.7439	10.4264	10.6754	0.3327	
TGFB1	10.5564	10.6520	10.5583	10.6362	10.4659	10.6830	10.7207	10.7676	10.7804	10.5501	10.6078	10.6985	10.6398	0.0958	
STK4	10.3198	10.1865	10.2559	10.5387	10.5216	10.5354	10.3466	10.3184	10.2454	11.2803	11.2596	11.1646	10.5811	0.4116	
CDC42	10.7727	10.8494	10.7354	10.4388	10.3329	10.4526	10.6853	10.7457	10.6397	10.2710	10.3346	10.3976	10.5547	0.2033	
CACNA2D1	10.7080	10.4222	10.7647	10.1435	10.3569	10.3253	10.7095	10.5023	10.6588	10.6234	10.5669	10.8325	10.5512	0.2057	
IRAK1	10.3188	10.2393	10.3591	10.9731	11.0974	10.8954	10.4993	10.3232	10.5103	10.5419	10.4870	10.1428	10.5323	0.3024	
TAB2	10.4280	10.3071	10.3547	10.7058	10.7557	10.6595	10.4362	10.2607	10.2436	10.7410	10.6673	10.5581	10.5098	0.1936	
GNG12	10.4733	10.3992	10.3948	10.4484	10.6226	10.3424	10.5108	10.2932	10.2469	10.6665	10.5469	10.7365	10.4735	0.1504	
CDC25B	10.4021	10.4341	10.4384	10.0178	10.0065	9.9710	11.1495	11.0840	11.1813	10.2777	10.2129	10.2345	10.4508	0.4446	

MAPKAPK2	10.3774	10.4279	10.3441	10.7053	10.6120	10.7226	10.3499	10.2632	10.2861	10.3019	10.3917	10.4135	10.4330	0.1589
PAK2	10.8037	10.8191	10.7984	9.7236	9.8825	9.8230	10.5040	10.4781	10.4335	10.3719	10.4147	10.6758	10.3940	0.3873
ECSIT	10.1581	10.2444	10.1265	10.6303	10.5974	10.5351	10.6034	10.5844	10.4886	10.3682	10.3096	10.0652	10.3926	0.2071
TRAF2	9.9029	9.8104	9.8047	10.5378	10.5474	10.5411	10.0350	9.9398	10.0506	11.2022	11.2103	10.6092	10.3493	0.5036
MKNK1	9.9169	9.8522	9.9427	11.0009	10.9684	10.9798	9.9860	9.8532	10.0980	10.6145	10.5883	10.1474	10.3290	0.4680
VEGFA	9.7805	9.7162	9.8433	11.4271	11.4914	11.2323	9.6843	9.5422	9.7503	10.3565	10.3363	10.0384	10.2666	0.7194
TGFBR2	10.5948	10.3838	10.6294	9.4737	9.7400	9.9576	10.5383	10.5731	10.5382	9.7303	9.7307	10.7174	10.2173	0.4518
MAPKAPK5	9.8651	9.8562	9.8871	10.5880	10.6521	10.4448	10.0683	10.0742	10.1907	10.2497	10.2221	9.8417	10.1617	0.2845
MET	10.2139	9.8769	10.3383	9.4042	9.5582	9.6294	10.7528	10.6375	10.6722	9.9925	9.9368	10.8485	10.1551	0.4976
RAF1	10.0856	10.0764	10.0724	10.1959	10.3417	10.2593	10.3624	10.1845	10.1787	10.0367	9.9888	10.0595	10.1535	0.1208
IKBKB	9.5529	9.3989	9.5995	10.8800	10.7881	10.7766	9.8436	9.8612	9.9876	10.5475	10.5252	10.0178	10.1482	0.5288
INSR	10.2067	9.8869	10.2950	9.9354	10.0969	9.9412	9.7433	9.6800	9.7487	10.1873	10.2082	10.9359	10.0721	0.3415
EPHA2	10.5958	10.3902	10.6848	8.7759	8.7455	9.0197	10.3555	10.2023	10.3339	10.0302	10.0438	11.0603	10.0198	0.7634
TAB1	9.7723	9.6933	9.7772	10.5735	10.5421	10.4612	10.2021	10.1147	10.2799	9.6887	9.6971	9.2974	10.0083	0.4095
SOS1	9.6752	9.6241	9.6611	10.2777	10.2333	10.2617	9.8577	9.7789	9.7355	10.1170	10.1319	10.0824	9.9530	0.2544
MAP3K7	9.7494	9.7120	9.7082	10.1514	10.0787	10.2006	9.9609	9.8364	9.8055	10.0394	10.0849	9.8458	9.9311	0.1763
MAP2K3	9.9593	9.9888	9.9151	9.5860	9.5667	9.5562	10.0947	9.9875	9.9723	10.0598	10.1190	10.0890	9.9079	0.2129
MYD88	9.6416	9.6735	9.6430	10.1874	10.2185	10.1543	9.6147	9.5939	9.6678	10.1526	10.0687	10.0940	9.8925	0.2682
RPS6KA4	9.6666	9.6578	9.7024	10.0259	9.9189	10.0289	10.1267	10.0596	9.9698	9.7850	9.7959	9.8918	9.8858	0.1619
AKT1	9.8190	9.7996	9.7243	10.0171	9.9920	10.0747	10.0875	9.8846	9.8976	9.7995	9.7503	9.7493	9.8830	0.1305
GADD45A	9.8741	10.0123	9.7187	10.1249	9.9129	10.0519	9.1781	9.2208	9.1638	10.2680	10.2712	10.5239	9.8600	0.4560
RAP1B	9.9172	9.8073	9.7480	10.0297	9.9214	10.0482	9.8054	9.7660	9.6010	9.8309	9.8272	10.0123	9.8596	0.1316
PPP5C	9.8370	9.8655	9.7583	9.7827	9.8125	9.6397	9.9077	9.8562	9.8563	9.7596	9.9155	9.8056	9.8164	0.0758
NFKB1	9.7781	9.7069	9.8188	9.4854	9.6087	9.6568	9.7400	9.6161	9.5476	10.0923	10.0524	10.3081	9.7843	0.2470
GRB2	9.8187	9.8374	9.8203	9.8006	9.6244	9.7788	9.8335	9.8058	9.7379	9.5354	9.6914	9.7674	9.7543	0.0936
MAP3K4	9.5139	9.4349	9.5182	9.7000	9.6872	9.7741	9.8194	9.7813	9.6903	9.8762	9.7519	10.2225	9.7308	0.2045
ATF2	9.4606	9.5301	9.5085	10.0978	10.0912	10.0961	9.6065	9.4562	9.5388	9.7094	9.6488	9.5017	9.6871	0.2568
HSPB1	8.9793	9.3684	8.8754	10.0494	9.8968	10.0969	9.3921	9.7360	9.5383	9.9692	9.9088	9.9243	9.6446	0.4134
MAPK1	10.0027	9.9077	9.9221	9.5429	9.5066	9.4020	9.8633	9.7680	9.6867	9.2965	9.3009	9.3329	9.6277	0.2630
RELA	9.6768	9.6231	9.5984	9.1628	9.3076	9.3082	9.6839	9.5520	9.4918	9.9192	9.8013	10.2676	9.6161	0.2976

PPM1A	9.7892	9.6316	9.7090	9.4710	9.6172	9.6124	9.7086	9.5422	9.5209	9.4313	9.3467	9.7977	9.5981	0.1410
RELB	9.1654	9.1799	9.1256	9.6368	9.6116	9.6608	9.0890	8.9621	9.0627	10.5664	10.4937	10.4649	9.5849	0.6053
CACNA1C	9.2968	9.2864	9.4416	9.8015	9.8428	9.7841	9.7208	9.5777	9.7368	9.6175	9.6079	9.2685	9.5819	0.2108
MAX	8.7042	8.7729	8.7278	10.5322	10.5687	10.4006	9.1361	9.1777	9.0220	10.0481	10.0898	9.6859	9.5722	0.7300
EREG	10.1187	9.9371	10.1513	9.1945	8.8706	9.0163	9.2268	9.1994	9.4183	9.7434	9.7694	9.8909	9.5447	0.4425
MRAS	8.8774	8.8499	8.7543	9.9834	9.9178	10.0064	9.1383	9.0664	8.9846	10.1611	10.0805	9.9052	9.4771	0.5679
PPP3R1	9.7931	9.6074	9.6445	9.2137	9.2043	9.1748	9.6686	9.4613	9.3892	9.2361	9.1455	9.5699	9.4257	0.2276
MAP2K2	9.4592	9.5844	9.4184	9.2923	9.4514	9.3256	9.5387	9.4778	9.4350	9.2165	9.1145	9.3919	9.3921	0.1344
MAP3K6	9.4684	9.4893	9.4672	9.8260	9.7044	9.7126	9.2964	9.1349	9.4010	9.2202	9.1889	8.7643	9.3895	0.2938
PAK1	8.7582	8.6824	8.6718	9.8553	9.7736	9.8292	9.0700	8.9782	8.9040	9.8715	9.7577	9.5020	9.3045	0.5020
PRKACA	9.2398	9.3129	9.1405	9.3065	9.0757	9.1671	9.3353	9.5019	9.2369	9.3036	9.2951	9.5150	9.2859	0.1304
DUSP6	9.8399	9.9182	9.9076	8.6742	8.6594	8.6269	9.7686	9.7656	9.7741	8.8352	8.7936	8.8214	9.2820	0.5766
RASA1	9.4662	9.3111	9.4258	9.2266	9.4703	9.1303	9.3681	9.1837	9.2466	9.1498	9.1424	9.1772	9.2748	0.1289
RAPGEF2	9.2518	9.0596	9.1588	9.3913	9.3328	9.4130	9.3763	9.1927	9.1505	9.2352	9.2494	9.1879	9.2499	0.1092
MAP3K2	8.9694	8.6742	9.0996	8.5858	9.2398	8.3965	9.6420	9.5939	9.6440	9.6527	9.5644	9.7729	9.2363	0.4826
FGFR1	9.1670	8.9461	9.2649	9.2462	9.2356	9.3703	9.2159	9.1531	9.1281	9.1745	9.2275	9.7014	9.2359	0.1777
NFATC3	9.1470	9.0580	9.2363	8.7880	9.0975	8.9183	9.1304	9.1201	9.2130	9.1423	9.1947	9.3777	9.1186	0.1510
PLA2G4A	9.3219	9.1586	9.2378	8.7891	8.7908	8.8917	9.2799	9.2749	9.2785	8.9525	8.8207	9.2940	9.0909	0.2213
SRF	9.1568	9.1807	9.1050	8.6418	8.8023	8.7578	9.2244	8.9891	8.9265	9.1262	9.3264	9.4470	9.0570	0.2400
CASP3	9.0639	9.1779	9.1428	8.9040	8.8985	9.0142	9.1455	9.2902	8.9652	8.9743	8.9602	9.0630	9.0500	0.1201
MAPK7	8.6759	8.7414	8.7406	9.3828	9.4298	9.4244	8.9860	8.9289	9.0774	9.2607	9.1259	8.8243	9.0498	0.2774
MAPKAPK3	8.8456	8.8247	8.8194	9.0314	9.1321	8.9947	8.8234	8.7122	8.6238	9.4111	9.3115	9.5819	9.0093	0.2961
CACNB3	8.3429	8.2774	8.3782	9.4933	9.5616	9.6263	9.1226	9.0194	9.0217	9.1736	9.1673	8.8529	9.0031	0.4658
ARAF	9.0942	8.9330	9.1606	8.9126	8.9479	8.9725	9.1350	9.1366	9.1780	8.6556	8.6039	8.9620	8.9743	0.1878
EGFR	7.8056	7.6686	7.8223	11.1868	9.8082	10.9622	8.2306	7.9687	8.2839	9.6179	9.4639	8.7251	8.9620	1.2320
MAP3K11	8.9066	8.9885	8.9987	8.9087	8.9609	9.0254	8.9194	8.7274	8.8624	8.9375	8.8458	9.2037	8.9404	0.1151
PDGFRA	8.7241	8.4761	8.7502	9.0801	9.0547	9.1926	9.0553	8.9531	9.0290	8.5550	8.5331	9.3950	8.8999	0.2888
DUSP3	8.6762	8.6058	8.5612	9.0355	9.1066	8.9774	8.8861	8.9094	8.8076	9.0952	8.9872	9.1270	8.8979	0.1964
PPM1B	9.1956	9.1900	9.1666	8.4252	8.4718	8.4289	8.9764	9.0312	8.9495	8.9288	8.9260	8.9719	8.8885	0.2867
IGF1R	8.9767	8.7371	9.1688	8.2517	8.6963	8.1217	9.4528	9.3262	9.3846	8.7205	8.6269	9.0556	8.8766	0.4284

NLK	8.6245	8.4778	8.6517	8.9485	8.9898	9.0536	8.8471	8.5844	8.7370	9.2009	9.1927	8.7163	8.8354	0.2409
MAP2K5	8.8445	8.8699	8.8456	9.1785	9.1956	9.0765	8.7408	8.6238	8.6935	8.6867	8.6367	8.5085	8.8251	0.2232
ERBB2	8.7272	8.4591	8.7276	8.9371	9.0450	9.1235	8.9295	8.6419	8.8685	8.5930	8.5841	8.9226	8.7966	0.2047
MAP3K14	8.3734	8.3729	8.2966	9.0614	9.2315	9.0323	9.0210	8.8372	8.8690	8.8314	8.8389	8.6621	8.7856	0.3016
IRAK4	8.2349	8.1947	8.3073	9.1613	9.2700	9.0160	8.6005	8.5444	8.6587	8.9789	8.9287	8.7746	8.7225	0.3604
LAMTOR3	8.4684	8.3990	8.4631	9.1855	9.1456	9.1806	8.3155	8.0876	8.2616	9.0547	8.8411	8.7726	8.6813	0.3963
MAP2K7	8.6034	8.3865	8.5279	9.0532	9.4034	8.9413	8.6934	8.4887	8.8313	8.3511	8.4549	8.2196	8.6629	0.3402
CACNA1G	7.9172	7.8482	7.8994	9.2717	9.4107	9.2113	8.5705	8.5237	8.5452	8.8566	8.7996	8.9943	8.6540	0.5427
ELK1	8.5737	8.4378	8.4517	9.0924	8.9244	8.8852	8.5729	8.1772	8.4063	8.5688	8.5306	8.6562	8.6064	0.2529
GNA12	9.0864	8.8548	8.9923	7.5771	8.0342	8.0163	9.1852	8.7920	8.5588	8.3782	8.3103	9.2544	8.5867	0.5311
NRAS	8.4296	8.3021	8.5704	8.6368	8.9355	8.4326	8.8141	8.5569	8.6609	8.5084	8.5073	8.5541	8.5757	0.1716
MAP3K3	8.3308	8.1705	8.3367	8.9484	9.0948	8.8574	8.6071	8.4996	8.5029	8.4587	8.5982	8.4643	8.5724	0.2710
PDGFRB	7.9081	7.6439	7.9983	8.4990	8.4875	8.5429	8.7159	8.7071	8.7307	8.8450	8.9618	9.6879	8.5607	0.5376
PRKCA	8.7510	8.5813	8.7480	8.1014	8.6334	8.0322	8.8775	8.8874	8.8351	8.2237	8.1733	8.6475	8.5410	0.3185
TRAF6	8.2276	7.9544	8.1199	8.7414	8.8724	8.6595	8.2928	8.2155	8.2953	8.8552	8.7030	8.8323	8.4808	0.3272
STK3	8.4509	8.5801	8.4393	8.0956	7.8871	8.0303	8.3554	8.4796	8.3208	8.7696	8.6760	8.9777	8.4219	0.3140
RRAS2	8.5811	8.6840	8.5556	8.2535	8.1390	8.0813	8.3154	8.4562	8.2223	8.2541	8.2262	8.3210	8.3408	0.1874
VEGFB	8.1480	8.2159	8.1707	8.5631	8.2418	8.6062	8.1490	8.4060	8.1513	8.5438	8.4290	8.4300	8.3379	0.1771
MAPK8	8.2615	8.1553	8.2422	8.4437	8.7061	8.2845	8.3905	8.4620	8.4446	8.1673	8.1158	8.0851	8.3132	0.1816
RRAS	8.1006	8.2201	8.0392	8.0818	7.9325	8.1146	8.1691	8.4552	8.3138	8.6474	8.8364	8.7519	8.3052	0.2992
MAP3K5	8.4060	8.3153	8.3698	8.0526	8.3249	8.0904	8.2637	8.3330	8.2527	8.2208	8.2177	8.4362	8.2736	0.1168
JUN	8.2986	8.4857	8.2705	8.2473	8.0772	8.2311	8.0770	8.1000	8.0416	8.4732	8.3940	8.4394	8.2613	0.1622
BRAF	8.3482	8.0927	8.4053	7.7265	8.1132	7.5837	8.3716	8.4177	8.2294	8.0057	8.1245	8.3650	8.1486	0.2703
ELK4	8.1673	7.9590	8.2655	7.9725	8.2283	7.5985	8.3067	8.2236	8.3188	7.9953	8.0482	8.3064	8.1159	0.2122
CSF1	7.6546	7.4047	7.7612	7.4425	7.5966	7.4906	8.2458	8.2866	8.1387	8.8076	8.8536	9.5481	8.1026	0.6794
CRK	8.2415	8.2447	8.1111	7.9653	8.0453	7.7432	8.1959	8.1208	8.0657	8.0399	7.8729	8.3325	8.0816	0.1666
NFATC1	8.0086	7.7701	7.9674	7.9110	8.3318	8.0869	8.1863	8.0990	8.0145	8.2512	8.0020	8.3201	8.0791	0.1691
JUND	7.6976	8.2413	7.4365	8.2049	7.8377	7.9931	7.5869	8.1177	7.5398	8.3572	8.0775	8.3789	7.9558	0.3283
RAP1A	8.0824	8.2335	7.9169	7.5834	7.4398	7.5450	8.0776	8.1364	7.7978	7.8848	7.9845	8.1375	7.9016	0.2602
VEGFC	8.3031	8.3215	8.4201	7.5995	7.6946	7.5540	7.7655	7.8188	7.8107	7.5469	7.6635	7.9141	7.8677	0.3110

STMN1	8.4030	8.5658	8.2600	6.7720	6.3151	6.9794	8.7450	8.8339	8.5294	7.2889	7.4261	7.8704	7.8324	0.8519
MAP4K2	7.5174	7.5339	7.3923	8.2555	8.3374	8.1191	7.3832	7.5170	7.6225	8.0356	7.9966	7.5731	7.7736	0.3487
ZAK	7.8704	7.9318	7.9135	7.6062	7.9201	7.6394	8.0614	7.8858	7.8797	7.5169	7.4287	7.5081	7.7635	0.2094
PDGFC	8.1474	8.0114	8.2657	7.0321	6.8760	7.2214	8.2479	8.3075	8.2457	7.3824	7.2963	7.7752	7.7341	0.5388
MEF2C	7.6385	7.5256	7.6387	7.7660	7.5010	7.2851	7.8070	7.8525	7.7943	7.7273	7.4849	7.7913	7.6510	0.1717
HRAS	7.4140	7.5140	7.3319	7.7922	7.5789	8.0662	7.1767	7.4505	7.4151	7.2996	7.4219	7.4002	7.4884	0.2368
FLT3L	6.4100	6.5775	6.5084	8.1535	8.2579	8.2127	7.1124	6.8882	7.2605	7.8517	8.0281	7.7047	7.4138	0.7051
IKBKG	7.2934	6.9957	7.3384	7.1022	7.5674	7.0720	7.6337	7.2355	7.4488	7.5654	7.5270	7.7636	7.3786	0.2444
KRAS	7.4382	7.4450	7.7496	7.1054	7.4338	7.0881	7.5359	7.6554	7.7291	7.1562	6.9418	7.1529	7.3693	0.2734
TRADD	6.9863	7.2932	7.0100	7.3623	7.5571	7.3160	7.2298	7.2276	7.0224	7.5472	7.4825	7.5001	7.2945	0.2076
EFNA1	7.0577	6.8238	7.0950	7.7223	7.6476	7.9223	6.9110	7.0473	6.9742	7.2594	7.3066	7.3331	7.2584	0.3461
EFNA3	6.7881	6.9772	6.8918	7.7464	7.6904	7.7768	6.9795	7.1610	7.2287	7.1090	7.1708	7.1839	7.2253	0.3356
DUSP1	6.5844	6.8814	6.7458	7.7172	7.8014	7.6734	7.2293	7.4977	7.2398	7.2486	7.0367	6.9615	7.2181	0.3946
MAPK8IP1	6.5113	6.2104	6.3021	7.8438	7.7190	7.6397	6.7722	6.6569	6.5432	7.6331	7.3815	7.9917	7.1004	0.6588
MAP2K4	7.2466	7.0914	6.9623	7.5096	7.1454	7.1672	7.1562	6.9129	7.0023	7.1432	6.7653	7.0626	7.0971	0.1853
DUSP10	7.0206	7.1405	6.9973	6.9141	6.9631	7.1210	6.8932	7.1194	6.9162	7.0467	6.9693	6.9266	7.0023	0.0877
ARRB1	6.6826	6.7246	7.1002	6.4460	7.0288	6.2641	7.7456	7.4558	7.5994	6.6791	6.6180	7.2557	6.9667	0.4729
PDGFA	6.6595	6.6203	6.6195	7.3033	7.2371	7.1159	6.7536	6.8338	6.9157	7.2603	7.1255	7.1333	6.9648	0.2608
RPS6KA5	6.5701	6.5074	6.7158	7.3432	7.5202	7.0189	7.2133	7.2639	7.1415	6.7923	6.7964	6.5987	6.9568	0.3378
IL1RAP	8.1864	7.8058	8.1789	5.1306	5.7663	5.6241	7.5347	7.0904	7.4289	6.5134	6.1098	7.1853	6.8796	1.0324
NR4A1	6.1747	6.4518	6.3408	7.1425	7.1376	7.0797	6.6813	6.6176	6.6949	7.3446	7.2657	7.1283	6.8383	0.3925
TAOK1	6.6042	6.3554	6.5722	6.3988	6.9816	6.4124	6.7589	6.4313	6.7552	6.8576	6.7818	6.6217	6.6276	0.2024
MAPT	5.9608	6.1216	6.0284	7.0647	7.1461	7.0308	6.2029	6.1466	6.0059	7.2853	7.0702	6.8884	6.5793	0.5350
BDNF	7.0519	6.7964	7.0678	6.5326	6.0925	6.4568	6.6489	6.7931	6.4455	6.0644	6.2084	6.1881	6.5289	0.3512
EFNA4	6.0418	5.9046	5.9291	7.0521	6.8172	6.6720	6.2893	6.3558	6.4465	6.4357	6.5616	6.3323	6.4032	0.3460
CACNG4	5.4579	5.3298	5.1810	7.1425	7.9909	6.8065	5.6849	5.6597	5.8175	7.5097	7.5710	6.6233	6.3979	0.9917
HGF	6.9725	6.7967	6.8641	6.0684	5.8991	6.1470	6.7261	6.3519	6.5987	5.8745	5.3533	6.7558	6.3673	0.5008
TGFB3	6.0037	6.0660	6.1046	6.8018	6.7685	6.6344	6.0831	6.1494	6.1400	6.1641	6.0701	6.7359	6.3101	0.3190
CACNB4	5.6185	5.1016	5.6506	6.4069	6.4921	5.7844	5.8291	6.0909	6.1039	7.3998	7.4168	7.1445	6.2533	0.7437
RAC3	5.2420	5.2232	5.4698	6.7212	7.2081	6.8744	5.8443	5.6163	6.2306	6.3691	6.4715	5.9845	6.1046	0.6506

FOS	5.9917	5.5838	5.8406	5.3521	4.8297	4.8787	5.7263	5.4642	5.3506	6.0333	5.9408	5.8760	5.5723	0.4106
FGFR3	5.2883	5.1666	5.1787	5.9480	5.8127	6.0614	5.5358	5.3093	5.1882	5.7754	5.6403	5.7087	5.5511	0.3184
ANGPT4	4.9014	4.8998	4.9744	4.8433	4.4968	4.5045	6.0776	6.0685	6.1136	5.9911	5.9444	6.8120	5.4690	0.7749
FGF18	5.4785	5.7564	5.4444	5.5463	5.5614	5.6129	5.2693	5.2671	5.1824	5.2311	5.3472	5.7394	5.4530	0.1961
CACNA1F	4.6869	4.6544	4.8950	5.6136	5.8551	4.8339	5.0814	4.9457	5.2906	5.5346	5.7081	5.4640	5.2136	0.4170
PTPRR	3.9748	3.8738	4.1521	5.9427	6.0520	5.7890	4.3602	4.2101	4.1455	6.6084	6.3374	6.7361	5.1818	1.1446
FGF9	5.1356	5.1425	5.1356	5.1524	5.1512	5.1520	5.1446	5.1356	5.1548	5.1592	5.2472	5.1583	5.1558	0.0301
EFNA5	5.5005	5.0250	5.5140	4.5666	4.7080	4.3203	5.5543	5.2932	5.1074	5.0955	4.5173	5.8556	5.0881	0.4802
TEK	3.9995	4.2114	3.9940	6.6351	6.5289	6.0084	4.3390	4.1966	4.5574	5.4896	5.4365	4.7504	5.0122	0.9738
CACNA1A	4.5906	4.3869	4.7765	5.0727	5.2534	5.1379	5.0720	5.0085	4.7951	5.3371	5.2559	5.1188	4.9838	0.2891
CACNA1D	4.0310	3.8948	4.3555	4.6093	5.0724	4.5147	5.1884	4.6567	4.6687	6.4315	6.2246	6.0743	4.9768	0.8490
CACNG2	4.6613	4.6612	4.6614	4.6619	4.6619	4.6619	4.6614	4.6614	4.6614	4.6616	4.6618	4.6615	4.6616	0.0002
VEGFD	3.9474	4.0894	3.9265	5.1260	4.5331	4.7595	4.4150	4.2028	5.0016	5.3319	5.1375	4.9825	4.6211	0.5014
MAP3K13	4.5258	4.3395	4.8107	4.8928	5.0786	3.7672	4.5644	4.3471	4.5034	4.6784	4.5167	4.6839	4.5590	0.3304
FLT1	3.9782	3.6107	3.8575	4.1140	4.0968	4.0784	4.0004	3.7485	3.9565	4.1514	4.2171	6.7571	4.2139	0.8196
FGFR4	3.6743	3.5239	3.7393	5.2257	4.9507	4.8286	3.3600	3.2425	3.3720	4.7022	4.8432	4.6625	4.1771	0.7470
FGF23	4.0844	4.0843	4.0845	4.0851	4.0850	4.0851	4.0845	4.0844	4.0845	4.0847	4.0849	4.0847	4.0847	0.0003
RASGRP3	3.2496	2.9353	3.3651	4.9134	4.8750	4.4629	3.6195	3.5906	3.6189	4.2337	4.2462	4.3950	3.9588	0.6483
TNFRSF1A	4.0666	3.7156	4.0567	3.8846	4.1511	3.8569	4.2754	3.9460	3.9047	3.5238	3.4116	4.2949	3.9240	0.2736
MECOM	3.4604	3.5856	3.8213	4.6507	5.0730	4.0117	3.7899	3.0824	3.2238	4.0849	3.9224	4.2388	3.9121	0.5669
MAP2K1	3.9547	3.9289	4.1485	4.1052	4.2472	3.4520	4.1970	3.9633	3.9114	3.6063	3.6553	3.6886	3.9049	0.2544
CACNA2D2	3.4516	3.5579	3.6991	4.3366	4.4045	4.1081	3.7394	3.2586	3.8391	3.9046	4.1961	3.7655	3.8551	0.3528
FGF7	4.1441	3.9250	4.3831	3.3315	3.5170	3.3995	3.6470	3.6958	3.8711	4.1620	3.7446	3.5239	3.7787	0.3273
CACNA2D3	3.4680	3.4133	3.6572	3.2571	3.6086	3.0541	3.8645	3.7822	3.8331	3.9478	3.8279	3.7884	3.6252	0.2746
MAP2K6	2.1790	2.7186	2.7117	3.8852	3.8099	3.3248	3.6086	3.8063	3.4591	4.5068	4.7864	4.1578	3.5795	0.7619
MAPK8IP2	3.2295	3.4127	3.1412	3.8177	3.7969	3.7222	3.5386	3.4330	3.4412	3.7734	4.1417	3.2920	3.5617	0.2926
CACNA1E	1.4624	2.4444	2.5154	2.7393	2.6572	3.3953	3.4686	2.8109	4.0148	4.7363	4.7312	3.5078	3.2070	0.9672
CACNA1B	2.9298	2.9623	2.8713	3.1239	2.9723	2.9997	3.0819	3.0388	3.1380	2.9230	2.9731	2.9158	2.9942	0.0851
CSF1R	1.4583	1.8618	1.8323	2.9973	4.3021	4.2000	2.5538	1.7181	2.3706	3.7073	3.2951	4.9759	2.9394	1.1622
CACNB1	2.4336	2.4102	3.3055	2.8331	2.9779	3.2038	2.7824	3.0019	2.0781	3.5960	3.7793	2.8565	2.9382	0.4924

CACNB2	2.4474	2.3918	1.9066	2.8493	3.1482	2.9625	3.2487	3.1089	3.1955	3.1722	3.4798	2.7757	2.8906	0.4467
CACNA1S	2.1683	2.3015	2.0553	2.8393	2.9577	3.1752	2.5522	3.3515	2.0496	3.3329	3.1733	3.3858	2.7786	0.5273
EFNA2	2.6704	2.4220	2.3126	3.0278	3.0814	2.7756	3.0882	2.9553	2.3112	3.0742	2.8233	2.4866	2.7524	0.3048
PTPN7	1.8430	1.6282	1.5719	3.2763	2.0513	2.8040	0.9251	3.1327	2.2437	4.3357	2.3549	3.9682	2.5112	1.0198
NGF	2.3688	2.3530	2.3552	2.1207	2.5454	2.3227	2.6541	2.7229	2.6157	2.6579	2.7167	2.6511	2.5070	0.1950
ERBB3	1.1641	2.0160	1.7173	3.0343	3.4412	2.5183	2.6130	2.5870	2.5056	2.4881	2.3896	2.4501	2.4104	0.5832
FGF17	2.3007	1.8706	2.2278	2.7509	2.7077	1.7358	1.7222	2.4972	2.0866	2.6599	2.2156	2.4213	2.2664	0.3610
RASGRP2	2.1261	1.0205	1.7175	1.2456	2.7416	1.2210	1.2553	2.1343	1.7117	4.1326	3.7197	3.3821	2.2007	1.0604
TGFB2	1.8619	1.7116	1.6572	3.2590	2.9185	2.7935	1.2074	1.5873	1.6533	2.8179	2.8363	1.7675	2.1726	0.6916
FLT3	2.0402	0.9765	0.7026	2.1785	2.9982	2.8799	2.6946	1.1731	1.6380	2.1247	2.3058	2.3133	2.0021	0.7410
ANGPT1	1.1522	1.1726	1.6448	1.8830	1.8110	2.1614	1.5710	1.5447	1.7978	2.0573	2.1347	2.1555	1.7572	0.3536
FLT4	1.1029	1.0243	1.6636	1.8137	1.3626	1.5459	1.2442	1.2318	1.5792	2.1730	2.1232	1.9737	1.5698	0.3909
ANGPT2	1.7619	1.5045	1.6281	1.5701	2.2540	1.0733	1.3666	1.7656	1.9683	1.0562	1.0653	1.6965	1.5592	0.3730
KIT	-0.1392	0.4861	0.6579	2.2780	2.7773	2.4263	1.3288	0.6004	0.6547	1.5405	2.4461	2.1043	1.4301	0.9665
RAC2	0.2444	0.0864	0.3305	2.8336	0.7067	2.4190	0.2715	1.2489	1.3415	2.7559	0.6318	2.0501	1.2434	1.0285
IL1A	1.1886	1.1869	1.1897	1.1967	1.1956	1.1963	1.1889	1.1887	1.1896	1.1919	1.1942	1.1914	1.1915	0.0034
RASGRF2	0.9360	0.9287	1.2955	0.9691	1.7623	0.9674	0.9373	0.9363	0.9402	1.3580	0.9590	0.9475	1.0781	0.2612
FGF8	1.1754	1.2038	1.1113	1.2381	0.7613	0.7708	1.0419	0.7325	1.2514	1.3381	1.1697	1.0834	1.0731	0.2074
CACNA2D4	1.0663	0.6893	0.7028	0.7356	1.8409	0.7337	0.6993	0.6980	1.5683	1.8568	0.7241	0.7110	1.0022	0.4705
KITL	1.4558	-0.2109	1.2533	-1.0593	2.1141	-1.0797	1.7541	0.0099	1.5745	1.0249	2.0096	1.7600	0.8839	1.1623
HSPA1A	-0.8252	-0.9436	-0.7608	2.0378	1.9634	1.6046	-0.8050	1.0109	0.5151	1.2969	1.4694	0.6581	0.6018	1.1519
TRP53	-0.0532	-0.2157	0.0346	0.4747	1.5949	0.4532	-0.0255	1.0352	0.0300	0.1939	0.3393	1.8632	0.4771	0.6736
MAP4K1	0.0320	-0.1311	0.1201	3.2517	0.5014	0.5403	0.0598	0.0382	0.1155	0.2800	0.4260	0.2472	0.4567	0.9038
PRKCB	-1.5954	-1.7727	1.2793	2.7443	-1.1061	-1.0667	0.0329	1.4952	1.2727	0.3272	2.0556	1.7992	0.4554	1.5419
FGF22	0.4285	0.4283	0.4287	0.4296	0.4294	0.4295	0.4286	0.4285	0.4287	0.4290	0.4293	0.4289	0.4289	0.0004
PTPN5	0.4824	0.3599	0.3601	0.4758	0.3606	0.3606	0.3601	0.4240	0.4945	0.3603	0.3605	0.3603	0.3966	0.0560
DAXX	-0.1307	-0.2850	-0.0472	2.5927	0.3154	0.3525	-0.1044	-0.1248	1.0674	0.1047	0.2436	0.0734	0.3381	0.7929
NTRK1	0.1371	0.4428	0.3619	0.1448	0.1437	0.1444	0.1374	0.1372	0.1381	0.9245	0.4397	0.5695	0.3101	0.2493
ERBB4	0.3158	0.2295	0.2298	0.2307	0.4969	0.2306	0.2297	0.3165	0.4089	0.2301	0.3637	0.4359	0.3098	0.0962
PDGFB	0.1770	0.1443	0.1963	0.3116	0.2939	0.3052	0.1830	0.1783	0.1953	0.2344	0.2728	0.2263	0.2265	0.0568

MAP3K8	0.0174	-0.0697	0.0663	0.3292	0.2916	0.3157	0.0327	0.0208	0.0638	0.1583	0.2454	1.1787	0.2208	0.3299
CACNG8	0.0946	0.0900	0.0973	0.1151	0.1122	0.1140	0.0954	0.0947	0.0972	0.1029	0.1088	0.1017	0.1020	0.0086
FGFR2	0.0862	0.0433	0.1108	-0.0060	0.0575	0.0671	-0.0366	-0.0913	-0.0809	0.1130	0.2006	0.1859	0.0541	0.0944
FAS	-0.1168	-0.1952	-0.0726	0.1666	0.1322	0.1542	-0.1030	-0.1137	-0.0749	0.0107	0.0901	-0.0067	-0.0108	0.1214
CACNA1I	-1.1856	0.0232	-1.1105	-0.7366	1.4380	-0.7548	-1.1619	-1.1803	0.9526	1.8917	-0.8512	1.1188	-0.1297	1.1608
PDGFD	-0.1427	-0.2440	-0.1321	-0.2427	0.1730	-0.2428	-0.0488	-0.1420	-0.2437	-0.2434	-0.2431	-0.2434	-0.1663	0.1253
RASGRF1	-0.3631	-0.3153	-0.2400	-0.0276	-0.3629	-0.0335	-0.2479	-0.1560	-0.1392	0.0270	-0.3629	-0.0614	-0.1902	0.1445
CACNA1H	-0.9187	0.2053	1.1904	1.1162	-0.4105	-0.3692	0.4607	-0.9119	-0.8268	-0.6477	-0.4909	-0.6833	-0.1905	0.7552
CACNG7	-0.2463	-0.2778	-0.2277	-0.1182	-0.1349	-0.1242	-0.2405	-0.2450	-0.2287	-0.1913	-0.1549	-0.1991	-0.1991	0.0541
RASGRP1	1.0949	-1.6287	-1.4141	-1.0447	-1.0947	-1.0626	1.4441	0.0279	-1.4180	0.3168	1.1765	0.9493	-0.2211	1.1742
CACNG5	-0.3950	-0.2934	-0.3863	-0.3311	0.0497	-0.4777	-0.4783	-0.1513	-0.3868	-0.1347	-0.0905	0.0068	-0.2557	0.1845
IL1R1	-0.4429	-0.4433	-0.4427	-0.4412	-0.4414	-0.4413	-0.4428	-0.4429	-0.4427	-0.4422	-0.4417	-0.4423	-0.4423	0.0007
FGF6	-0.6210	-0.6211	-0.6209	-0.4552	-0.3304	-0.6205	-0.6210	-0.6210	-0.6209	-0.3901	-0.6206	-0.6208	-0.5636	0.1069
AREG	-0.5477	-0.6548	-0.5754	-0.5576	-0.7427	-0.5615	-0.3791	-0.5465	-0.4528	-0.6038	-0.6563	-0.5522	-0.5692	0.0941
PGF	-0.5961	-0.5961	-0.5960	-0.5958	-0.5959	-0.5958	-0.5961	-0.5961	-0.5960	-0.5960	-0.5959	-0.5960	-0.5960	0.0001
KDR	-0.4652	-0.5896	-1.4095	-1.3321	-1.3440	-1.3364	0.9616	-0.4603	-1.4102	0.6054	-0.1426	-0.2925	-0.6013	0.8077
TNF	-1.1481	-1.2008	-1.1183	1.3525	-0.9788	-0.9637	-1.1388	-1.1461	-1.1199	0.6942	0.2832	-1.0736	-0.6299	0.8814
EGF	-0.7318	-0.7929	-0.6968	-0.5020	-0.5304	-0.5122	-0.7209	-0.7294	-0.6986	-0.6298	-0.5651	-0.6439	-0.6462	0.0980
RASGRP4	-0.7956	-0.9056	-0.7344	-0.4138	-0.4588	-0.4299	-0.7764	-0.7913	-0.7376	-0.6208	-0.5145	-0.6444	-0.6519	0.1644
NGFR	-0.7637	-0.7639	-0.7635	-0.7627	-0.5775	-0.7627	-0.5499	-0.7637	-0.7635	-0.6146	-0.7630	-0.7633	-0.7177	0.0838
IGF1	-1.6945	0.3299	-1.6221	-1.2663	1.1370	-1.2834	-0.0902	-1.6893	-1.6258	0.8570	-1.3745	-1.5186	-0.8201	1.0668
FGF21	0.1598	-1.5044	-1.2332	0.8541	-0.8353	-0.7955	-1.2976	-1.3207	-1.2381	-1.0645	-0.9129	-1.0989	-0.8573	0.6866
CD14	-1.0762	-1.1002	-1.0622	-0.9798	-0.9923	-0.9843	-1.0719	-1.0752	-1.0629	-1.0346	-1.0073	-1.0405	-1.0406	0.0408
FASL	-1.9894	-2.0972	-1.9314	0.8959	0.1285	0.1717	-1.9711	-0.3863	-1.9344	-0.1173	-1.7315	-1.8481	-1.0676	1.1063
DUSP9	-1.3934	-1.5373	-1.3163	-0.9350	-0.9867	-0.9535	-1.3690	-1.3880	-1.3202	-1.1771	-1.0513	-1.2056	-1.2195	0.1995
CACNG1	-1.2295	-1.2298	-1.2293	-1.2281	-1.2283	-1.2282	-1.2295	-1.2295	-1.2293	-1.2290	-1.2286	-1.2290	-1.2290	0.0006
PRKCG	-1.5244	-1.6453	-1.4593	-1.1347	-1.1789	-1.1505	-1.5039	-1.5198	-1.4627	-1.3413	-1.2341	-1.3656	-1.3767	0.1693
FGF2	-1.4835	-1.4835	-1.4835	-1.4834	-1.3992	-1.4834	-1.4835	-1.4835	-1.4835	-1.4834	-1.4834	-1.4834	-1.4764	0.0243
NTRK2	-0.5596	-2.7684	-0.4387	0.9878	-2.1694	-2.1350	-2.5791	-2.6001	-2.5252	-2.3701	-2.2368	1.0436	-1.5292	1.4092
FGF20	-1.5602	-1.5202	-1.5602	-1.5601	-1.5602	-1.5602	-1.5602	-1.5602	-1.5602	-1.5602	-1.5602	-1.4980	-1.5517	0.0204

FGF1	-1.7270	-1.7545	-1.7111	-1.6208	-1.6342	-1.6256	-1.7221	-1.7259	-1.7120	-0.5373	-1.6504	-1.6869	-1.5923	0.3353
IL1B	-2.1528	-2.3154	-2.0669	-1.6538	0.2804	-1.6734	-2.1255	-2.1467	-2.0713	-0.0074	-1.7779	-1.9451	-1.6380	0.8553
CACNG6	-1.9289	-2.0758	-1.8509	-1.4717	-1.5225	-1.4898	-1.9042	-1.9234	-1.8549	-1.7113	-1.5863	-1.7398	-1.7550	0.1996
IGF2	-2.1103	-2.1113	-2.1098	-2.1060	-2.1066	-2.1062	-2.1102	-2.1103	-2.1098	-2.1086	-2.1073	-2.1089	-2.1088	0.0018
TGFA	-3.2389	-3.3350	-3.1882	-0.5497	-2.9786	-2.9582	-3.2228	-3.2353	-3.1908	-3.0983	-3.0187	-3.1165	-2.9276	0.7576
FGF16	-3.0169	-3.0169	-3.0169	-3.0168	-3.0168	-3.0168	-3.0169	-3.0169	-3.0169	-3.0169	-3.0168	-3.0169	-3.0169	0.0000
FGF10	-4.0907	-4.1551	-4.0567	-3.7961	-3.8324	-3.8090	-1.7320	-4.0882	-4.0584	-3.9667	-3.8779	-3.9869	-3.7875	0.6586
FGF5	-3.8711	-3.8711	-3.8711	-3.8696	-3.8698	-3.8697	-3.8711	-3.8711	-3.8711	-3.8707	-3.8701	-3.8708	-3.8706	0.0006
PPP3C	#NV	#NV	#NV	#NV	#NV	#NV	#NV	#NV	#NV	#NV	#NV	#NV	#NV	#NV
RPS6KA	#NV	#NV	#NV	#NV	#NV	#NV	#NV	#NV	#NV	#NV	#NV	#NV	#NV	#NV
INS	#NV	#NV	#NV	#NV	#NV	#NV	#NV	#NV	#NV	#NV	#NV	#NV	#NV	#NV
NTF4	#NV	#NV	#NV	#NV	#NV	#NV	#NV	#NV	#NV	#NV	#NV	#NV	#NV	#NV
FGF19	#NV	#NV	#NV	#NV	#NV	#NV	#NV	#NV	#NV	#NV	#NV	#NV	#NV	#NV
FGF4	#NV	#NV	#NV	#NV	#NV	#NV	#NV	#NV	#NV	#NV	#NV	#NV	#NV	#NV

Table S3 Relative expression values of genes related to the JAK-STAT pathway

Jak-Stat	Replicate 1	Replicate 2	Replicate 3	Replicate 4	Replicate 5	Replicate 6	Replicate 7	Replicate 8	Replicate 9	Replicate 10	Replicate 11	Replicate 12	Mean	SD	Comments
GAPDH	13.5720	13.8087	13.5775	12.9286	12.6895	12.9789	13.4230	13.6322	13.6013	13.3205	13.3560	13.2579	13.3455	0.3331	Positive Reference Gen
EPO	-1.0478	-1.0043	-1.0477	-1.0476	-1.0476	-0.9545	-1.0477	-1.0478	-1.0477	-1.0477	-0.9653	-0.9802	-1.0238	0.0370	Negative Reference Gen
MYC	12.9329	12.9820	12.9262	12.9637	12.8473	12.9155	13.3571	13.3971	13.3457	12.8349	12.8262	12.6471	12.9980	0.2391	
IL6ST	11.2228	10.9270	11.3525	11.1044	11.3553	11.1060	11.2427	11.1333	11.1967	11.3824	11.4496	12.1697	11.3035	0.3095	
IL6ST	11.2228	10.9270	11.3525	11.1044	11.3553	11.1060	11.2427	11.1333	11.1967	11.3824	11.4496	12.1697	11.3035	0.3095	
JAK1	11.5773	11.4257	11.5887	10.6087	10.7113	10.7186	11.4969	11.4465	11.3317	10.8689	10.8874	11.3319	11.1662	0.3744	
EP300	10.9889	10.8584	10.9209	11.3725	11.3484	11.2756	11.0002	10.8631	10.9640	11.0928	10.9493	10.9606	11.0496	0.1824	
STAT6	10.9158	10.9566	10.9359	11.2241	11.1721	11.1509	11.0384	11.0000	11.0374	11.0723	11.0628	10.7848	11.0293	0.1220	
PTPN11	11.1605	11.1454	11.1508	10.8788	10.8003	10.7972	11.1091	10.9735	10.8987	11.0770	11.0256	11.3280	11.0288	0.1631	
MTOR	10.5973	10.5724	10.6838	10.6982	10.8748	10.7847	10.7279	10.5986	10.7657	10.6691	10.5977	10.6774	10.6873	0.0905	
PIAS2	10.1654	10.1239	10.3313	10.9776	11.1215	10.9991	10.5065	10.3583	10.5592	11.0799	11.0617	10.5945	10.6566	0.3736	
LIF	10.0614	9.8889	10.0556	10.6286	10.6155	10.5492	9.9326	9.8276	10.0023	11.0117	11.0447	10.5916	10.3508	0.4387	
CCND1	10.5411	10.5323	10.5715	9.2709	9.4322	9.2866	10.7715	10.7688	10.6291	10.0733	9.9975	10.3254	10.1834	0.5685	
TYK2	9.4708	9.4368	9.4972	10.9029	11.1241	10.8681	10.1224	9.9836	10.0975	10.3228	10.2822	9.8309	10.1616	0.5726	
RAF1	10.0856	10.0764	10.0724	10.1959	10.3417	10.2593	10.3624	10.1845	10.1787	10.0367	9.9888	10.0595	10.1535	0.1208	
JAK3	9.1505	9.3149	9.2970	10.7481	10.8176	10.6045	10.0211	9.9810	10.1439	10.3945	10.3180	9.7725	10.0470	0.5707	
STAT5A	9.4213	9.4185	9.4359	10.3488	10.4548	10.4328	9.7669	9.7128	9.6826	10.4730	10.5773	10.3116	10.0030	0.4670	
SOS1	9.6752	9.6241	9.6611	10.2777	10.2333	10.2617	9.8577	9.7789	9.7355	10.1170	10.1319	10.0824	9.9530	0.2544	
AKT1	9.8190	9.7996	9.7243	10.0171	9.9920	10.0747	10.0875	9.8846	9.8976	9.7995	9.7503	9.7493	9.8830	0.1305	
CDKN1A	9.6458	9.7824	9.5567	10.0933	9.9158	10.1105	9.5875	9.5179	9.4519	10.0203	10.0793	10.1111	9.8227	0.2598	
GRB2	9.8187	9.8374	9.8203	9.8006	9.6244	9.7788	9.8335	9.8058	9.7379	9.5354	9.6914	9.7674	9.7543	0.0936	
STAT3	9.7319	9.5343	9.6878	9.4925	9.6590	9.5303	9.8996	9.7554	9.7562	9.8665	9.8390	10.2728	9.7521	0.2112	
CCND3	9.5641	9.5821	9.5033	9.6188	9.4847	9.6795	9.7073	9.7372	9.6409	9.8482	9.8109	10.1526	9.6941	0.1829	
PIAS1	9.3806	9.2822	9.3924	9.8433	9.9047	9.9122	9.6765	9.5876	9.5850	9.7469	9.6825	9.6347	9.6357	0.2050	
MCL1	9.7999	9.8368	9.6727	9.5187	9.7423	9.2923	9.6099	9.5605	9.6360	9.4362	9.3583	9.4518	9.5763	0.1722	

GHR	9.1481	9.0026	9.2189	9.7590	9.8151	9.8556	9.2013	9.1967	9.2409	9.4806	9.5224	9.4400	9.4068	0.2847
JAK2	9.3697	9.3379	9.3876	9.2426	9.3210	9.4307	9.6172	9.5477	9.5603	9.3032	9.2775	9.3329	9.3940	0.1206
STAT1	9.5540	9.4940	9.6007	8.6342	8.8222	8.7768	9.7343	9.7427	9.7142	9.3175	9.2728	9.7646	9.3690	0.4105
STAM	9.4220	9.4147	9.4270	9.1975	9.3775	9.3655	9.2054	9.2373	9.0590	9.2063	9.2668	9.1655	9.2787	0.1200
FHL1	8.6822	8.7170	8.7397	9.7574	9.8604	9.6305	9.3487	9.4155	9.1772	9.3861	9.3775	9.0306	9.2602	0.4007
PIK3CA	8.8941	8.7973	8.9463	9.3578	9.5141	9.3783	9.0681	9.0091	8.9539	9.3110	9.1101	9.0802	9.1184	0.2225
EGFR	7.8056	7.6686	7.8223	11.1868	9.8082	10.9622	8.2306	7.9687	8.2839	9.6179	9.4639	8.7251	8.9620	1.2320
PDGFRA	8.7241	8.4761	8.7502	9.0801	9.0547	9.1926	9.0553	8.9531	9.0290	8.5550	8.5331	9.3950	8.8999	0.2888
PIK3CB	8.9997	8.7413	8.8727	8.6727	8.8009	8.6105	9.2069	9.0518	8.8863	8.9648	8.8451	9.1312	8.8987	0.1807
STAT2	8.1801	8.0058	8.1322	9.7863	9.9756	9.5709	8.4925	8.4230	8.4491	9.1406	9.0545	8.9213	8.8443	0.6703
PIK3R1	8.6038	8.4967	8.5200	8.8517	8.7943	8.8142	8.9309	8.8709	8.8672	8.5985	8.5085	8.7161	8.7144	0.1604
BCL2L1	8.9053	8.9913	8.8030	8.1724	8.1704	8.4201	8.8138	8.7811	8.6396	8.8015	8.8143	9.1500	8.7052	0.3050
PDGFRB	7.9081	7.6439	7.9983	8.4990	8.4875	8.5429	8.7159	8.7071	8.7307	8.8450	8.9618	9.6879	8.5607	0.5376
PIAS4	8.2669	8.3977	8.2401	8.9846	8.9152	8.9213	8.3086	8.4188	8.2584	8.5314	8.5256	8.5967	8.5304	0.2728
IRF9	7.9631	8.0644	8.1038	9.3176	9.2124	9.2036	7.8517	7.7974	8.0574	8.9432	9.0412	8.4389	8.4995	0.5961
PTPN2	8.6895	8.7568	8.6955	8.1062	8.2000	7.9593	8.3878	8.4102	8.3644	8.3049	8.2317	8.2600	8.3638	0.2453
PIAS3	8.2209	8.0219	8.1121	8.5698	8.7631	8.3152	8.5706	8.4070	8.5774	8.1328	8.0420	8.2384	8.3309	0.2440
SOCS4	8.3164	8.0676	8.3367	7.7717	8.2447	7.7546	8.6142	8.4866	8.5714	8.0287	8.0814	8.2257	8.2083	0.2810
PIM1	7.8302	7.7114	7.7621	8.5808	8.3598	8.7591	7.9143	8.0474	7.7799	8.0475	8.0409	8.0532	8.0739	0.3318
IL22RA1	7.6657	7.5205	7.7452	8.7456	8.7942	8.7253	7.7609	7.5656	7.7561	8.1900	8.3033	7.9924	8.0637	0.4771
AOX1	7.1239	6.8350	6.9291	8.4681	8.4956	8.4860	7.4770	7.2291	7.3499	7.6784	7.6205	7.6495	7.6118	0.5897
IL15RA	7.5652	7.5041	7.4988	7.4722	7.3025	7.6538	7.6621	7.6587	7.7151	7.2833	7.4509	7.4057	7.5144	0.1418
HRAS	7.4140	7.5140	7.3319	7.7922	7.5789	8.0662	7.1767	7.4505	7.4151	7.2996	7.4219	7.4002	7.4884	0.2368
SOCS2	6.8810	6.8685	6.6615	7.7951	7.7886	7.8738	6.9769	6.7084	6.9231	7.6912	7.7496	7.6482	7.2972	0.4913
CCND2	7.5130	7.0938	7.6809	6.8046	7.3311	6.6743	7.4737	7.4172	7.0909	7.3664	6.7548	7.6142	7.2346	0.3447
SOCS6	7.1455	7.0761	6.8780	7.3309	7.1203	7.0250	7.2090	7.0074	7.0498	7.2581	7.1696	7.3558	7.1355	0.1396
IL17D	7.6169	7.6299	7.6616	6.8228	6.3509	6.5717	7.1611	7.4121	7.3371	6.5511	6.3623	6.4245	6.9918	0.5308
PDGFA	6.6595	6.6203	6.6195	7.3033	7.2371	7.1159	6.7536	6.8338	6.9157	7.2603	7.1255	7.1333	6.9648	0.2608
SOCS7	6.8641	7.0082	6.9836	6.8966	7.2470	6.7649	7.1288	7.1099	6.9770	6.6634	6.5638	7.0605	6.9390	0.1994
SOCS3	6.1306	6.1852	6.1034	7.5015	7.2946	7.3718	6.7955	6.5477	6.5522	7.3824	7.3018	7.0798	6.8539	0.5338

CISH	5.9286	5.9224	5.7008	6.8508	6.7609	7.0284	6.2786	5.8979	6.1441	7.6091	7.5244	7.0706	6.5597	0.6654
PIK3R3	6.2277	6.0967	6.3126	6.5672	6.4259	6.1771	6.4692	6.4297	6.4149	7.0156	6.6220	6.7702	6.4607	0.2584
IL11	6.4863	6.4022	6.4574	7.0587	7.0107	7.0796	6.1331	5.8063	6.1307	6.2841	6.4141	6.0415	6.4421	0.4152
IL10	6.0780	6.0417	6.3461	5.5006	5.6520	5.7269	6.4842	6.7433	6.6035	6.5250	6.6286	6.3662	6.2247	0.4181
CNTF	5.6403	5.7475	5.6253	6.1777	6.3023	5.9974	5.9329	6.1990	6.3431	6.5762	6.7565	6.1427	6.1201	0.3529
EPOR	4.9448	4.8728	4.8708	6.2295	6.1592	6.1894	4.5646	4.4316	4.6860	6.0033	6.2849	5.6051	5.4035	0.7378
SOCS1	5.5996	5.9443	5.4946	5.2422	5.3179	4.5734	5.5010	5.7206	5.4754	5.3039	5.0684	5.2607	5.3752	0.3451
PRLR	5.2842	5.1700	5.5179	4.9902	4.8006	4.9262	5.7095	5.6794	6.0457	5.3366	5.2019	5.2733	5.3280	0.3593
IL23A	5.1003	5.2700	5.5543	4.9345	5.0860	4.8347	5.3861	5.1876	5.3329	5.5751	5.1035	5.9759	5.2784	0.3145
IL15	4.4586	4.7417	4.7155	5.3087	5.7469	5.7216	5.1355	5.5013	5.6017	5.4131	5.3697	5.0787	5.2327	0.4167
IL7	4.3487	4.3383	4.4969	4.9293	4.8595	5.1858	4.8642	4.8225	4.7976	4.9747	5.0906	4.9894	4.8081	0.2751
PIK3R2	3.6289	4.6680	4.3328	5.2908	5.1629	5.5391	5.0030	4.3836	4.3267	4.6624	4.1408	4.6177	4.6464	0.5367
BCL2	4.7531	4.4508	4.9800	4.2104	3.5989	1.3262	5.4267	5.0220	4.7935	4.7071	4.6780	5.1889	4.4280	1.0859
CSF3	3.9153	4.0267	4.1164	3.0987	3.2838	3.0057	4.6006	4.2432	4.7796	4.6057	4.6770	4.3425	4.0579	0.6227
STAT4	3.0276	2.8889	3.6154	4.8800	4.7949	4.4917	2.7436	2.9382	2.9377	5.5977	5.2164	5.0328	4.0138	1.0832
PTPN6	2.5316	3.4275	3.2863	4.4028	4.7973	3.4082	3.9241	3.5855	3.3977	3.9254	4.1282	3.5803	3.6996	0.5875
PIK3CD	1.9712	2.6169	2.7965	4.1764	4.8065	4.6224	2.8806	3.6242	3.6488	4.1906	3.6600	3.2838	3.5232	0.8528
IL5RA	2.5547	3.2771	2.5259	3.7998	4.1395	4.1209	2.8579	1.7735	2.6755	4.4326	4.4031	3.4448	3.3338	0.8637
IL20RB	2.3945	3.0271	2.9554	4.0996	3.4646	2.8206	2.4164	2.7414	1.4822	3.1112	4.4132	3.3171	3.0203	0.7771
SOCS5	3.2066	2.6918	2.2262	2.2026	2.9738	3.0186	2.9203	2.4502	2.2215	3.1855	3.9584	3.8405	2.9080	0.5913
LIFR	2.1926	0.4572	2.3140	1.3010	3.0385	1.2749	2.6035	1.2639	1.7656	3.0629	3.7400	3.9685	2.2485	1.0813
IL13RA2	2.0319	2.0575	2.1859	1.7592	2.2527	2.0987	1.7118	1.8452	2.0583	2.4049	2.4473	2.3662	2.1016	0.2440
LEPR	1.2507	1.6315	0.8323	2.5274	2.3042	2.7793	1.5490	2.3067	2.4797	1.6906	2.8782	1.6688	1.9915	0.6426
IL21	1.2909	1.1994	1.2262	1.5844	1.5750	1.4393	1.4422	1.4783	1.6267	1.9483	2.3677	1.8076	1.5822	0.3320
IL20	1.3458	1.2288	0.8898	1.2104	1.4066	1.3239	1.5622	1.2848	1.5271	1.5641	1.3530	1.3188	1.3346	0.1845
IL12RB2	1.0289	1.0162	1.0366	1.0855	1.0777	1.0827	1.0313	1.0295	1.0362	1.0523	1.0685	1.0489	1.0495	0.0237
STAT5B	0.0817	-0.0587	1.7569	2.2996	0.5009	0.5364	0.1059	0.0871	0.1548	1.9537	2.1328	1.9133	0.9554	0.9554
IL12RB1	0.8749	0.8468	0.8916	0.9929	0.9772	0.9873	0.8800	0.8760	0.8907	0.9248	0.9585	0.9177	0.9182	0.0497
GH	0.8069	0.4241	0.0184	0.7824	1.4101	0.7696	1.2436	0.5015	0.0180	1.2288	1.8797	1.4014	0.8737	0.5776
IFNAR2	0.1160	0.9537	0.2036	3.6672	0.5839	0.6227	1.1851	1.1590	0.1991	0.3630	0.5085	0.3302	0.8243	0.9664

IFNAR1	0.1104	-0.0496	0.1972	3.4889	0.5746	0.6132	1.1787	0.1165	1.2458	0.3552	1.6179	0.3226	0.8143	0.9899
CTF1	0.8539	0.7327	0.7654	0.6391	0.6388	0.6390	0.7568	0.6366	0.6369	1.0268	0.8163	0.7849	0.7439	0.1190
IL2RG	1.1359	0.9379	0.1850	2.3569	0.5644	0.6032	0.1252	0.1039	0.1805	0.3439	0.4892	0.3112	0.6114	0.6374
IL6RA	-0.0359	-0.2008	0.0532	3.7491	0.4387	0.4780	-0.0078	-0.0296	0.0486	0.2150	0.3625	1.3028	0.5311	1.0877
IL5	0.3460	0.3459	0.3461	0.3466	0.3465	0.3465	0.3460	0.3460	0.3461	0.3462	0.3464	0.3462	0.3462	0.0002
IL10RB	-0.0361	-0.1966	0.0506	2.2691	0.4265	0.4648	-0.0088	-0.0300	0.0461	0.2082	0.3521	0.1758	0.3102	0.6492
MPL	0.3287	0.1519	0.2559	0.1533	0.1531	0.3158	0.4099	0.3298	0.4249	0.1526	0.4171	0.2718	0.2804	0.1080
PDGFB	0.1770	0.1443	0.1963	0.3116	0.2939	0.3052	0.1830	0.1783	0.1953	0.2344	0.2728	0.2263	0.2265	0.0568
OSMR	0.0681	0.0629	0.0712	0.0915	0.0882	0.0903	0.0690	0.0683	0.0711	0.0776	0.0844	0.0762	0.0766	0.0098
CSF2RB	-0.5320	-0.7002	1.4059	2.7163	-0.0545	-0.0153	-0.5035	-0.5256	-0.4464	-0.2786	-0.1307	-0.3121	0.0519	1.0009
IFNGR1	-0.1461	-0.3032	-0.0612	0.3658	0.3073	0.3449	-0.1193	-0.1401	-0.0656	0.0933	0.2343	0.0615	0.0476	0.2221
IFNGR2	-0.2683	-0.4192	-0.1866	1.4904	0.1685	0.2049	-0.2426	-0.2626	-0.1908	-0.0378	0.0981	-0.0685	0.0238	0.4998
IL24	-0.0181	-0.0185	-0.0179	-0.0165	-0.0167	-0.0166	-0.0180	-0.0181	-0.0179	0.1269	-0.0170	0.1219	0.0061	0.0553
IL13RA1	-0.9885	0.1638	-0.8950	-0.4355	-0.4976	1.7111	-0.9589	-0.9819	0.4875	1.3619	-0.5753	0.6578	-0.0792	0.9455
OSM	-0.1161	-0.1165	-0.1159	-0.1143	-0.1146	-0.1144	-0.1160	-0.1161	-0.1159	-0.1154	-0.1149	-0.1155	-0.1155	0.0007
GFAP	-0.2691	-0.1348	-0.1742	-0.1170	-0.0126	-0.0031	-0.1804	-0.2691	-0.2690	-0.1559	0.1351	-0.0686	-0.1266	0.1233
IL4RA	-0.3293	-0.4707	-0.2525	0.1358	0.0823	0.1167	-0.3051	-0.3239	-0.2565	-0.1125	0.0158	-0.1414	-0.1534	0.2015
IL12A	-0.4882	-0.5009	-0.4805	-0.4336	-0.4410	-0.4363	-0.4858	-0.4876	-0.4809	-0.4653	-0.4497	-0.4686	-0.4682	0.0229
IL12B	-0.4917	-0.4918	-0.4916	-0.4910	-0.4911	-0.4910	-0.4916	-0.4917	-0.4916	-0.4914	-0.4912	-0.4914	-0.4914	0.0003
IL22	-0.5117	-0.5118	-0.5116	-0.5111	-0.5111	-0.5111	-0.5117	-0.5117	-0.5116	-0.5114	-0.5113	-0.5115	-0.5115	0.0003
IFNLR1	-0.8846	-1.0451	-0.7984	1.0960	-0.4293	-0.3919	-0.8574	-0.8785	-0.8028	-0.6428	-0.5018	-0.6747	-0.5676	0.5606
IL2RA	-0.7584	-0.7810	-0.7450	0.4065	-0.6774	-0.6696	-0.7543	-0.7575	-0.7457	-0.7186	-0.6921	-0.7243	-0.6348	0.3297
EGF	-0.7318	-0.7929	-0.6968	-0.5020	-0.5304	-0.5122	-0.7209	-0.7294	-0.6986	-0.6298	-0.5651	-0.6439	-0.6462	0.0980
IL9R	-0.8178	-0.9208	-0.7613	-0.4700	-0.5106	-0.4845	-0.8001	-0.8139	-0.7643	-0.6573	-0.5609	-0.6788	-0.6867	0.1502
IL21R	-0.8818	-0.9984	-0.8183	-0.4955	-0.5401	-0.5114	-0.8618	-0.8773	-0.8215	-0.7022	-0.5955	-0.7261	-0.7358	0.1672
IL10RA	-1.0386	-1.1731	-0.9661	-0.6036	-0.6532	-0.6213	-1.0157	-1.0335	-0.9698	-0.8346	-0.7150	-0.8616	-0.8739	0.1889
IL27RA	-1.3961	-1.5647	-1.3063	1.5081	-0.9259	-0.8878	-1.3677	-1.3898	-1.3109	-1.1451	-1.0001	-1.1780	-0.9970	0.8155
EPO	-1.0478	-1.0043	-1.0477	-1.0476	-1.0476	-0.9545	-1.0477	-1.0478	-1.0477	-1.0477	-0.9653	-0.9802	-1.0238	0.0370
CTF2	-1.1201	-1.0769	-1.1201	-1.1200	-1.0312	-0.8827	-1.0165	-1.1201	-1.1201	-1.1200	-1.0382	-1.1201	-1.0738	0.0725
LEP	-1.6323	-1.6323	-1.5308	-1.6322	-1.5507	-1.6322	-1.6323	-1.6323	-1.6323	-1.5118	-1.6322	-1.6322	-1.6070	0.0465

IL6	-1.7497	-1.8528	-1.6936	-1.4093	-1.4484	-1.4233	-1.7320	-1.7457	-1.6965	-1.5911	-1.4971	-1.6122	-1.6210	0.1474
IL2RB	-1.8713	-2.0253	-1.7895	-1.3920	-1.4453	-1.4110	-1.8454	-1.8655	-1.7937	-1.6432	-1.5121	-1.6730	-1.6889	0.2092
CNTFR	-1.8432	-1.8432	-1.8432	-1.8431	-1.8431	-1.8431	-1.8432	-1.8432	-1.8432	-1.8431	-1.8431	-1.8432	-1.8432	0.0000
CSF3R	-2.4429	-2.5979	-2.3613	-1.9714	-2.0230	-1.9898	-2.4170	-2.4371	-0.3919	-2.2165	-2.0881	-2.2459	-2.0985	0.5748
IL23R	-2.4325	-2.4325	-2.4325	-2.4325	-2.4325	-2.4325	-2.4325	-2.4325	-2.4325	-2.4325	-2.4325	-2.4325	-2.4325	0.0000
IFNB1	-2.5172	-2.5173	-2.5172	-2.5172	-2.5172	-2.5172	-2.5172	-2.5172	-2.5172	-2.5172	-2.5172	-2.5172	-2.5172	0.0000
IL7R	-2.8376	-2.9468	-2.7798	-2.5028	-2.5395	-2.5159	-2.8193	-2.8335	-2.7828	-2.6771	-2.5858	-2.6980	-2.7099	0.1465
PRL	-2.9419	-2.9419	-2.9419	-2.9419	-2.9419	-2.9419	-2.9419	-2.9419	-2.9419	-2.9419	-2.9419	-2.9419	-2.9419	0.0000
IL22RA2	-3.0698	-3.0727	-3.0681	-3.0574	-3.0591	-3.0581	-3.0693	-3.0697	-3.0682	-3.0646	-3.0611	-3.0654	-3.0653	0.0052
IL20RA	-4.1241	-4.1244	-4.1238	-4.1225	-4.1227	-4.1226	-4.1240	-4.1241	-4.1238	-4.1233	-4.1229	-4.1234	-4.1235	0.0007
IFNE	-4.3526	-4.3529	-4.3524	-4.3515	-4.3516	-4.3515	-4.3525	-4.3526	-4.3524	-4.3520	-4.3517	-4.3521	-4.3521	0.0005
IL2	-4.5697	-4.5698	-4.5696	-4.5693	-4.5693	-4.5693	-4.5697	-4.5697	-4.5696	-4.5695	-4.5694	-4.5695	-4.5695	0.0002
IFNG	#NV	#NV	#NV	#NV	#NV	#NV	#NV	#NV	#NV	#NV	#NV	#NV	#NV	#NV
IL3	#NV	#NV	#NV	#NV	#NV	#NV	#NV	#NV	#NV	#NV	#NV	#NV	#NV	#NV
IL3RA	#NV	#NV	#NV	#NV	#NV	#NV	#NV	#NV	#NV	#NV	#NV	#NV	#NV	#NV
IL11RA	#NV	#NV	#NV	#NV	#NV	#NV	#NV	#NV	#NV	#NV	#NV	#NV	#NV	#NV
CSF2RA	#NV	#NV	#NV	#NV	#NV	#NV	#NV	#NV	#NV	#NV	#NV	#NV	#NV	#NV
TSLPR	#NV	#NV	#NV	#NV	#NV	#NV	#NV	#NV	#NV	#NV	#NV	#NV	#NV	#NV
CSF2	#NV	#NV	#NV	#NV	#NV	#NV	#NV	#NV	#NV	#NV	#NV	#NV	#NV	#NV
IL4	#NV	#NV	#NV	#NV	#NV	#NV	#NV	#NV	#NV	#NV	#NV	#NV	#NV	#NV
IL9	#NV	#NV	#NV	#NV	#NV	#NV	#NV	#NV	#NV	#NV	#NV	#NV	#NV	#NV
IL13	#NV	#NV	#NV	#NV	#NV	#NV	#NV	#NV	#NV	#NV	#NV	#NV	#NV	#NV
TSLP	#NV	#NV	#NV	#NV	#NV	#NV	#NV	#NV	#NV	#NV	#NV	#NV	#NV	#NV
IFNW	#NV	#NV	#NV	#NV	#NV	#NV	#NV	#NV	#NV	#NV	#NV	#NV	#NV	#NV
IFNK	#NV	#NV	#NV	#NV	#NV	#NV	#NV	#NV	#NV	#NV	#NV	#NV	#NV	#NV
IL19	#NV	#NV	#NV	#NV	#NV	#NV	#NV	#NV	#NV	#NV	#NV	#NV	#NV	#NV
IFNL1	#NV	#NV	#NV	#NV	#NV	#NV	#NV	#NV	#NV	#NV	#NV	#NV	#NV	#NV
THPO	#NV	#NV	#NV	#NV	#NV	#NV	#NV	#NV	#NV	#NV	#NV	#NV	#NV	#NV
UPD1	#NV	#NV	#NV	#NV	#NV	#NV	#NV	#NV	#NV	#NV	#NV	#NV	#NV	#NV
DOME	#NV	#NV	#NV	#NV	#NV	#NV	#NV	#NV	#NV	#NV	#NV	#NV	#NV	#NV

IFNL2	#NV	#NV	#NV	#NV	#NV	#NV	#NV	#NV	#NV	#NV	#NV	#NV	#NV	#NV
IFNL3	#NV	#NV	#NV	#NV	#NV	#NV	#NV	#NV	#NV	#NV	#NV	#NV	#NV	#NV
IFNA1	#NV	#NV	#NV	#NV	#NV	#NV	#NV	#NV	#NV	#NV	#NV	#NV	#NV	#NV
IFNA2	#NV	#NV	#NV	#NV	#NV	#NV	#NV	#NV	#NV	#NV	#NV	#NV	#NV	#NV
IFNA3	#NV	#NV	#NV	#NV	#NV	#NV	#NV	#NV	#NV	#NV	#NV	#NV	#NV	#NV
IFNA4	#NV	#NV	#NV	#NV	#NV	#NV	#NV	#NV	#NV	#NV	#NV	#NV	#NV	#NV
IFNA5	#NV	#NV	#NV	#NV	#NV	#NV	#NV	#NV	#NV	#NV	#NV	#NV	#NV	#NV
IFNA6	#NV	#NV	#NV	#NV	#NV	#NV	#NV	#NV	#NV	#NV	#NV	#NV	#NV	#NV
IFNA7	#NV	#NV	#NV	#NV	#NV	#NV	#NV	#NV	#NV	#NV	#NV	#NV	#NV	#NV
IFNA9	#NV	#NV	#NV	#NV	#NV	#NV	#NV	#NV	#NV	#NV	#NV	#NV	#NV	#NV
IFNA10	#NV	#NV	#NV	#NV	#NV	#NV	#NV	#NV	#NV	#NV	#NV	#NV	#NV	#NV
IFNA11	#NV	#NV	#NV	#NV	#NV	#NV	#NV	#NV	#NV	#NV	#NV	#NV	#NV	#NV
IFNA12	#NV	#NV	#NV	#NV	#NV	#NV	#NV	#NV	#NV	#NV	#NV	#NV	#NV	#NV
IFNA14	#NV	#NV	#NV	#NV	#NV	#NV	#NV	#NV	#NV	#NV	#NV	#NV	#NV	#NV
IFNA16	#NV	#NV	#NV	#NV	#NV	#NV	#NV	#NV	#NV	#NV	#NV	#NV	#NV	#NV

Table S4 Relative expression values of genes related to the RAP1 signaling pathway

RAP1 Signaling	Replicate 1	Replicate 2	Replicate 3	Replicate 4	Replicate 5	Replicate 6	Replicate 7	Replicate 8	Replicate 9	Replicate 10	Replicate 11	Replicate 12	Mean	SD	Comments
GAPDH	13.5720	13.8087	13.5775	12.9286	12.6895	12.9789	13.4230	13.6322	13.6013	13.3205	13.3560	13.2579	13.3455	0.3331	Positive Reference Gen
EPO	-1.0478	-1.0043	-1.0477	-1.0476	-1.0476	-0.9545	-1.0477	-1.0478	-1.0477	-1.0477	-0.9653	-0.9802	-1.0238	0.0370	Negative Reference Gen
ACTB	14.5408	14.7824	14.5329	14.1700	13.8537	14.2036	14.6576	14.8902	14.7777	14.2898	14.3090	14.3296	14.4448	0.3051	
ITGB1	14.4833	14.2941	14.5779	13.6967	13.4304	13.8708	13.9368	13.8733	13.9475	13.5261	13.5959	14.4074	13.9700	0.3876	
THBS1	13.5517	13.1452	13.5569	11.9202	12.0535	12.1904	12.9619	12.6039	12.8268	12.8505	12.8744	13.9511	12.8739	0.6247	
GNAS	12.7339	13.0403	12.7163	13.0482	12.7945	12.9934	12.8045	12.9907	13.0270	12.8999	12.9094	12.5265	12.8737	0.1617	
KRIT1	12.1920	12.3139	12.1354	12.5535	12.4198	12.4929	11.9975	12.0153	12.0777	12.3483	12.3649	11.9280	12.2366	0.2075	
CTNNB1	12.3459	12.2765	12.3240	11.8545	11.9285	11.8432	12.0937	12.1030	11.9631	11.7564	11.7252	11.9321	12.0122	0.2154	
TLN1	11.7881	11.6482	11.8078	11.9851	12.0733	12.0522	11.9205	11.7573	11.8043	11.9750	11.9591	12.1871	11.9132	0.1550	
ACTG1	12.2716	12.2346	12.2011	10.7522	10.7483	11.0227	12.1757	12.0687	11.8976	11.4260	11.4816	12.0301	11.6925	0.5846	
RAC1	11.4810	11.5909	11.4031	11.7228	11.7026	11.7549	11.5017	11.5636	11.5211	11.2911	11.3533	11.0922	11.4982	0.1930	
PFN1	11.2595	11.5860	11.2844	11.1511	11.0571	11.1074	11.2346	11.5423	11.4275	11.1939	11.2283	11.0312	11.2586	0.1782	
ADCY7	11.0016	10.7419	11.0553	11.2338	11.4307	11.1182	11.2666	11.0654	11.2127	11.1041	11.1140	11.3097	11.1378	0.1748	
PLCG1	10.6824	10.6275	10.6765	11.5069	11.5242	11.3934	11.0183	10.9004	10.9378	10.6893	10.6747	10.4143	10.9205	0.3711	
PKD1	10.3059	10.1866	10.3937	11.3423	11.4577	11.2283	10.9822	10.7916	10.9251	10.6625	10.6721	10.6695	10.8015	0.4042	
MAPK14	10.3489	10.2936	10.3561	11.1923	11.2290	11.1084	10.5954	10.5394	10.5756	10.6955	10.7439	10.4264	10.6754	0.3327	
CDC42	10.7727	10.8494	10.7354	10.4388	10.3329	10.4526	10.6853	10.7457	10.6397	10.2710	10.3346	10.3976	10.5547	0.2033	
RHOA	10.9041	10.9381	10.8801	9.9411	9.9314	10.0626	10.7125	10.6501	10.4889	10.1121	10.2243	10.5075	10.4461	0.3797	
ARAP3	10.0615	9.9968	10.1266	10.7596	10.8271	10.7171	10.5332	10.4559	10.4751	10.2413	10.2326	10.0226	10.3708	0.2979	
ADCY6	10.1952	9.9904	10.1848	10.6212	10.8198	10.5941	10.3803	10.1553	10.2730	10.0760	10.1635	10.4942	10.3290	0.2531	
VEGFA	9.7805	9.7162	9.8433	11.4271	11.4914	11.2323	9.6843	9.5422	9.7503	10.3565	10.3363	10.0384	10.2666	0.7194	
DOCK4	10.4190	10.2984	10.4777	9.8648	10.0813	9.8700	10.3276	10.2571	10.2639	10.1544	10.1528	10.1524	10.1933	0.1906	
RAPGEF1	10.1978	10.0353	10.0619	10.0026	10.1865	10.0908	10.3736	10.1964	10.1817	10.1657	10.1759	10.5863	10.1879	0.1587	
MET	10.2139	9.8769	10.3383	9.4042	9.5582	9.6294	10.7528	10.6375	10.6722	9.9925	9.9368	10.8485	10.1551	0.4976	
RAF1	10.0856	10.0764	10.0724	10.1959	10.3417	10.2593	10.3624	10.1845	10.1787	10.0367	9.9888	10.0595	10.1535	0.1208	

SIPA1L1	10.4184	10.3691	10.4513	9.6607	9.6588	9.7070	10.4889	10.4461	10.3161	9.8587	9.7493	10.2333	10.1131	0.3509
INSR	10.2067	9.8869	10.2950	9.9354	10.0969	9.9412	9.7433	9.6800	9.7487	10.1873	10.2082	10.9359	10.0721	0.3415
PRKCI	10.0882	10.0107	10.0588	9.9357	9.9243	9.7661	10.3475	10.2611	10.2538	9.8549	9.8571	10.0418	10.0333	0.1807
EPHA2	10.5958	10.3902	10.6848	8.7759	8.7455	9.0197	10.3555	10.2023	10.3339	10.0302	10.0438	11.0603	10.0198	0.7634
CTNND1	10.1831	10.1021	10.1742	9.6831	9.7546	9.7172	10.1240	10.1327	10.1077	9.9995	9.9469	10.0558	9.9984	0.1823
SIPA1L3	10.0682	9.9551	9.9515	9.4848	9.5429	9.5886	9.9745	9.8751	9.8726	10.1763	10.0027	10.5178	9.9175	0.2865
MAP2K3	9.9593	9.9888	9.9151	9.5860	9.5667	9.5562	10.0947	9.9875	9.9723	10.0598	10.1190	10.0890	9.9079	0.2129
VASP	10.0739	10.1178	10.0443	9.8776	9.9725	9.8940	9.9174	9.8142	9.7577	9.6593	9.6073	9.9657	9.8918	0.1593
AKT1	9.8190	9.7996	9.7243	10.0171	9.9920	10.0747	10.0875	9.8846	9.8976	9.7995	9.7503	9.7493	9.8830	0.1305
SRC	9.6971	9.6991	9.6550	10.1607	10.2004	10.1745	9.6917	9.4976	9.5577	10.0742	10.1118	10.0593	9.8816	0.2687
RAP1B	9.9172	9.8073	9.7480	10.0297	9.9214	10.0482	9.8054	9.7660	9.6010	9.8309	9.8272	10.0123	9.8596	0.1316
ADCY9	9.7635	9.4895	9.7874	9.4666	9.6473	9.6920	9.8448	9.6617	9.7885	9.7897	9.6823	10.3319	9.7454	0.2188
ENAH	9.3553	9.3449	9.3475	9.6278	9.5060	9.5830	9.6903	9.7301	9.5747	9.9866	9.9221	9.9841	9.6377	0.2351
MAGI3	9.4349	9.2985	9.5072	9.5549	9.4913	9.4086	10.1580	9.9956	10.1374	9.6267	9.5521	9.4145	9.6316	0.2953
BCAR1	9.5031	9.4612	9.3854	9.3422	9.1433	9.2891	9.6403	9.5429	9.5143	9.6767	9.6424	9.7556	9.4914	0.1781
MRAS	8.8774	8.8499	8.7543	9.9834	9.9178	10.0064	9.1383	9.0664	8.9846	10.1611	10.0805	9.9052	9.4771	0.5679
MAP2K2	9.4592	9.5844	9.4184	9.2923	9.4514	9.3256	9.5387	9.4778	9.4350	9.2165	9.1145	9.3919	9.3921	0.1344
RAPGEF6	9.2964	9.1354	9.4159	9.0978	9.3196	9.1427	9.4591	9.3473	9.4384	9.6879	9.4666	9.5963	9.3669	0.1823
RALB	9.5658	9.4783	9.5065	8.8805	8.9664	9.1491	9.5995	9.4658	9.3074	9.3848	9.2938	9.7622	9.3634	0.2603
F2R	9.5960	9.4303	9.7191	8.8500	8.5320	9.0493	9.6668	9.6148	9.6981	8.8268	8.7184	9.7368	9.2865	0.4551
RAPGEF2	9.2518	9.0596	9.1588	9.3913	9.3328	9.4130	9.3763	9.1927	9.1505	9.2352	9.2494	9.1879	9.2499	0.1092
FGFR1	9.1670	8.9461	9.2649	9.2462	9.2356	9.3703	9.2159	9.1531	9.1281	9.1745	9.2275	9.7014	9.2359	0.1777
PIK3CA	8.8941	8.7973	8.9463	9.3578	9.5141	9.3783	9.0681	9.0091	8.9539	9.3110	9.1101	9.0802	9.1184	0.2225
EGFR	7.8056	7.6686	7.8223	11.1868	9.8082	10.9622	8.2306	7.9687	8.2839	9.6179	9.4639	8.7251	8.9620	1.2320
PDGFRA	8.7241	8.4761	8.7502	9.0801	9.0547	9.1926	9.0553	8.9531	9.0290	8.5550	8.5331	9.3950	8.8999	0.2888
PIK3CB	8.9997	8.7413	8.8727	8.6727	8.8009	8.6105	9.2069	9.0518	8.8863	8.9648	8.8451	9.1312	8.8987	0.1807
IGF1R	8.9767	8.7371	9.1688	8.2517	8.6963	8.1217	9.4528	9.3262	9.3846	8.7205	8.6269	9.0556	8.8766	0.4284
APBB1IP	8.9500	8.9300	8.9910	8.7396	8.5591	8.7714	8.8249	8.8978	8.7967	8.9022	8.8545	8.9989	8.8513	0.1243
PARD3	8.9682	8.9047	8.8660	8.5039	8.5947	8.7035	8.8495	8.6405	8.6044	8.8146	8.7810	9.2299	8.7884	0.1983
PIK3R1	8.6038	8.4967	8.5200	8.8517	8.7943	8.8142	8.9309	8.8709	8.8672	8.5985	8.5085	8.7161	8.7144	0.1604

RALA	8.6141	8.9139	8.7196	8.7535	8.5637	8.6913	8.5218	8.6194	8.5845	8.6180	8.4371	8.6333	8.6392	0.1216
NRAS	8.4296	8.3021	8.5704	8.6368	8.9355	8.4326	8.8141	8.5569	8.6609	8.5084	8.5073	8.5541	8.5757	0.1716
PDGFRB	7.9081	7.6439	7.9983	8.4990	8.4875	8.5429	8.7159	8.7071	8.7307	8.8450	8.9618	9.6879	8.5607	0.5376
PRKCA	8.7510	8.5813	8.7480	8.1014	8.6334	8.0322	8.8775	8.8874	8.8351	8.2237	8.1733	8.6475	8.5410	0.3185
RASSF5	8.3323	8.1761	8.3799	8.3405	8.5795	8.2870	8.5197	8.5631	8.7647	8.7172	8.6580	8.5840	8.4918	0.1852
SIPA1L2	8.5154	8.4098	8.5582	8.0245	8.1640	8.2320	8.6094	8.6987	8.5449	8.3799	8.3224	8.6930	8.4294	0.2131
VEGFB	8.1480	8.2159	8.1707	8.5631	8.2418	8.6062	8.1490	8.4060	8.1513	8.5438	8.4290	8.4300	8.3379	0.1771
PLCB1	8.3471	8.1812	8.4020	8.2838	8.1536	8.2147	8.3052	8.0786	8.1664	8.5558	8.4456	8.6903	8.3187	0.1802
RRAS	8.1006	8.2201	8.0392	8.0818	7.9325	8.1146	8.1691	8.4552	8.3138	8.6474	8.8364	8.7519	8.3052	0.2992
RALGDS	7.4694	7.4247	7.4804	9.1340	9.1055	9.0400	7.8645	7.8430	7.7804	8.3774	8.4331	8.4122	8.1971	0.6465
FARP2	7.7790	7.5384	7.7747	8.2965	8.5423	8.4410	7.9537	7.9668	8.0140	8.6630	8.6780	8.4223	8.1725	0.3830
SIPA1	8.1022	8.1351	8.1174	7.8711	7.9513	8.1992	8.5000	8.4181	8.4367	8.0403	8.0595	8.0878	8.1599	0.1960
BRAF	8.3482	8.0927	8.4053	7.7265	8.1132	7.5837	8.3716	8.4177	8.2294	8.0057	8.1245	8.3650	8.1486	0.2703
GNAO1	7.7517	7.6392	7.7931	8.0437	8.4249	8.0083	8.2542	8.2507	8.2910	8.2322	8.3116	8.3075	8.1090	0.2580
CSF1	7.6546	7.4047	7.7612	7.4425	7.5966	7.4906	8.2458	8.2866	8.1387	8.8076	8.8536	9.5481	8.1026	0.6794
CRK	8.2415	8.2447	8.1111	7.9653	8.0453	7.7432	8.1959	8.1208	8.0657	8.0399	7.8729	8.3325	8.0816	0.1666
ITGA2B	7.4733	7.5226	7.5364	8.2032	8.2516	8.1719	7.9847	8.0022	7.9794	8.1617	7.9771	7.5700	7.9028	0.2944
RAP1A	8.0824	8.2335	7.9169	7.5834	7.4398	7.5450	8.0776	8.1364	7.7978	7.8848	7.9845	8.1375	7.9016	0.2602
VEGFC	8.3031	8.3215	8.4201	7.5995	7.6946	7.5540	7.7655	7.8188	7.8107	7.5469	7.6635	7.9141	7.8677	0.3110
LPAR1	8.3126	7.9780	8.2824	7.3036	6.8225	7.3167	8.4714	8.4995	8.4068	7.2964	7.2892	8.0869	7.8388	0.5916
PDGFC	8.1474	8.0114	8.2657	7.0321	6.8760	7.2214	8.2479	8.3075	8.2457	7.3824	7.2963	7.7752	7.7341	0.5388
GNAQ	7.4144	7.2309	7.2929	7.8035	8.0580	7.9008	7.5548	7.1306	7.4114	8.4441	8.4142	7.9692	7.7187	0.4479
HRAS	7.4140	7.5140	7.3319	7.7922	7.5789	8.0662	7.1767	7.4505	7.4151	7.2996	7.4219	7.4002	7.4884	0.2368
KRAS	7.4382	7.4450	7.7496	7.1054	7.4338	7.0881	7.5359	7.6554	7.7291	7.1562	6.9418	7.1529	7.3693	0.2734
GNAI1	7.4504	7.3790	7.4510	7.0331	7.0509	7.2469	7.6702	7.5712	7.4542	7.2466	7.0173	7.4384	7.3341	0.2153
EFNA1	7.0577	6.8238	7.0950	7.7223	7.6476	7.9223	6.9110	7.0473	6.9742	7.2594	7.3066	7.3331	7.2584	0.3461
EFNA3	6.7881	6.9772	6.8918	7.7464	7.6904	7.7768	6.9795	7.1610	7.2287	7.1090	7.1708	7.1839	7.2253	0.3356
PDGFA	6.6595	6.6203	6.6195	7.3033	7.2371	7.1159	6.7536	6.8338	6.9157	7.2603	7.1255	7.1333	6.9648	0.2608
LAT	5.9678	5.8632	6.1400	6.7823	6.4799	6.2904	6.4293	6.2776	6.3588	6.9725	7.0718	6.9110	6.4621	0.3953
PIK3R3	6.2277	6.0967	6.3126	6.5672	6.4259	6.1771	6.4692	6.4297	6.4149	7.0156	6.6220	6.7702	6.4607	0.2584

EFNA4	6.0418	5.9046	5.9291	7.0521	6.8172	6.6720	6.2893	6.3558	6.4465	6.4357	6.5616	6.3323	6.4032	0.3460
HGF	6.9725	6.7967	6.8641	6.0684	5.8991	6.1470	6.7261	6.3519	6.5987	5.8745	5.3533	6.7558	6.3673	0.5008
RAC3	5.2420	5.2232	5.4698	6.7212	7.2081	6.8744	5.8443	5.6163	6.2306	6.3691	6.4715	5.9845	6.1046	0.6506
ADCY3	5.3982	5.4306	5.5287	6.2951	6.3808	6.1398	5.6108	5.3318	5.3443	6.4668	6.2113	5.5992	5.8114	0.4457
ID1	5.2652	5.7299	5.1419	6.2165	6.1800	6.2177	5.0610	5.4271	5.3969	5.9507	5.8337	5.2416	5.6385	0.4355
FGFR3	5.2883	5.1666	5.1787	5.9480	5.8127	6.0614	5.5358	5.3093	5.1882	5.7754	5.6403	5.7087	5.5511	0.3184
RAPGEF3	4.6925	4.5932	4.7036	5.7925	6.1287	5.9908	5.8029	5.2252	5.6686	5.8700	5.8055	5.5964	5.4892	0.5447
ANGPT4	4.9014	4.8998	4.9744	4.8433	4.4968	4.5045	6.0776	6.0685	6.1136	5.9911	5.9444	6.8120	5.4690	0.7749
FGF18	5.4785	5.7564	5.4444	5.5463	5.5614	5.6129	5.2693	5.2671	5.1824	5.2311	5.3472	5.7394	5.4530	0.1961
PARD6A	5.3043	5.2203	4.9099	5.8070	5.2069	5.5468	5.3967	5.4402	5.1739	5.8373	5.9219	5.5796	5.4454	0.3060
LPAR4	5.6319	5.2831	5.8445	5.0483	5.1436	4.8623	5.6083	5.5141	5.3655	4.6568	4.6770	5.3594	5.2496	0.3822
P2RY1	4.9841	4.8407	5.0966	4.7302	5.0798	5.0388	5.3110	5.6677	5.3694	5.4606	5.5599	5.7880	5.2439	0.3340
FGF9	5.1356	5.1425	5.1356	5.1524	5.1512	5.1520	5.1446	5.1356	5.1548	5.1592	5.2472	5.1583	5.1558	0.0301
EFNA5	5.5005	5.0250	5.5140	4.5666	4.7080	4.3203	5.5543	5.2932	5.1074	5.0955	4.5173	5.8556	5.0881	0.4802
TEK	3.9995	4.2114	3.9940	6.6351	6.5289	6.0084	4.3390	4.1966	4.5574	5.4896	5.4365	4.7504	5.0122	0.9738
RAPGEF5	4.1995	4.1006	4.1137	5.3938	5.2740	5.4302	4.4594	4.6822	4.0483	5.3479	4.9784	5.5537	4.7985	0.5947
PIK3R2	3.6289	4.6680	4.3328	5.2908	5.1629	5.5391	5.0030	4.3836	4.3267	4.6624	4.1408	4.6177	4.6464	0.5367
VEGFD	3.9474	4.0894	3.9265	5.1260	4.5331	4.7595	4.4150	4.2028	5.0016	5.3319	5.1375	4.9825	4.6211	0.5014
LPAR2	3.4218	3.9750	3.6213	4.9367	5.0695	4.6489	4.1313	4.1528	4.3491	4.8135	5.3617	4.8759	4.4465	0.6011
PRKCZ	4.2140	4.1398	4.2502	4.5905	4.3447	4.9955	4.1710	4.1426	4.2020	4.4597	4.9485	4.5735	4.4193	0.3025
PLCE1	3.7487	3.4532	3.5043	4.3425	4.2435	4.6325	4.3622	4.4399	4.2491	4.7195	4.7625	4.8923	4.2792	0.4797
FLT1	3.9782	3.6107	3.8575	4.1140	4.0968	4.0784	4.0004	3.7485	3.9565	4.1514	4.2171	6.7571	4.2139	0.8196
ADCY5	4.0976	3.8783	3.7047	4.0662	4.3354	4.3116	4.1500	4.3088	4.5037	4.2941	4.4795	4.2833	4.2011	0.2352
FGFR4	3.6743	3.5239	3.7393	5.2257	4.9507	4.8286	3.3600	3.2425	3.3720	4.7022	4.8432	4.6625	4.1771	0.7470
FGF23	4.0844	4.0843	4.0845	4.0851	4.0850	4.0851	4.0845	4.0844	4.0845	4.0847	4.0849	4.0847	4.0847	0.0003
RASGRP3	3.2496	2.9353	3.3651	4.9134	4.8750	4.4629	3.6195	3.5906	3.6189	4.2337	4.2462	4.3950	3.9588	0.6483
ADORA2A	3.4735	3.3360	2.8500	4.9422	4.7525	5.1442	3.5115	2.0681	3.4917	4.7814	4.8982	4.2244	3.9561	0.9760
MAP2K1	3.9547	3.9289	4.1485	4.1052	4.2472	3.4520	4.1970	3.9633	3.9114	3.6063	3.6553	3.6886	3.9049	0.2544
RAPGEF4	3.2817	3.3634	3.1433	3.9425	3.9778	2.5071	4.1489	4.3182	4.3755	4.0538	4.0761	4.2201	3.7840	0.5760
FGF7	4.1441	3.9250	4.3831	3.3315	3.5170	3.3995	3.6470	3.6958	3.8711	4.1620	3.7446	3.5239	3.7787	0.3273

RAP1GAP	3.7258	2.9470	2.8096	4.1566	4.1496	3.8865	3.2782	3.3336	3.0543	4.3529	4.1187	3.9491	3.6468	0.5364
MAP2K6	2.1790	2.7186	2.7117	3.8852	3.8099	3.3248	3.6086	3.8063	3.4591	4.5068	4.7864	4.1578	3.5795	0.7619
PIK3CD	1.9712	2.6169	2.7965	4.1764	4.8065	4.6224	2.8806	3.6242	3.6488	4.1906	3.6600	3.2838	3.5232	0.8528
LPAR5	3.2311	3.3866	2.4439	4.3793	3.9337	4.3505	2.7050	3.3250	3.1466	3.1224	3.5477	3.0806	3.3877	0.5910
TIAM1	2.9398	2.4806	3.1633	3.2487	3.7728	4.2255	3.4266	1.3423	2.9372	3.8016	3.7627	3.8259	3.2439	0.7739
CSF1R	1.4583	1.8618	1.8323	2.9973	4.3021	4.2000	2.5538	1.7181	2.3706	3.7073	3.2951	4.9759	2.9394	1.1622
VAV1	1.9211	1.7205	1.5126	4.3285	2.8705	4.0734	1.9552	2.2833	2.3818	4.1326	3.2865	3.7937	2.8550	1.0303
EFNA2	2.6704	2.4220	2.3126	3.0278	3.0814	2.7756	3.0882	2.9553	2.3112	3.0742	2.8233	2.4866	2.7524	0.3048
ITGB2	0.9195	2.3739	1.0274	4.2253	3.5743	3.8864	2.8111	0.9271	2.5207	4.0734	3.7276	2.9002	2.7472	1.2327
NGF	2.3688	2.3530	2.3552	2.1207	2.5454	2.3227	2.6541	2.7229	2.6157	2.6579	2.7167	2.6511	2.5070	0.1950
FGF17	2.3007	1.8706	2.2278	2.7509	2.7077	1.7358	1.7222	2.4972	2.0866	2.6599	2.2156	2.4213	2.2664	0.3610
RASGRP2	2.1261	1.0205	1.7175	1.2456	2.7416	1.2210	1.2553	2.1343	1.7117	4.1326	3.7197	3.3821	2.2007	1.0604
ADORA2B	1.2571	1.5859	1.4891	1.5806	1.8715	2.1243	1.4629	2.0732	2.2038	2.1134	2.0462	1.8650	1.8061	0.3176
EVL	1.5942	-0.1408	0.0782	2.6477	3.1031	2.9164	0.0249	2.4881	2.3613	1.9035	2.4740	1.8626	1.7761	1.1614
ANGPT1	1.1522	1.1726	1.6448	1.8830	1.8110	2.1614	1.5710	1.5447	1.7978	2.0573	2.1347	2.1555	1.7572	0.3536
ITGAM	0.4923	1.6173	1.9743	2.5874	1.0914	1.1405	1.5774	1.8588	2.4262	2.4504	0.9960	1.8363	1.6707	0.6499
FLT4	1.1029	1.0243	1.6636	1.8137	1.3626	1.5459	1.2442	1.2318	1.5792	2.1730	2.1232	1.9737	1.5698	0.3909
ANGPT2	1.7619	1.5045	1.6281	1.5701	2.2540	1.0733	1.3666	1.7656	1.9683	1.0562	1.0653	1.6965	1.5592	0.3730
KIT	-0.1392	0.4861	0.6579	2.2780	2.7773	2.4263	1.3288	0.6004	0.6547	1.5405	2.4461	2.1043	1.4301	0.9665
RAC2	0.2444	0.0864	0.3305	2.8336	0.7067	2.4190	0.2715	1.2489	1.3415	2.7559	0.6318	2.0501	1.2434	1.0285
GRIN1	0.6857	0.5900	0.7399	1.0373	2.0020	1.0217	0.7026	0.6895	1.1244	2.3965	1.6874	1.7477	1.2021	0.6055
FGF8	1.1754	1.2038	1.1113	1.2381	0.7613	0.7708	1.0419	0.7325	1.2514	1.3381	1.1697	1.0834	1.0731	0.2074
ITGAL	-0.0010	-0.1672	0.0888	2.7041	0.4778	1.6863	1.0985	1.6309	0.0842	1.3688	2.1315	1.3286	1.0359	0.9341
KITL	1.4558	-0.2109	1.2533	-1.0593	2.1141	-1.0797	1.7541	0.0099	1.5745	1.0249	2.0096	1.7600	0.8839	1.1623
LPAR3	0.4644	0.4641	0.4646	0.6418	0.4658	0.8968	0.6568	0.7313	0.4646	1.5006	1.2505	1.6515	0.8044	0.4307
F2RL3	0.6784	0.6738	1.0083	0.6997	0.6967	0.6986	0.6793	0.9809	0.6811	0.6870	0.6931	0.6858	0.7386	0.1200
CDH1	0.1773	1.0002	0.8165	0.2584	0.2475	1.0837	0.7814	0.1781	0.1880	0.2113	0.2345	0.8931	0.5058	0.3699
LCP2	1.1271	-0.2280	-1.5033	3.0465	1.3205	-1.0955	-1.5630	-0.0042	1.5757	1.0213	1.6779	0.2677	0.4702	1.4145
PRKCB	-1.5954	-1.7727	1.2793	2.7443	-1.1061	-1.0667	0.0329	1.4952	1.2727	0.3272	2.0556	1.7992	0.4554	1.5419
FGF22	0.4285	0.4283	0.4287	0.4296	0.4294	0.4295	0.4286	0.4285	0.4287	0.4290	0.4293	0.4289	0.4289	0.0004

ITGB3	1.1113	-1.0055	1.2303	-0.2871	1.7388	-0.3091	0.5169	-0.8269	-0.7461	1.8693	-0.4256	1.4009	0.3556	1.0698
ADCY4	0.3502	0.0290	0.0351	0.0502	1.2000	0.0493	0.0335	0.0329	0.3781	0.4372	1.1476	0.4249	0.3473	0.4217
PDGFB	0.1770	0.1443	0.1963	0.3116	0.2939	0.3052	0.1830	0.1783	0.1953	0.2344	0.2728	0.2263	0.2265	0.0568
RGS14	-0.1674	-0.3004	-0.0946	1.5157	0.2263	0.2595	0.9538	-0.1622	-0.0984	0.0390	0.1622	0.0114	0.1954	0.5273
FGFR2	0.0862	0.0433	0.1108	-0.0060	0.0575	0.0671	-0.0366	-0.0913	-0.0809	0.1130	0.2006	0.1859	0.0541	0.0944
ADCY1	0.0428	0.0407	0.0441	0.0527	0.0513	0.0522	0.0432	0.0429	0.0440	0.0468	0.0496	0.0462	0.0464	0.0041
PDGFD	-0.1427	-0.2440	-0.1321	-0.2427	0.1730	-0.2428	-0.0488	-0.1420	-0.2437	-0.2434	-0.2431	-0.2434	-0.1663	0.1253
GRIN2A	-0.2475	-0.3975	-0.5291	-0.5286	-0.2761	-0.0879	-0.2999	-0.1925	-0.2867	0.3069	0.2025	-0.1240	-0.2050	0.2555
FGF6	-0.6210	-0.6211	-0.6209	-0.4552	-0.3304	-0.6205	-0.6210	-0.6210	-0.6209	-0.3901	-0.6206	-0.6208	-0.5636	0.1069
PGF	-0.5961	-0.5961	-0.5960	-0.5958	-0.5959	-0.5958	-0.5961	-0.5961	-0.5960	-0.5960	-0.5959	-0.5960	-0.5960	0.0001
KDR	-0.4652	-0.5896	-1.4095	-1.3321	-1.3440	-1.3364	0.9616	-0.4603	-1.4102	0.6054	-0.1426	-0.2925	-0.6013	0.8077
EGF	-0.7318	-0.7929	-0.6968	-0.5020	-0.5304	-0.5122	-0.7209	-0.7294	-0.6986	-0.6298	-0.5651	-0.6439	-0.6462	0.0980
NGFR	-0.7637	-0.7639	-0.7635	-0.7627	-0.5775	-0.7627	-0.5499	-0.7637	-0.7635	-0.6146	-0.7630	-0.7633	-0.7177	0.0838
IGF1	-1.6945	0.3299	-1.6221	-1.2663	1.1370	-1.2834	-0.0902	-1.6893	-1.6258	0.8570	-1.3745	-1.5186	-0.8201	1.0668
FGF21	0.1598	-1.5044	-1.2332	0.8541	-0.8353	-0.7955	-1.2976	-1.3207	-1.2381	-1.0645	-0.9129	-1.0989	-0.8573	0.6866
CNR1	-1.1010	-1.1011	-1.1010	-1.1007	-1.1007	-1.1007	-1.0245	-1.1010	-1.1010	-1.1009	-1.1008	-1.1009	-1.0945	0.0220
FPR1	-1.0009	-0.2160	-2.5233	-2.3382	-2.3635	-2.3472	0.2732	-2.5575	0.3373	-0.1439	-0.6214	-0.7938	-1.1913	1.1566
ADCY2	-1.3494	-1.2713	-1.2277	-0.8051	-1.0735	-1.3036	-1.2390	-1.1956	-1.4088	-1.4088	-1.4087	-1.3327	-1.2520	0.1728
PRKCG	-1.5244	-1.6453	-1.4593	-1.1347	-1.1789	-1.1505	-1.5039	-1.5198	-1.4627	-1.3413	-1.2341	-1.3656	-1.3767	0.1693
FGF2	-1.4835	-1.4835	-1.4835	-1.4834	-1.3992	-1.4834	-1.4835	-1.4835	-1.4835	-1.4834	-1.4834	-1.4834	-1.4764	0.0243
FGF20	-1.5602	-1.5202	-1.5602	-1.5601	-1.5602	-1.5602	-1.5602	-1.5602	-1.5602	-1.5602	-1.5602	-1.4980	-1.5517	0.0204
GRIN2B	-2.3763	-2.4973	-0.4671	-1.9991	-2.0409	-2.0140	-2.3559	0.1383	-0.4725	-2.1966	-2.0934	-0.3166	-1.5576	0.9679
SKAP1	-1.7305	-1.8670	-1.6575	-1.2995	-1.3478	-1.3167	-1.7074	-1.7253	-1.6613	-1.5264	-1.4083	-1.5533	-1.5667	0.1878
FGF1	-1.7270	-1.7545	-1.7111	-1.6208	-1.6342	-1.6256	-1.7221	-1.7259	-1.7120	-0.5373	-1.6504	-1.6869	-1.5923	0.3353
DRD2	-2.2067	-2.2078	-2.2061	-2.2019	-2.2026	-2.2021	-2.2065	-2.2067	-2.2061	-2.2048	-2.2034	-2.2051	-2.2050	0.0020
FYB	-2.6077	-2.7711	-2.5220	-2.1154	-2.1689	-2.1345	-2.5805	-2.6016	-2.5264	-2.3704	-2.2366	-2.4012	-2.4197	0.2159
ADCY8	-2.6024	-2.6024	-2.6023	-2.6023	-2.6023	-2.6023	-2.6024	-2.6024	-2.6024	-2.6023	-2.6023	-2.6023	-2.6023	0.0000
FGF16	-3.0169	-3.0169	-3.0169	-3.0168	-3.0168	-3.0168	-3.0169	-3.0169	-3.0169	-3.0169	-3.0168	-3.0169	-3.0169	0.0000
FGF10	-4.0907	-4.1551	-4.0567	-3.7961	-3.8324	-3.8090	-1.7320	-4.0882	-4.0584	-3.9667	-3.8779	-3.9869	-3.7875	0.6586
FGF5	-3.8711	-3.8711	-3.8711	-3.8696	-3.8698	-3.8697	-3.8711	-3.8711	-3.8711	-3.8707	-3.8701	-3.8708	-3.8706	0.0006

FGF15	-4.3022	-4.3060	-4.3000	-4.2891	-4.2906	-4.2896	-4.3015	-4.3020	-4.3001	-4.2960	-4.2924	-4.2968	-4.2972	0.0056
FGF3	-4.3405	-4.3408	-4.3404	-4.3397	-4.3398	-4.3397	-4.3405	-4.3405	-4.3404	-4.3401	-4.3399	-4.3402	-4.3402	0.0004
CALM	#NV	#NV	#NV	#NV	#NV	#NV	#NV	#NV	#NV	#NV	#NV	#NV	#NV	#NV
NGFB	#NV	#NV	#NV	#NV	#NV	#NV	#NV	#NV	#NV	#NV	#NV	#NV	#NV	#NV
ERK1	#NV	#NV	#NV	#NV	#NV	#NV	#NV	#NV	#NV	#NV	#NV	#NV	#NV	#NV
INS	#NV	#NV	#NV	#NV	#NV	#NV	#NV	#NV	#NV	#NV	#NV	#NV	#NV	#NV
AIP1	#NV	#NV	#NV	#NV	#NV	#NV	#NV	#NV	#NV	#NV	#NV	#NV	#NV	#NV
AIP3	#NV	#NV	#NV	#NV	#NV	#NV	#NV	#NV	#NV	#NV	#NV	#NV	#NV	#NV
AF6	#NV	#NV	#NV	#NV	#NV	#NV	#NV	#NV	#NV	#NV	#NV	#NV	#NV	#NV
TRAV	#NV	#NV	#NV	#NV	#NV	#NV	#NV	#NV	#NV	#NV	#NV	#NV	#NV	#NV
TRBV	#NV	#NV	#NV	#NV	#NV	#NV	#NV	#NV	#NV	#NV	#NV	#NV	#NV	#NV
FGF19	#NV	#NV	#NV	#NV	#NV	#NV	#NV	#NV	#NV	#NV	#NV	#NV	#NV	#NV
FGF4	#NV	#NV	#NV	#NV	#NV	#NV	#NV	#NV	#NV	#NV	#NV	#NV	#NV	#NV
